# Trichothiodystrophy‐associated MPLKIP maintains DBR1 levels for proper lariat debranching and ectodermal differentiation

**DOI:** 10.15252/emmm.202317973

**Published:** 2023-10-06

**Authors:** Arjan F Theil, Alex Pines, Tuğba Kalayci, José M Heredia‐Genestar, Anja Raams, Marion H Rietveld, Sriram Sridharan, Sabine EJ Tanis, Klaas W Mulder, Nesimi Büyükbabani, Birsen Karaman, Zehra O Uyguner, Hülya Kayserili, Jan HJ Hoeijmakers, Hannes Lans, Jeroen AA Demmers, Joris Pothof, Umut Altunoglu, Abdoelwaheb El Ghalbzouri, Wim Vermeulen

**Affiliations:** ^1^ Department of Molecular Genetics Erasmus MC Cancer Institute Rotterdam The Netherlands; ^2^ Department of Medical Genetics, Istanbul Faculty of Medicine Istanbul University Istanbul Turkey; ^3^ Department of Dermatology Leiden University Medical Center (LUMC) Leiden The Netherlands; ^4^ Cancer Science Institute of Singapore National University of Singapore Singapore Singapore; ^5^ Department of Molecular Developmental Biology, Faculty of Science, Radboud Institute for Molecular Life Sciences Radboud University Nijmegen The Netherlands; ^6^ Department of Pathology, Istanbul Faculty of Medicine Istanbul University Istanbul Turkey; ^7^ Department of Medical Genetics Koc University Hospital Istanbul Turkey; ^8^ Department of Pediatric Basic Sciences, Child Health Institute Istanbul University Istanbul Turkey; ^9^ Department of Medical Genetics Koc University School of Medicine (KUSOM) Istanbul Turkey; ^10^ Institute for Genome Stability in Aging and Disease, CECAD Forschungszentrum University Hospital of Cologne Köln Germany; ^11^ Princess Máxima Center for Pediatric Oncology ONCODE Institute Utrecht The Netherlands; ^12^ Department of Proteomics Erasmus MC Rotterdam The Netherlands

**Keywords:** brittle hair phenotype, epithelial barrier function, mRNA splicing, skin differentiation, TTDN1, Genetics, Gene Therapy & Genetic Disease

## Abstract

The brittle hair syndrome Trichothiodystrophy (TTD) is characterized by variable clinical features, including photosensitivity, ichthyosis, growth retardation, microcephaly, intellectual disability, hypogonadism, and anaemia. TTD‐associated mutations typically cause unstable mutant proteins involved in various steps of gene expression, severely reducing steady‐state mutant protein levels. However, to date, no such link to instability of gene‐expression factors for TTD‐associated mutations in *MPLKIP*/*TTDN1* has been established. Here, we present seven additional TTD individuals with *MPLKIP* mutations from five consanguineous families, with a newly identified *MPLKIP* variant in one family. By mass spectrometry‐based interaction proteomics, we demonstrate that MPLKIP interacts with core splicing factors and the lariat debranching protein DBR1. *MPLKIP*‐deficient primary fibroblasts have reduced steady‐state DBR1 protein levels. Using Human Skin Equivalents (HSEs), we observed impaired keratinocyte differentiation associated with compromised splicing and eventually, an imbalanced proteome affecting skin development and, interestingly, also the immune system. Our data show that MPLKIP, through its DBR1 stabilizing role, is implicated in mRNA splicing, which is of particular importance in highly differentiated tissue.

## Graphical abstract

MPLKIP‐deficiency is one of the causative genes for trichothiodystrophy (TTD), a condition characterized by clinical features associated with ectodermal abnormalities. We used mass spectrometry‐based interaction proteomics to disclose the still unknown biological function of MPLKIP and applied a Human 3D skin model to investigate its role in skin development.

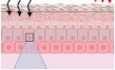

**The paper explained**

**Problem**

Trichothiodystrophy (TTD) is a clinically and genetically heterogeneous disorder characterized by a distinctive brittle hair phenotype, various ectodermal health issues, and developmental and neurologic deficiencies. Despite its identification almost two decades ago, the exact function of one of the TTD‐causative genes, *MPLKIP*/*TTDN1*, associated with a significant number of TTD cases, remains unclear.

**Results**

In this study, we discovered that MPLKIP interacts with core splicing factors and plays a crucial role in maintaining DBR1 protein levels, which is severely reduced in *MPLKIP*‐deficient cells. Using a reconstituted human 3D skin model, we demonstrate that *MPLKIP* deficiency disturbs gene expression, including abnormal pre‐mRNA splicing and altered protein expression. *MPLKIP* deficiency impairs keratinocyte differentiation, resulting in “leaky” skin development and altered immune response.

**Impact**

This study uncovers a biological role of the enigmatic MPLKIP/TTDN1 protein and emphasizes the significant impact of *MPLKIP* deficiency on epithelial differentiation, tissue homeostasis, and immunological skin barrier function, which are commonly affected in TTD. Notably, with the observed down regulation of immune related pathways in *MPLKIP*‐deficient epithelia, future studies may provide potential targets for diagnostic and therapeutic interventions, particularly for addressing recurrent life‐threatening infections associated with TTD.

## Introduction

Trichothiodystrophy (TTD) is a rare recessive multisystem developmental disorder characterized by brittle hair and nails caused by a low content of sulphur‐rich proteins in keratinocytes. Patients present a variable combination of additional symptoms, including photosensitivity, ichthyosis, intellectual disability, reduced fertility, microcephaly, developmental delay, recurrent infections, and anaemia (Faghri *et al*, 2008). The spectrum of clinical features observed in TTD individuals varies from very mild skin symptoms to severe neurologic abnormalities with profound developmental delay and short life expectancy. Most clinical features occur due to defects in highly differentiated cell types such as epithelial tissues. Epithelial barrier function is crucial to sustain tissue homeostasis and to protect against bacterial, viral and fungal infections. “Leaky” epithelia are linked to a variety of diseases and genetic disorders including TTD, and compromise tissue homeostasis, immune response activation and tissue regeneration (Akdis, 2021; Hewitt & Lloyd, 2021; Gutiérrez‐Cerrajero *et al*, 2023).

Almost all affected TTD individuals develop cutaneous manifestations, including ichthyosis, dry skin, palmoplantar keratoderma, atopic dermatitis, follicular keratosis, and keratosis pilaris (Faghri *et al*, 2008). This heterogeneous group of skin conditions is characterized by skin barrier dysfunction, increase in trans‐epidermal water loss and symptoms that can be aggravated by environmental effects (Gutiérrez‐Cerrajero *et al*, 2023). A fully functional epithelial barrier is essential to protect against environmental conditions (e.g., sun‐light exposure, humidity, temperature) and pathogen‐induced infections (Gruber *et al*, 2015), which is a serious concern and common cause of mortality in TTD patients. In fact, approximately two‐third of deaths reported in TTD are a consequence of a chronic infection (Faghri *et al*, 2008; Randall *et al*, 2019). Proper management, timely treatment, and a better understanding of the molecular mechanism are, therefore, necessary.

Approximately 50% of TTD patients is photosensitive, due to biallelic mutations in genes encoding subunits of the dual functional nucleotide excision repair (NER) and basal transcription initiation factor II H (TFIIH), such as the *ERCC2* (or *XPD*) (MIM: 126340), *ERCC3* (or *XPB*) (MIM: 133510) and *GTF2H5* (or TTDA/p8) (MIM: 608780) genes (Stefanini *et al*, 1986; Weeda *et al*, 1997; Giglia‐Mari *et al*, 2004; Compe & Egly, 2012). Photosensitivity in TFIIH‐mutated TTD individuals was shown to be caused by defective NER, which is responsible for the repair of the broad spectrum of base‐pair‐disturbing DNA lesions, including different UV‐induced photoproducts (Stefanini *et al*, 2010; Theil *et al*, 2014), causing sun sensitivity and features of segmental accelerated aging (Schumacher *et al*, 2021). Most of the typical TTD features are related to impaired gene transcription (De Boer *et al*, 1998; Vermeulen *et al*, 2001; Theil *et al*, 2013). Nonphotosensitive TTD (NPS‐TTD) cases carry biallelic mutations in genes *MPLKIP* (MIM: 609188), *RNF113A* (MIM: 300951), *GTF2E2* (MIM: 189964), *CARS1* (MIM: 123859), *TARS1* (MIM: 187790), *MARS1* (MIM: 156560) and *AARS1* (MIM: 601065) (Nakabayashi *et al*, 2005; Corbett *et al*, 2015; Kuschal *et al*, 2016; Theil *et al*, 2017, 2019; Kuo *et al*, 2019; Botta *et al*, 2021), not implicated in DNA repair.

Alterations in basal transcription factor GTF2E2 lead to decreased protein levels of the entire tetrameric TFIIE complex and impaired transcription initiation (Kuschal *et al*, 2016; Theil *et al*, 2017). A nonsense mutation in *RNF113A* in two male cousins with NPS‐TTD causes reduced levels of the encoded ubiquitin‐ligase RNF113A protein (Corbett *et al*, 2015), affecting spliceosome activation (Wu *et al*, 2017). Furthermore, mutations in the *CARS1*, *TARS1*, *MARS1*, and *AARS1* genes, encoding different aminoacyl‐tRNA synthetases (ARS), result in reduced protein abundance and severely impaired enzyme activity, influencing translation (Kuo *et al*, 2019; Theil *et al*, 2019; Botta *et al*, 2021). We postulated that, part of, the TTD phenotype arises from defects at any stage of gene expression; including gene transcription, mRNA splicing, and protein translation (Theil *et al*, 2017). Additionally, TTD‐associated mutant proteins are typically unstable, leading to decreased cellular steady‐state levels of the mutant protein and complex partners (Vermeulen *et al*, 2000; Botta *et al*, 2002, 2021; Corbett *et al*, 2015; Kuschal *et al*, 2016; Theil *et al*, 2017, 2019; Kuo *et al*, 2019). Strikingly, conditions associated with high fever may cause sudden worsening of clinical symptoms in some TTD individuals, such as sudden hair loss, aggravated ataxia, or gastroenteritis (Vermeulen *et al*, 2001; Theil *et al*, 2017; Lanzafame *et al*, 2022), which were due to additional temperature‐dependent decline in protein levels. This thermo‐sensitivity further corroborates that, to some extent, the TTD phenotype develops due to the instability of gene‐expression factors.

Although the gene causative for a significant group of NPS‐TTD cases, *MPLKIP*, originally dubbed as *TTDN1*, was identified almost two decades ago, its exact function has remained unclear to date and thus no such link with gene expression could be made (Nakabayashi *et al*, 2005). MPLKIP is a 179 amino acid protein with a glycine/proline‐rich N‐terminal region, but no obvious functional domains or putative functions have been identified (Heller *et al*, 2015). MPLKIP was previously found to interact with the mitosis‐regulating polo‐like kinase 1 (PLK1), and based on this, it was hypothesized to act in cell cycle progression (Zhang *et al*, 2007). However, cells derived from MPLKIP patients do not display any obvious defects in cell cycle progression, suggesting that it is not essential for cell proliferation and viability despite its important function in promoting health (Heller *et al*, 2015). *MPLKIP*‐deficient individuals also display characteristic skin abnormalities such as dry skin and keratosis pilaris, and suffer from recurrent infections, including gastrointestinal infections, sinopulmonary infections, otitis media, and sepsis (Heller *et al*, 2015; Randall *et al*, 2019).

In this paper, we report on seven new individuals with *MPLKIP* deficiency, each displaying TTD‐associated ectodermal features, which triggered us to closely investigate the biological function of the MPLKIP protein to identify a rational mechanistic basis for this phenotypic expression. We employed mass spectrometry (MS)‐based quantitative MPLKIP interaction proteomics and identified a group of core splicing factors as main MPLKIP interactors. Cellular studies revealed that *MPLKIP* deficiency caused reduced cellular amounts of the splicing‐associated debranching protein DBR1. Within a reconstituted human skin equivalent (HSEs) system, we showed that *MPLKIP* deficiency caused altered gene expression by increased altered spliced transcripts, and altered protein expression, which severely impaired pathways associated with skin development at various stages of terminal keratinocyte differentiation and immune responses. Our data provide novel insights into TTD associated epithelial barrier dysfunction, tissue homeostasis and immunological problems.

## Results

### Sanger sequencing identifies biallelic *MPLKIP* variants in TTD patients

**Figure 1:**
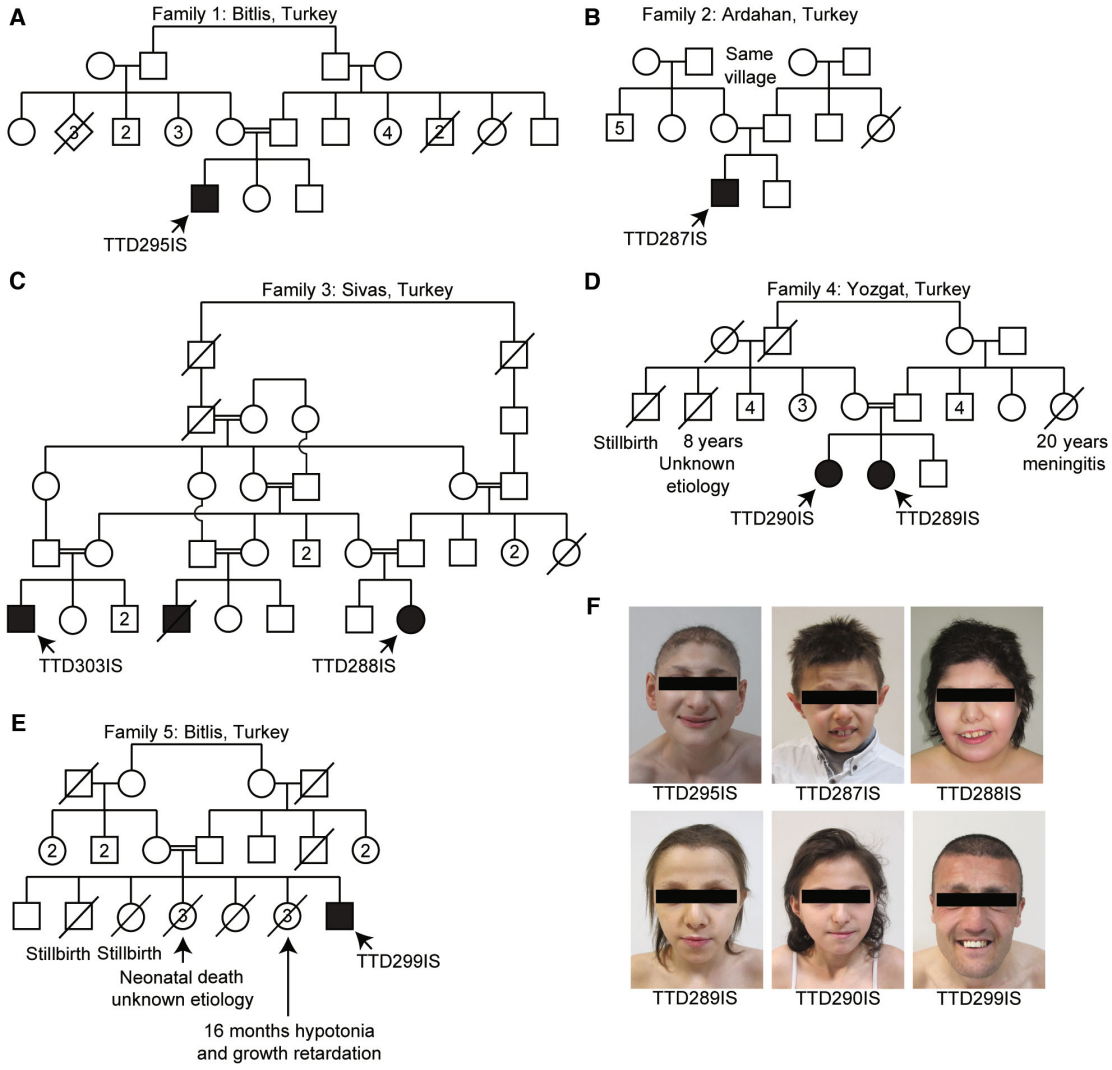
Pedigrees and clinical photographs of TTD individuals A–EThe five pedigrees of seven *MPLKIP*‐deficient individuals. Squares represent males, and circles represent females. Numbers inside symbols indicate the number of unaffected children. Solid circles or squares represent individuals with a TTD diagnosis. Proband is indicated by an arrow and patient number.FFacial views of patient TTD295IS at 14 years and 6 months of age, TTD287IS at 8 years of age, TTD288IS at 13 years and 7 months of age, TTD289IS at 10 years and 10 months of age, TTD290IS at 13 years and 3 months of age, and TTD299IS at 26 years of age. All individuals show similar hair texture (sparse and brittle hair and eyebrows), and facial dysmorphisms (infraorbital creases with thin skin, prominent nasal root, low hanging columella, short philtrum in all cases, and malar hypoplasia except TTD288IS).

**Figure 2:**
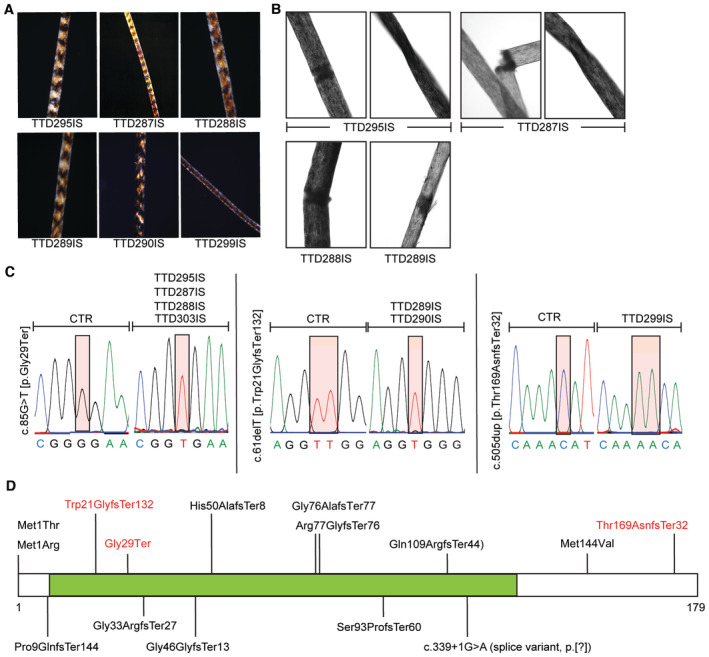
Hair analysis and genetic identification of *MPLKIP* variants AMicroscopic pictures showing the tiger‐tail banding pattern visualized under a polarized light microscope of all TTD individuals.BCharacteristic pictures of hairs with trichorrhexis nodosa, trichoschisis, and pili torti patterns visualized under a light microscope.CRepresentative sequence chromatograms are shown for the individuals indicated at the top. The identified variants and protein alterations are indicated on the left side of each panel. Coloured boxes show the position of the altered nucleotide sequence.DA schematic representation of the domain structure of the MPLKIP protein with the location of previously reported disease‐causing protein alterations. Protein alterations found in our cohort are indicated in red. Source data are available online for this figure.

We investigated a cohort of seven patients from five unrelated families with characteristic features of TTD (Figs 1A–F and 2A and B), including short, woolly, slow‐growing, sparse and brittle hair, sparse and brittle eyebrows and eyelashes, ectodermal abnormalities comprising keratosis pilaris, hyperkeratotic plaques on the scalp, dry skin and nail dystrophy, dysmorphic facial features, microcephaly, global developmental delay and/or intellectual disability, short stature, and hypogonadism. The scalp hair of all affected individuals displayed trichorrhexis nodosa under light microscopy and the characteristic “tiger‐tail” banding pattern under a polarized light microscope (Figs 2A and B). Detailed descriptions of the clinical features are shown in Table EV1 and in Materials and Methods.

**Figure EV1:**
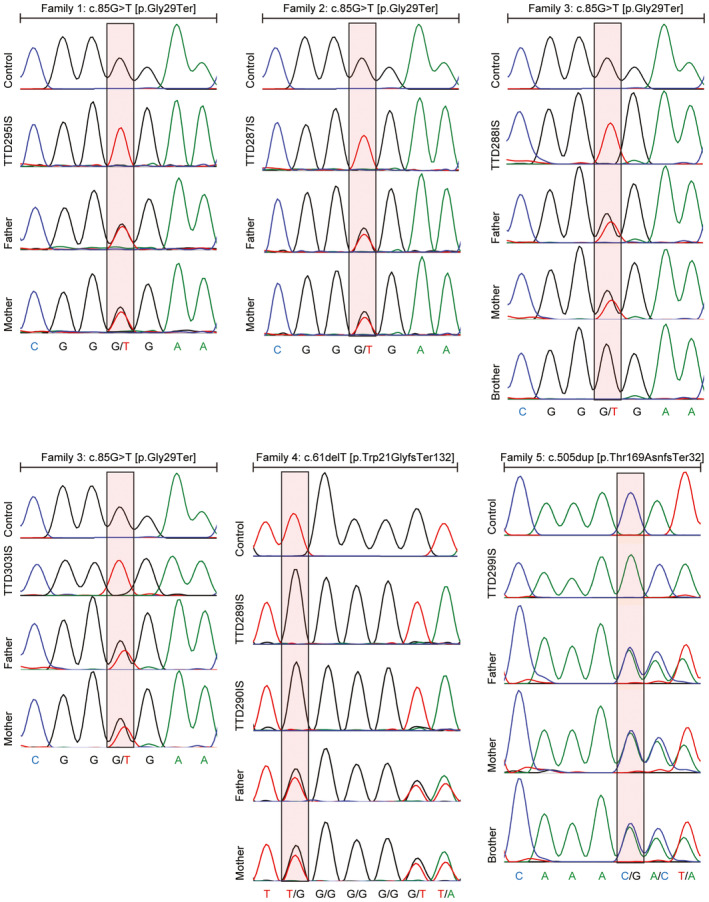
Molecular analysis of *MPLKIP* variants in family members Sequence chromatograms are shown for the indicated family members. Coloured boxes show the position of the altered nucleotide sequence.Source data are available online for this figure.

**Figure EV2:**
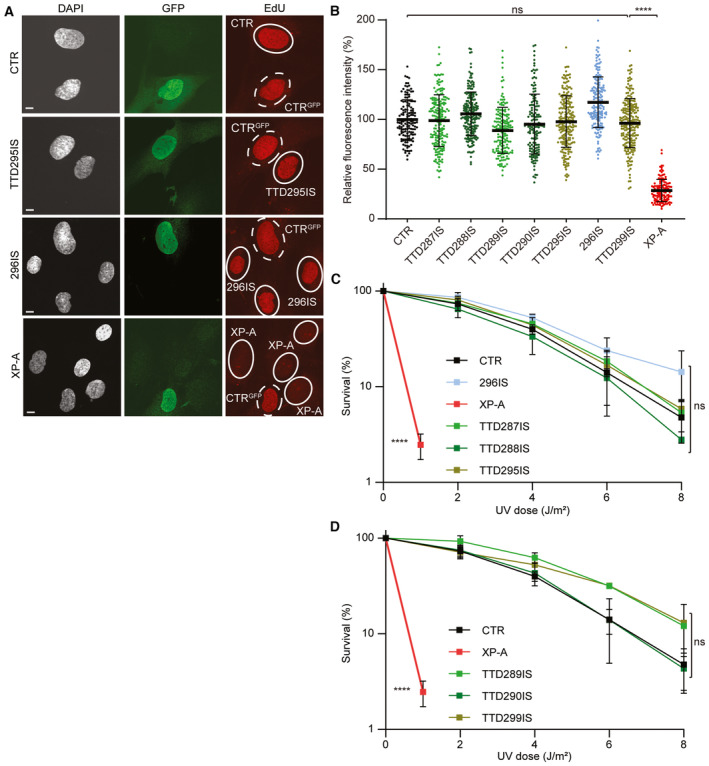
*MPLKIP*‐deficient fibroblasts are DNA repair proficient ARepresentative pictures from the UV‐induced unscheduled DNA synthesis (UDS) experiment performed on primary fibroblasts from MPLKIP‐deficient TTD295IS, the father of TTD295IS (296IS), NER‐deficient XP25RO (XP‐A), and NER‐proficient control (CTR). Wild‐type control fibroblasts that stably express GFP (CTRGFP, dashed circles) were mixed with the test fibroblasts (dashed circles). Global NER activities were measured using EdU incorporation after UV‐irradiation, visualized by fluorescence‐conjugated azide (Click‐iT assay), and subsequently stained for GFP and DNA (DAPI). Scale bars: 20 μm.BMean UDS‐derived fluorescence intensities of at least 50 nuclei were expressed as percentage of the mean intensity in CTR fibroblasts assayed in parallel (*n* = 3 biological replicates).C, DClonogenic UV survival to measure UV sensitivity. One day after seeding, primary fibroblasts were irradiated with the indicated doses of UV, and cultures were incubated for 2 weeks to grow colonies. Survival was blotted as a percentage of colonies obtained after treatment compared to mock‐treated fibroblasts, set at 100% (*n* = 3 biological replicates). Data information: (B). Data are represented as mean ± SD, ordinary one‐way ANOVA. ****P < 0.0001; ns, not significant. (C, D). Data are represented as mean ± SD, nested one‐way ANOVA. ****P < 0.0001; ns, not significant. Source data are available online for this figure.

All individuals carry homozygous, potential deleterious variants in *MPLKIP*, also known as *TTDN1* (Fig 2C; Table EV1), including the novel frameshift NM_138701.4 (MPLKIP):c.61del (p.Trp21GlyfsTer132) in affected siblings TTD289IS and TTD290IS; homozygous nonsense c.85G>T (p.Gly29Ter) in individuals TTD287IS, TTD295IS, and siblings TTD288IS and TTD303IS; and homozygous frameshift NM_138701.4 (MPLKIP):c.505dup (p.Thr169AsnfsTer32) in affected individual TTD299IS. The latter two mutations were previously found to be associated with TTD (Strang‐Karlsson *et al*, 2021). The respective parents were confirmed to be heterozygous for the variants (Fig EV1). All identified mutations are expected to strongly affect the encoded polypeptide and, consequently, severely affect the function of the MPLKIP protein (Fig 2D). Considering allele frequency, predicted pathogenicity of the identified variants, patient phenotypes that are highly specific for TTD, and co‐segregation with disease in multiple family members (Fig EV1), we concluded that the identified variants in *MPLKIP* were likely the cause of the disease. Patient TTD295IS was initially thought to have photosensitive skin features when exposed to sunlight, which is normally only observed in photosensitive TTD individuals with NER deficiency. However, cellular assays to assess NER capacity; Unscheduled DNA synthesis (UDS) (Fig EV2A and B) and clonogenic UV‐light survival assays (Fig EV2C and D) did not reveal NER deficiency in any of the here‐tested TTD patient‐derived fibroblasts. In accordance with these results, clinical follow‐up of patient TTD295IS revealed that ichthyosiform areas on the sun‐exposed areas were mistaken for photosensitivity.

### MPLKIP interacts with core spliceosome factors

**Figure EV3:**
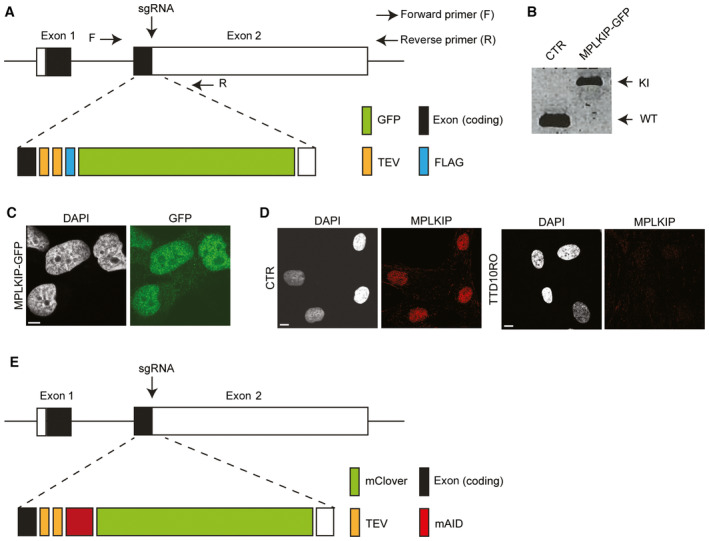
Generation of fluorescently tagged *MPLKIP* knock‐in cells Schematic presentation of a knock‐in strategy to target the coding sequencing of enhanced GFP at the 3′ end of the coding sequencing of the MPLKIP gene using the CRISPR‐Cas9 technology in SV40‐immortalized MRC‐5 cells. The dotted line indicates the DNA fragment TEV(2x)‐FLAG‐GFP targeting construct containing homology arms on both sides. The translational stop codon (in exon 2) was mutated to allow in‐frame fusion with the fluorescent marker and additional FLAG and TEV (2x) tags.PCR amplification of genomic DNA from MRC‐5 (CTR) cells and MRC‐5 MPLKIP‐TEV(2x)‐FLAG‐GFP (MPLKIP‐GFP) knock‐in cells, forward (F) and reversed (R) PCR primers are indicated in panel S2A.Representative immunofluorescence analysis of MRC‐5 cells expressing MPLKIP‐GFP. DNA was stained with DAPI. Scale bars: 10 μmRepresentative immunofluorescence analysis was stained for MPLKIP and DNA (DAPI) to determine steady‐state protein amounts in control fibroblasts (CTR) and MPLKIP‐deficient primary fibroblast TTD10RO. Scale bars: 20 μmSchematic presentation of a knock‐in strategy to target the coding sequencing of mClover in frame fused to the mAID tag at the 3′ end of the coding sequencing of the MPLKIP gene using the CRISPR‐Cas9 technology in HCT116 cells. The dotted line indicates the DNA fragment TEV(2x)‐mAID‐mClover targeting construct containing homology arms on both sides. The translational stop codon (in exon 2) was mutated to allow in‐frame fusion with the fluorescent marker and additional mAID and TEV (2x) tags. Source data are available online for this figure.

To understand the molecular basis underlying the phenotype caused by these MPLKIP variants, it is important to first gain insight into its still unknown biological function. To provide insight into the biological pathway through which MPLKIP is functioning, we examined the MPLKIP protein‐interaction network. To that aim, we inserted the coding sequence of enhanced green fluorescent protein (GFP) at the 3′ end of the coding sequence of the *MPLKIP* gene using CRISPR‐Cas9 technology in SV40‐immortalized MRC‐5 cells (Fig EV3A), to create an in‐frame MPLKIP‐GFP fusion protein, expressed from its endogenous gene locus. The MRC‐5 MPLKIP‐GFP knock‐in cells were genotyped (Fig EV3B) and their sequence verified. The MPLKIP‐GFP fusion protein is predominantly localized in the nucleus (Fig EV3C), which is in line with the observed localization of endogenously expressed untagged MPLKIP proteins in healthy primary fibroblasts (CTR) (Fig EV3D, left panel). As expected, no MPLKIP protein could be detected by immunofluorescence in *MPLKIP*‐deficient primary fibroblasts (TTD10RO; Fig EV3D, right panel), since the *MPLKIP* gene is completely deleted in these primary fibroblasts (Botta *et al*, 2007; Heller *et al*, 2015), which further confirms that the applied antibody truly identifies MPLKIP.

**Figure 3:**
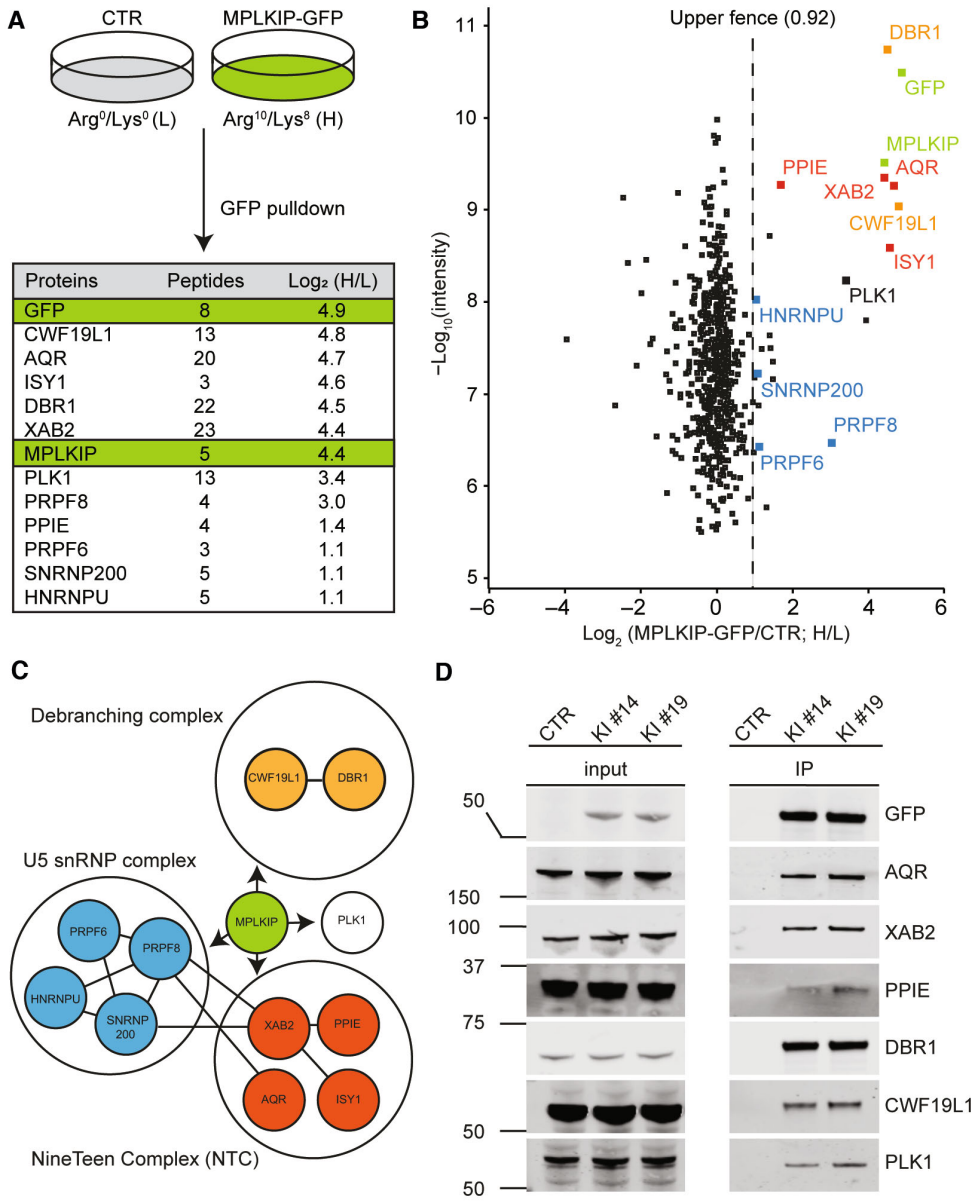
MPLKIP interacts with core spliceosome factors Workflow of the SILAC‐based MS approach. MRC‐5 cells (CTR) were grown in light (L; Arg0/Lys0) medium and MRC‐5 MPLKIP‐GFP knock‐in cells (MPLKIP‐GFP) in heavy (H; Arg10/Lys6) medium. After GFP pull down, proteins were mixed in a 1:1 ratio and analysed by MS. The table shows the number of unique peptides found for the top‐ranked interactors as well as the normalized Log_2_ ratio (H/L; MPLKIP‐GFP/CTR).Scatter plot of Log_2_ SILAC ratios of proteins isolated by GFP‐pulldown in MRC‐5 MPLKIP‐GFP knock‐in cells. The SILAC fold change (Log_2_) is plotted on the x‐axis, and the –Log_10_ signal intensity of the peptides is plotted on the y axis. Gene labels are similarly colour‐coded as in (C), representing the different subcomplexes.STRING protein‐protein interaction analysis. The figure highlights the connections between the most prominently identified interacting proteins.Immunoblotting to validate MS results. MPLKIP‐mAID‐mClover was immunoprecipitated by GFP‐Trap® from two independent HCT116 MPLKIP‐mAID‐mClover knock‐in cells (KI #14 and KI #19), followed by immunostaining for the indicated proteins, left panel input signals, and right panel co‐immunoprecipitated proteins. Source data are available online for this figure.

The GFP tag was exploited as an affinity bait to immune‐purify MPLKIP and its associated proteins. To this end, we performed differential Stable Isotope Labelling by Amino Acids in Cell Culture (SILAC) of MRC‐5 MPLKIP‐GFP knock‐in cells and the MRC‐5 parental control cell line. SILAC‐based quantitative mass spectrometry (MS) was performed on GFP‐pull‐down samples from whole cell extracts of both cell lines (Fig 3A). As the most significant interacting proteins of MPLKIP, we identified PLK1, AQR, XAB2, ISY1, PPIE, DBR1, and CWF19L1 (Fig 3A and B). Previously, PLK1, was also identified as an MPLKIP‐interacting protein (Zhang *et al*, 2007), validating our approach. However, the other identified most significant interacting proteins were novel, and all are surprisingly associated with the core splicing machinery acting at the late stages of pre‐mRNA processing (Fig 3C). For instance, debranching enzyme DBR1 and its interaction partner CWF19L1 are responsible for the turnover of lariat‐intermediates and lariat‐introns, finalizing splicing, and stimulating intron degradation (Chapman & Boeke, 1991; Garrey *et al*, 2014; Montemayor *et al*, 2014). XAB2, ISY1, AQR, and PPIE are all core components of the NineTeen Complex (NTC)/PRP19‐associated complexes, which have been implicated in mediating spliceosome assembly and activation (Makarova *et al*, 2004; Will & Lührmann, 2011; Chanarat & Sträßer, 2013). We used another engineered cell line, HCT116 MPLKIP‐mAID‐mClover (mClover is a GFP‐derivative) knock‐in (Fig EV3E), to verify the strongest MPLKIP‐interactors by immuno‐blotting after GFP pull‐down (Fig 3D). These data not only confirm MPLKIP as a novel spliceosome interactor but also show that this interaction is independent of the cell type.

### Reduced DBR1 protein levels upon MPLKIP depletion in primary fibroblasts

**Figure 4:**
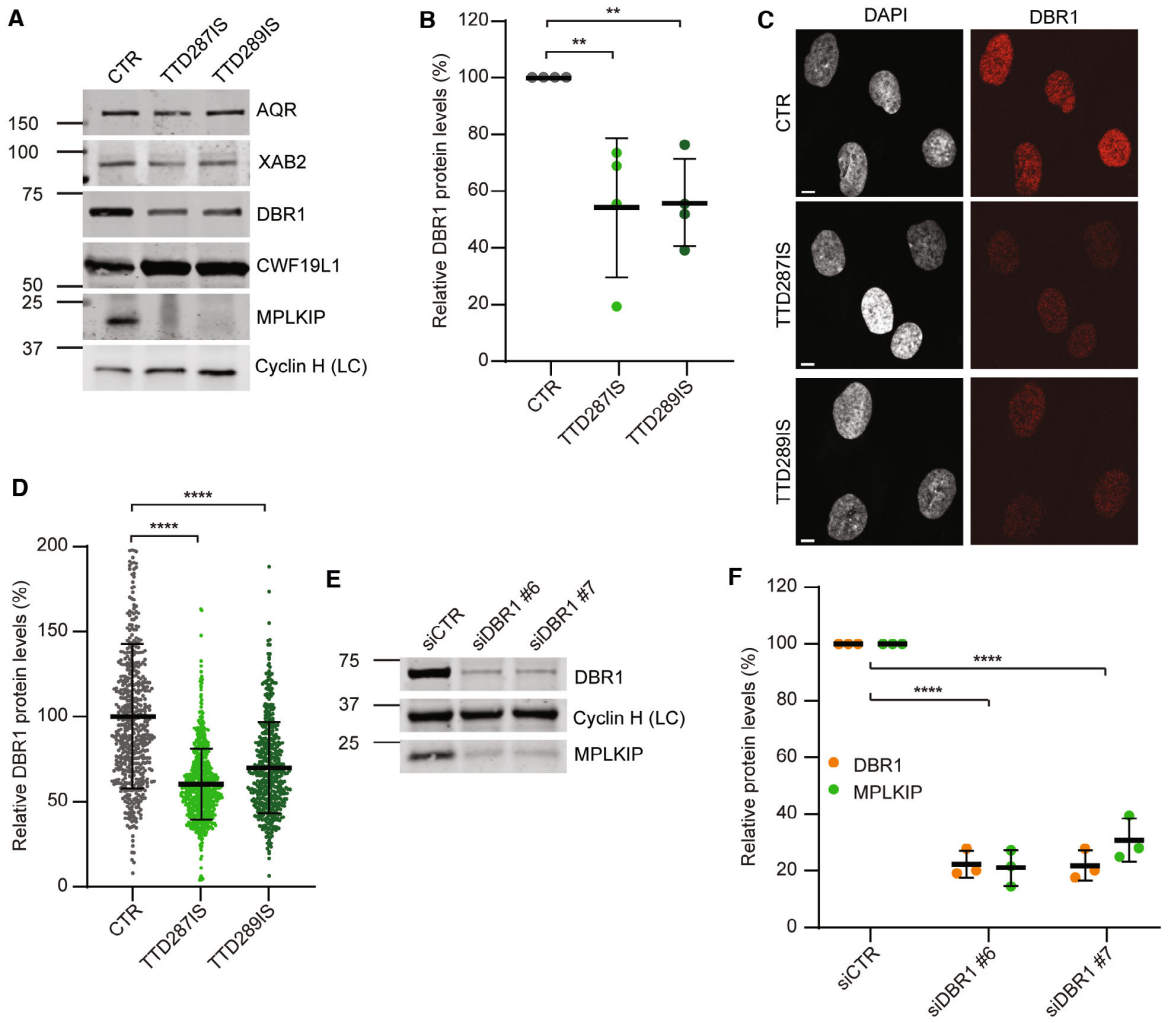
*MPLKIP*‐deficient primary fibroblasts have reduced DBR1 protein levels Representative immunoblot analysis was used to determine steady‐state protein amounts in lysates of *MPLKIP*‐deficient primary fibroblasts (TTD287IS and TTD298IS) and control fibroblasts (CTR), immunostained for the indicated proteins, and cyclin H was used as a loading control.Quantification of the immunoblot analysis. The band intensities of DBR1 were normalized to loading control (LC) Cyclin H and expressed as a percentage of CTR (*n* = 4 biological replicates), set at 100%.Representative immunofluorescence analysis stained for DBR1 and DNA (DAPI) used to determine steady‐state protein amounts in *MPLKIP*‐deficient primary fibroblasts (TTD287IS and TTD298IS) compared with control fibroblasts (CTR). Scale bars: 10 μm.Quantification of the immunofluorescence experiments. Mean fluorescence intensities of at least 150 nuclei were expressed as a percentage of the mean intensity in CTR (*n* = 3 biological replicates), set as 100%.Representative immunoblot analysis used to determine steady‐state protein amounts in lysates of control fibroblasts transfected with control siRNA (siCTRL) or siRNA‐mediated gene knock‐down of DBR1 (siDBR1 #6 and siDBR1 #7).Quantification of the immunoblot analysis. The band intensities of DBR1 and MPLKIP were normalized to loading control (LC) Cyclin H and expressed as percentage of siCTR (*n* = 3 biological replicates), set as 100%. Data information: (B, D, and F). Data are represented as mean ± SD, ordinary one‐way ANOVA. **P < 0.01; ****P < 0.0001. Source data are available online for this figure.

**Figure EV4:**
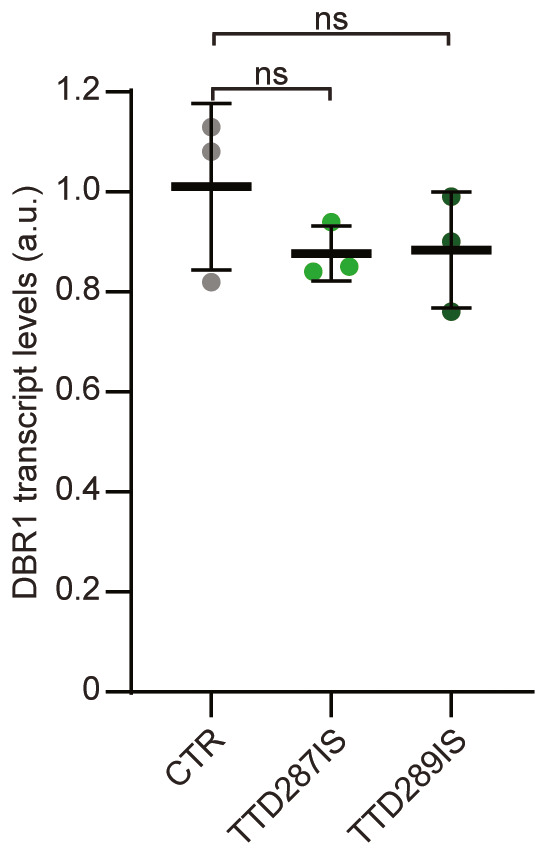
Analysis of *DBR1* transcript levels in *MPLKIP*‐deficient fibroblasts Relative DBR1 transcript levels were assessed by qRT‐PCR in *MPLKIP*‐deficient primary fibroblasts (TTD287IS and TTD298IS) and control fibroblasts (CTR). Total DBR1 transcript levels were first normalized to the levels of *TUBG2* mRNA and then expressed as percentages of the corresponding value in the control fibroblasts (*n* = 3 biological replicates). Data information: Data are represented as mean ± SD, ordinary one‐way ANOVA; ns, not significant. Source data are available online for this figure.

Previous analysis, by us and others (Vermeulen *et al*, 2000; Botta *et al*, 2002, 2021; Corbett *et al*, 2015; Kuschal *et al*, 2016; Kuo *et al*, 2019; Theil *et al*, 2019), revealed that each of the TTD‐causative mutations in other genes than MPLKIP caused instability and/or low steady‐state levels of the mutated protein or even of the protein complexes in which they reside. We postulated that the functional decline in for example, gene expression by this remarkable protein frailty would become especially apparent in highly or terminally differentiated cell types, explaining that phenotypes are observed in specific tissue only. It is however excluded that instability of the MPLKIP protein itself underlies TTD‐specific features, since a significant part of *MPLKIP*‐mutated patients carry null alleles (e.g., large genomic deletions spanning the entire gene) and consequently will not produce any MPLKIP protein at all (Heller *et al*, 2015; Strang‐Karlsson *et al*, 2021). We therefore wondered if *MPLKIP* deficiency might reduce steady‐state protein levels of complex partners of MPLKIP, as previously observed with TFIIE and TFIIH associated TTD mutations. We measured protein content of various MPLKIP interactors in primary fibroblasts from a healthy control (CTR) and *MPLKIP* mutated TTD287IS‐ and TTD288IS‐derived primary fibroblasts. Immuno‐blot analysis showed that MPLKIP deficiency did not change protein levels of closely associated binding partners XAB2, AQR, and CWF19L1. Strikingly, however, steady‐state protein levels of particularly DBR1 were significantly reduced to almost 50% in patient fibroblasts as compared to control primary fibroblasts (Fig 4A and B). Immuno‐fluorescence analysis confirmed these low steady‐state levels of DBR1 in *MPLKIP*‐deficient primary fibroblasts (Fig 4C and D). Real‐time quantitative polymerase chain reaction (RT‐qPCR) analysis demonstrated that total mRNA levels of DBR1 were unaffected in both TTD287IS and TTD289IS primary fibroblasts (Fig EV4), suggesting that reduced DBR1 protein levels are likely caused by protein instability by lacking a functional interaction partner rather than by reduced gene expression. Interestingly, siRNA mediated depletion of DBR1 led to a striking downregulation of MPLKIP protein levels (Fig 4E and F), suggesting a mutual dependency of MPLKIP and DBR1 for protein stability.

### MPLKIP is required for DBR1 binding to the NTC complex

**Figure 5:**
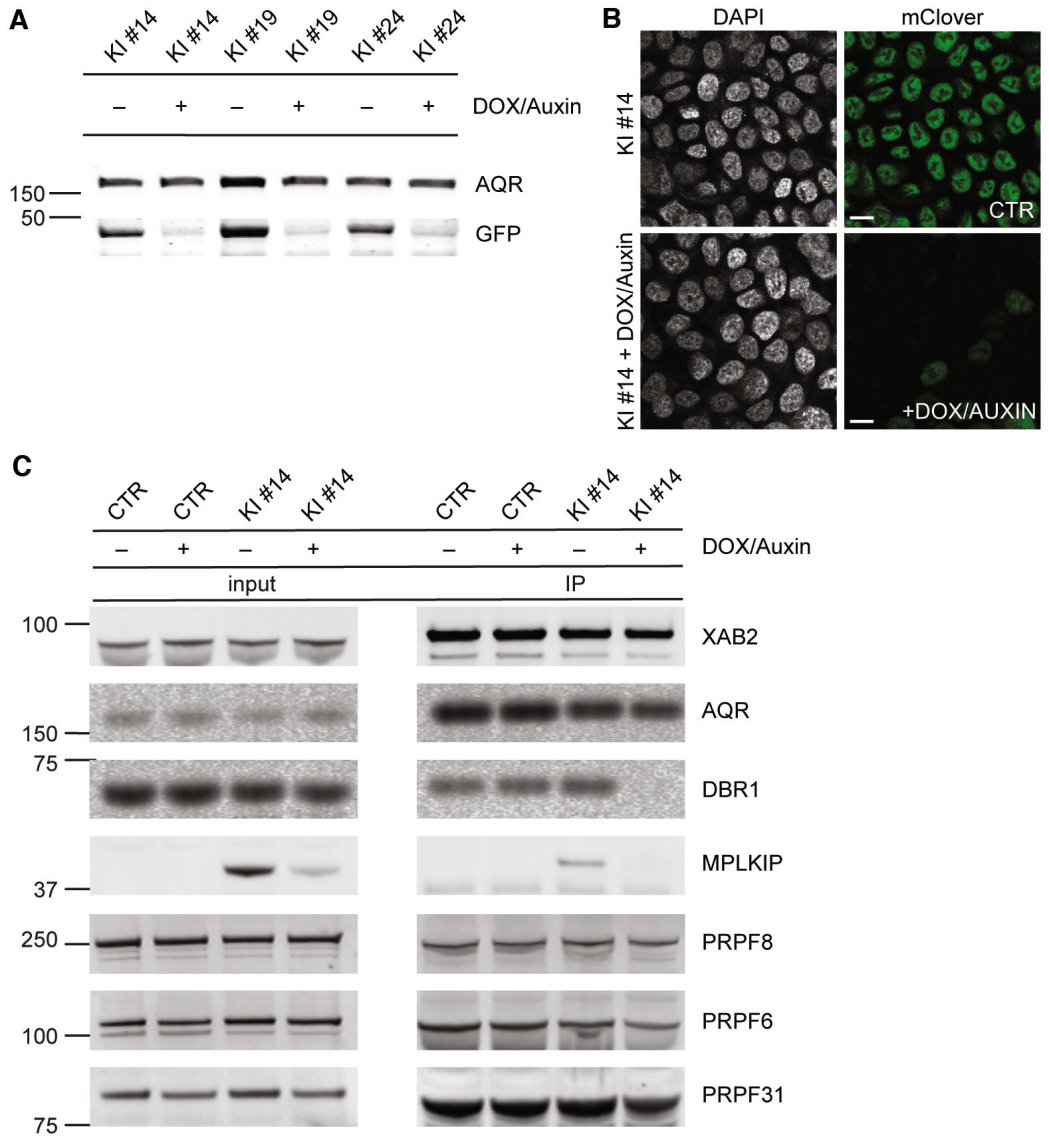
MPLKIP is required for DBR1 binding to the NTC complex ARepresentative Immunoblot analysis showing auxin‐induced (DOX/Auxin) MPLKIP‐mAID‐mClover degradation after 24 h incubation time in three independent HCT116 knock‐in clones (KI #14, KI #19 and KI #24), immunostained for Aquarius (AQR) and GFP (detection of MLKIP‐mAID‐mClover).BRepresentative immunofluorescence analysis stained for DNA (DAPI) and mClover showed auxin‐induced fluorescence loss of MPLKIP‐mAID‐mClover proteins in HCT116 MPLKIP‐mAID‐mClover knock‐in clone (KI #14) after 24 h incubation time. Scale bars: 15 μm.CImmunoblot of XAB2 immunoprecipitation showing the steady‐state complex composition upon auxin‐induced MPLKIP‐mAID‐mClover degradation in the HCT116 control (CTR) and HCT116 MPLKIP‐mAID‐mClover knock‐in clone (KI #14). XAB2 immunoprecipitations was followed by immunoblotting for the indicated proteins. Source data are available online for this figure.

The intricate splicing reaction is a stepwise process involving multiple splicing factors and different snRNP complexes that relies on highly orchestrated complex interactions (Shi, 2017; Wilkinson *et al*, 2020). Since MPLKIP not only interacts with DBR1 but also with components of the NTC, we investigated whether dysfunction or full absence of MPLKIP could compromise the transition of post‐splicing complexes to the debranching complex. We employed mini‐auxin‐inducible degron (mAID) technology to rapidly deplete MPLKIP protein from HCT116 MPLKIP‐mAID‐mClover cells. This approach enabled us to observe an immediate phenotype without affecting DBR1 protein levels (Natsume *et al*, 2016). Immunoblot analysis and immunofluorescence analysis revealed a rapid and efficient depletion (> 90% protein loss) of fluorescent MPLKIP within ~ 2 h after activating the degron system in several independent HCT116 MPLKIP‐mAID‐mClover knock‐in clones (Fig 5A and B). To investigate the interactions between splicing sub‐complexes, we used the XAB2 as an affinity bait to immunoprecipitate the NTC complex and its associated proteins after depletion of MPLKIP‐mAID‐mClover proteins. Transient depletion of MPLKIP‐mAID‐mClover did not significantly change DBR1 protein levels within the experimental timeline (Fig 5C, input lanes), ensuring that we specifically analyse the intrinsic MPLKIP protein function and not secondary aftereffects created by DBR1 depletion. Strikingly, the NTC complex appeared to be less associated with DBR1 upon MPLKIP‐mAID‐mClover depletion (Fig 5C). Collectively, these observations support a scenario in which MPLKIP not only stabilizes DBR1 but also facilitates efficient DBR1 binding to the NTC complex.

### Impaired keratinocyte differentiation in *MPLKIP* ‐deficient TTD human skin equivalents (HSEs)

**Figure 6:**
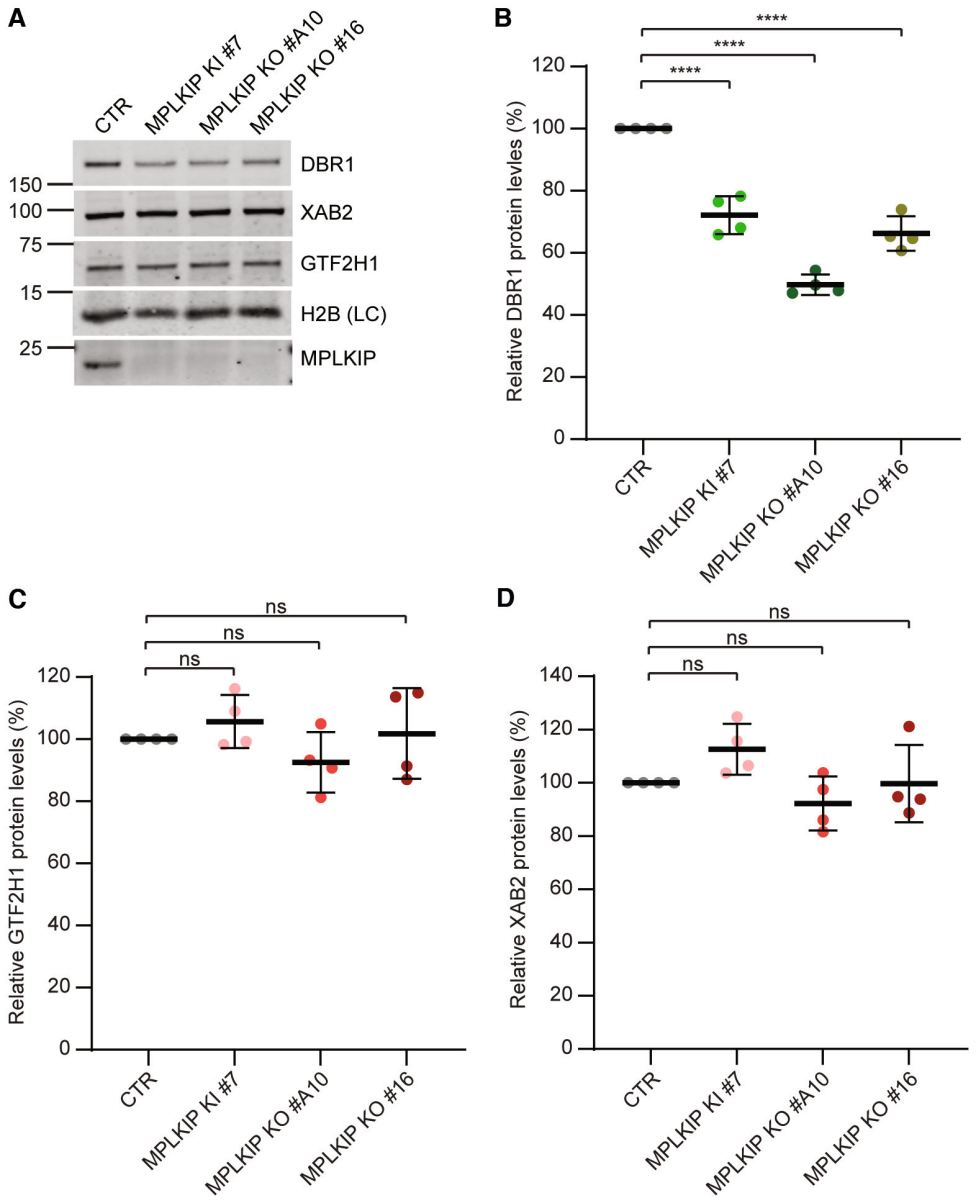
*MPLKIP*‐deficient N/TERT immortalized keratinocytes have reduced DBR1 protein levels ARepresentative immunoblot analysis used to determine steady‐state protein amounts in lysates of *MPLKIP*‐deficient N/TERT‐immortalized primary keratinocytes (KI #7, KO #A10, and KO #16) and control keratinocytes (CTR), immunostained for the indicated proteins.B–DQuantification of the immunoblot analysis. The band intensities of DBR1 (B), GTF2H1 (C) and XAB2 (D) were normalized to loading control (LC) H2B and expressed as percentage of CTR (*n* = 4 biological replicates), set as 100%. Data information: (B–D). Data are represented as mean ± SD, ordinary one‐way ANOVA. ****P < 0.0001; ns: not significant. Source data are available online for this figure.

RNA lariat debranching is the rate‐limiting step in intron turnover (Chapman & Boeke, 1991; Mohanta & Chakrabarti, 2021), which is likely compromised in *MPLKIP*‐deficient cells by the reduced DBR1 protein levels and the affected binding to splicing intermediates. Therefore, *MPLKIP* deficiency is expected to impair splicing with consequent altered spliced transcripts and compromised gene expression. We reasoned that immortalized colon cancer HCT116 cells are not representative cell types to mimic the TTD‐specific symptoms, particularly since these symptoms are mainly apparent in terminally differentiated tissues, such as human skin. Primary human keratinocytes are a more relevant cell type to study *in situ* 2D keratinocyte differentiation (Mulder *et al*, 2012; Tanis *et al*, 2018). To recapitulate epithelial morphogenesis we used human skin equivalents (HSEs) to mimic normal epidermal differentiation with respect to morphology, expression of differentiation markers and lipids (El Ghalbzouri *et al*, 2002; Van Drongelen *et al*, 2014). The *MPLKIP*‐deficient subjects in this study all presented ectodermal abnormalities, including keratosis pilaris, dry skin, and hyperkeratotic plaques on the scalp. These skin abnormalities are likely a consequence of defects in epidermal differentiation that is driven by impaired gene expression and aberrant splicing. To reconstitute HSEs with *MPLKIP* deficiency, we used primary fibroblasts from *MPLKIP*‐deficient individuals (TTD287IS, TTD289IS, and TTD299IS), and CRISPR/Cas9‐mediated *MPLKIP*‐deficient N/TERT keratinocytes. Three independent *MPLKIP*‐deficient N/TERT keratinocyte cell lines were created, two *MPLKIP* knock‐out cell lines (KO #A10 and KO #16) and one *MPLKIP* knock‐in cell line mimicking TTD289IS/TTD290IS mutation (KI #7; p.Trp21GlyfsTer132). MPLKIP inactivation was sequence verified and confirmed by immunoblot analysis, which showed a concomitant reduction in DBR1 protein levels to almost 50% (Fig 6A and B), but without affecting the NTC component XAB2 or TFIIH protein GTF2H1 (Fig 6C and D). H2B was used as loading control.

**Figure 7:**
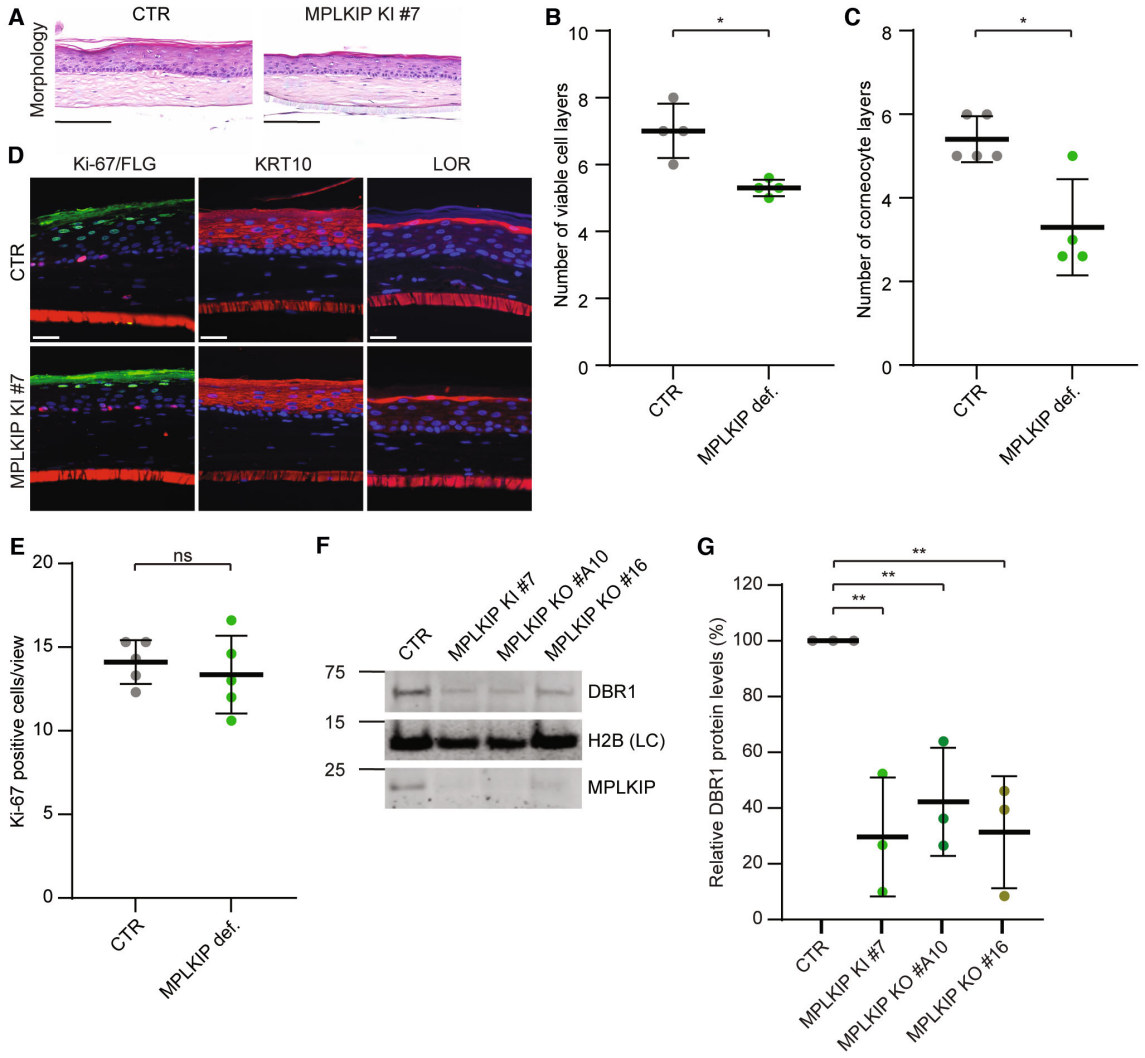
*MPLKIP*‐deficient TTD HSEs exhibit impaired differentiation Representative haematoxylin and eosin (HE)‐stained cross‐sections of *MPLKIP*‐deficient HSE (KI #7) and control HSE (CTR). Scale bars: 100 μm.Quantification of the epidermal thickness based on the HE staining (*n* = 4 biological replicates).Quantification based on safranin red staining of corneocyte layers in the stratum corneum (*n* = 4 biological replicates).Representative immunohistochemistry analysis showing *MPLKIP*‐deficient HSE (KI #7) and control HSE (CTR) stained for DNA (DAPI) (blue channel, all panels), Ki67/FLG (red/green channel, left panel), KRT10 (red channel, middle panel), or LOR (red channel, right panel). Scale bars: 100 μm.Basal cell proliferation was quantified based on the Ki67‐positive cells in multiple regions of the epidermis (*n* = 5 biological replicates).Representative immunoblot analysis to determine steady‐state protein amounts in lysates of *MPLKIP*‐deficient epidermis (KI #7, KO #A10, and KO #16) and control epidermis (CTR), isolated from the HSEs.Quantification of the immunoblot analysis. The band intensities of DBR1 were normalized to loading control (LC) H2B and expressed as percentage of CTR (*n* = 3 biological replicates). Data information: (B, C and E). Data are represented as mean ± SD, Mann–Whitney‐test. *P < 0.05; ns, not significant. (G). Data are represented as mean ± SD, ordinary one‐way ANOVA. **P < 0.01. Source data are available online for this figure.

HSEs were generated by seeding N/TERT keratinocytes onto a dermal collagen substrate containing subject‐derived primary fibroblasts. Thereafter, the HSEs were cultured at the air–liquid interface to initiate epidermal differentiation, as described in more detail in the Materials and Methods section and in (Van Drongelen *et al*, 2014). Haematoxylin and eosin (HE) stained cross‐sections of these HSEs revealed abnormal keratinocyte differentiation (Fig 7A), as compared to control (CTR) HSE reconstituted from wild‐type/parental fibroblasts and keratinocytes. *MPLKIP*‐deficient HSEs were characterized by a general decrease in the number of viable cell layers in the epidermis and corneocyte layers in the stratum corneum (Fig 7B and C). The epidermal morphogenesis was evaluated by determining the presence of the early and two late differentiation markers cytokeratin 10 (KRT10), loricrin (LOR) and filaggrin (FLG), respectively. KRT10 was consistently localized in the suprabasal cells, and the expression of LOR and FLG was restricted to the stratum granulosum (Fig 7D). The proliferation index was determined by counting all Ki67‐positive nuclei, displaying similar proliferative capacity in *MPLKIP*‐deficient HSEs and control (Fig 7E). We also quantified the protein content of DBR1 in epidermal tissue lysates of HSEs deficient in *MPLKIP* (MPLKIP KI #7, KO #A10, and KO #16). Importantly, immunoblot analysis revealed an even more severely reduced steady‐state DBR1 protein level compared to *MPLKIP*‐deficient fibroblasts and N/TERT keratinocytes, to nearly 30% of control (CTR) levels (Fig 7F and G).

Taken together, the evaluation of just a few general protein biomarkers in the HSEs suggests that the potential for epidermal morphogenesis is largely retained in *MPLKIP*‐deficient HSEs. Therefore, the observed abnormal keratinocyte differentiation must be caused by a more subtle defect, likely driven by impaired pre‐mRNA splicing due to indirectly compromised DBR1 protein levels and function.

### Impaired gene expression in *MPLKIP* ‐deficient TTD HSEs

**Figure 8:**
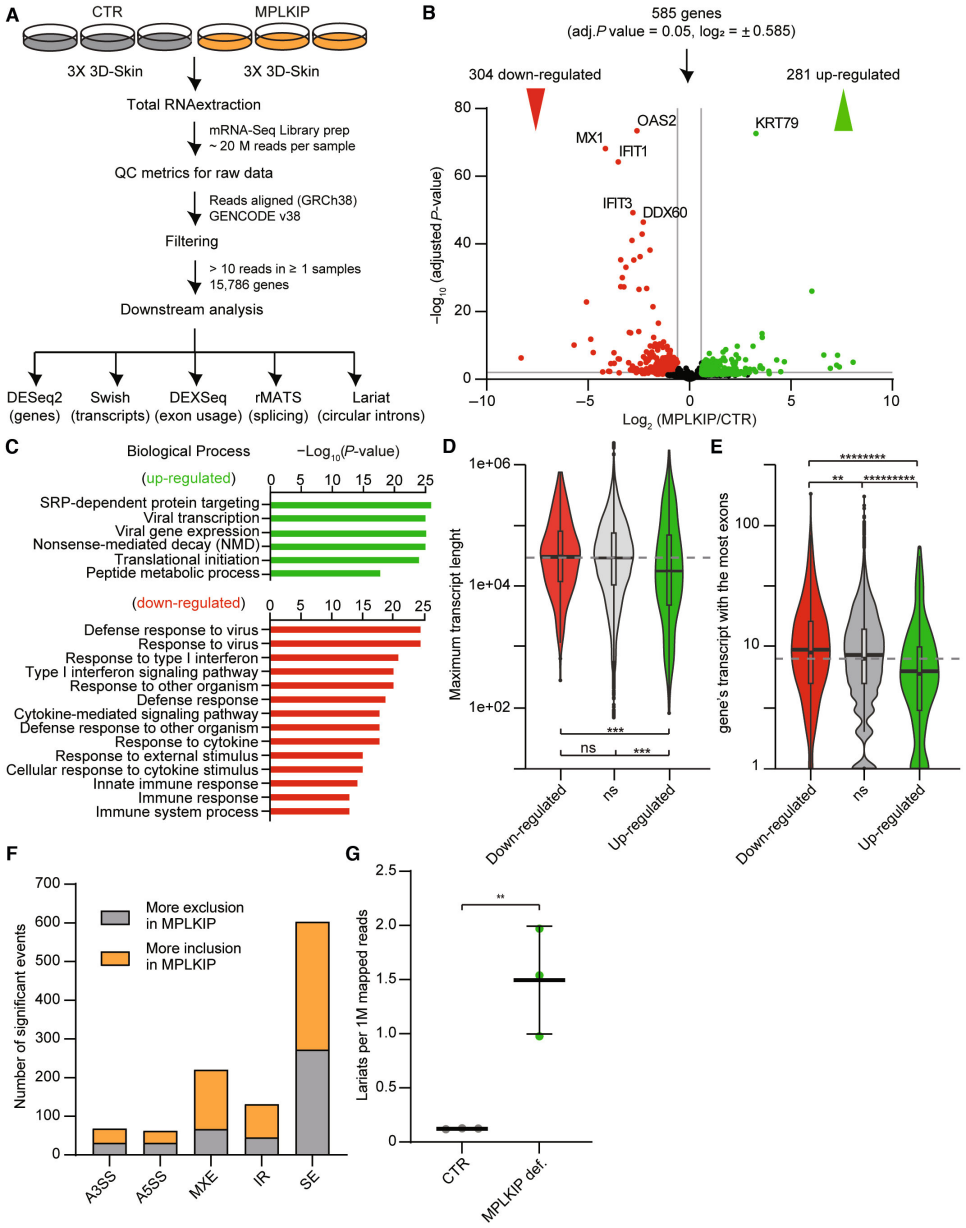
RNA‐seq of *MPLKIP*‐deficient TTD HSEs Schematic overview of the experimental setup for the RNA sequencing of HSEs and downstream analysis.Volcano plot of differentially expressed genes between 3 *MPLKIP*‐deficient HSEs (KI #7, KO #A10, and KO #16) and 3 control HSEs (CTR). Each red/green dot represents a significantly differentially down‐regulated or up‐regulated gene. Horizontal line: adjusted *P*‐value = 0.05. Vertical lines log_2_ Fold Change = ± log_2_(1.5).Top‐ranked up‐regulated (green bars) and down‐regulated (red bars) biological processes affected by *MPLKIP* loss in HSEs were determined by STRING analysis.Violin plots of the maximum transcript length in base pairs in all significantly down‐regulated (red), up‐regulated (green), and non‐significant (ns, grey) genes from B.Violin plots of the gene's transcript with the most exons in all significantly down‐regulated (red), up‐regulated (green), and non‐significant (ns, grey) genes from B.Number of significant altered splice events detected by rMATS. Alternative 3′ and 5′ Splice Sites (A3SS, A5SS), Mutually eXclusive Exons (MXE), Retained Introns (RI), and Skipped Exons (SE).Number of lariat per million reads detected in *MPLKIP*‐deficient HSEs (KI #7, KO #A10, and KO #16) and 3 control HSEs (CTR). Data information: (D, E). The boxplot boxes and mark denote the first, second, and third quartiles. The boxplot whiskers extend no further than 1.5 * the group's inter‐quartile range. The grey dashed line denotes the median of the non‐significant group. The dark lines in the violin plots denote the median of the kernel density estimates for each group. The statistical significance of the differences in transcript length and number of exons was obtained with a two‐sided Mann–Whitney test. ***P < 0.001; **P < 0.01; ********P < 0.00000001; *********P < 0.000000001; ns, not significant. (G). Data are represented as mean ± SD, unpaired *t*‐test. **P < 0.01. Source data are available online for this figure.

**Figure EV5:**
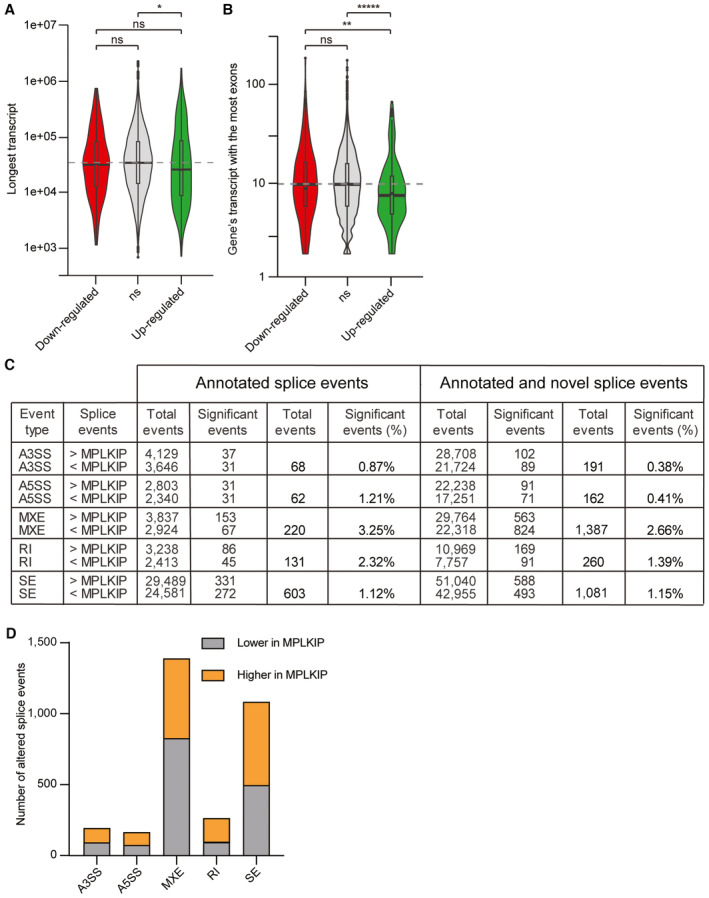
RNA‐seq analysis of *MPLKIP*‐deficient TTD HSEs A, BDifferential gene expression when limiting the analysis only to protein‐coding genes with at least two exons. Violin plots of the gene's longest transcript in bp (A) the gene's transcript with the most exons (B) in all significantly down‐regulated (red), up‐regulated (green), and non‐significant (ns, grey) genes (*n* = 3 biological replicates).CNumber of significant altered splice events detected by rMATS across all gene types when exploring novel un‐annotated splicing sites. Alternative 3′ and 5′ Splice Sites (A3SS, A5SS), Mutually eXclusive Exons (MXE), Retained Introns (RI), and Skipped Exons (SE).DOverview of identified altered splicing events by rMATS between *MPLKIP*‐deficient samples (MPLKIP) and control, based on annotated splicing sites (novel Splicing Sites = no) or also including novel splicing sites (novel Splicing Sites = yes). This analysis includes alternative 3′ splice sites (A3SSs), alternative 5′ splice sites (A5SSs), mutually exclusive exons (MXEs), intron retentions (RI), and skipped exons (SEs). Data information: (A, B). The boxplot boxes and mark denote the first, second, and third quartiles. The boxplot whiskers extend no further than 1.5 * the group's inter‐quartile range. The grey dashed line denotes the median of the non‐significant group. The dark lines in the violin plots denote the median of the kernel density estimates for each group. The statistical significance of the differences in transcript length and number of exons was obtained with a two‐sided Mann–Whitney test. *P < 0.05; **P < 0.01; *****P < 0.00001; ns, not significant. Source data are available online for this figure.

To understand the impact of *MPLKIP* deficiency on gene expression and pre‐mRNA splicing in HSEs, we performed mRNA sequencing and downstream analysis on three *MPLKIP*‐deficient (MPLKIP) and three control (CTR) normal HSEs (Fig 8A). We performed differential gene expression analysis between *MPLKIP*‐deficient samples and controls with DESeq2 (Love *et al*, 2014). We found 281 significantly up‐regulated and 304 down‐regulated genes in *MPLKIP*‐deficient samples compared to normal control samples, indicative for a subtly altered gene expression (Fig 8B). Many of the dysregulated genes are involved in epidermal development, including keratins (e.g., *KRT79*), collagenases (e.g., *COL11A1*), and specific markers of differentiation (e.g., *TGM1*, *SPRR2G*), which is consistent with the HSE phenotype. We performed a Gene Ontology (GO) term enrichment analysis on the differentially expressed genes and, interestingly, found the majority of significant up‐regulated genes in *MPLKIP*‐deficient samples being associated with SRP‐dependent protein targeting and nonsense‐mediated mRNA decay (NMD; Fig 8C). Moreover, we identified a large group of down‐regulated genes involved in the immune response (e.g. “response to virus”, “type I interferon signalling pathway”), including genes such as *DDX60*, *IFIT1*, *IFIT3*, *OAS2*, and *MX1* (Fig 8C). We also observed that down‐regulated genes in *MPLKIP*‐deficient samples have a higher number of exons, whereas up‐regulated genes were shorter and had less exons than both the down‐regulated and the non‐significant genes (Fig 8D and E). These results suggest that a specific splicing defect may be responsible for the observed transcriptome changes, diminishing our ability to detect longer genes and genes with more exons. When we filtered the dataset to perform the analysis only on protein‐coding genes with at least two exons, we observed the same tendency (Fig EV5A and B). We also analysed differential transcript expression with Kallisto (14) and Swish (15) (see Materials and Methods) and obtained results consistent with the differential gene expression analysis.

**Figure EV6:**
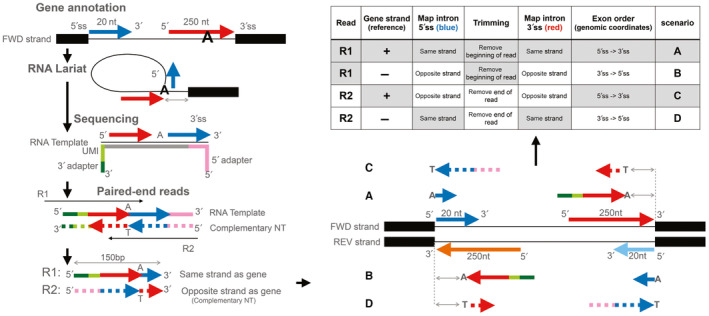
Schematic of the lariat detection process Schematic of the lariat detection process. Lariat loops originate during splicing from the intronic regions adjacent to the 5′ (blue) and 3′ (red) splicing sites. The spliced intron creates a loop at a branchpoint location (chiefly an adenine). Lariats can be identified by RNAseq when sequencing reads overlap this branching point. This creates a read hybrid between the intronic region adjacent to the 3′ splicing site upstream and the one adjacent to the 5′ splicing site. Paired‐end sequencing might give rise to 150 bp‐long reads overlapping the branchpoint region that are in the complementary strand (dashed arrows) and will map to the opposite strand in the genome. The combination of the gene's annotated strand and the two read pairs gives rise to four different scenarios to computationally map and identify reads originating from a lariat.Source data are available online for this figure.

We next analysed differential exon expression/usage using DEXSeq (Anders *et al*, 2012; Reyes *et al*, 2013; see Materials and Methods). This analysis revealed that 863 exons (0.346% of the total) were significantly differentially expressed in *MPLKIP*‐deficient samples. This result is consistent with the notion that dysregulated gene expression is observed in a more restricted set of genes/transcripts during skin differentiation (Mulder *et al*, 2012; Tanis *et al*, 2018). To detect differential alternative splicing events, we used rMATS (Multivariate Analysis of Transcript Splicing) to detect differential alternative splicing events (see Materials and Methods). This software uses an isoform annotation database to detect alternative (or aberrant) splicing events included differently between conditions. Our analysis revealed a number of splicing events whose inclusion differed significantly between *MPLKIP*‐deficient samples and controls. Specifically, 68 (0.87% significant events among all the detected events of this type) alternative 3′ splice sites (A3SSs), 62 (1.21%) alternative 5′ splice sites (A5SSs), 220 (3.25%) mutually exclusive exons (MXEs), 131 (2.32%) intron retentions (RI), and 603 (1.12%) skipped exons (SEs) (Figs 8F and EV5C). Furthermore, we ran an additional analysis to include splicing events involving novel or un‐annotated splicing sites, which showed an even higher number of significant aberrant splicing events detected (Fig EV5C and D). Finally, we used the RNA sequencing data to quantitatively assess the number of lariat sequences (Fig EV6). On average, we detected 263 lariat reads in the *MPLKIP*‐deficient samples (1.49 per million reads) compared to 25 reads in the control samples (0.12 per million reads), indicating an 11.8‐fold enrichment of lariats in the *MPLKIP*‐deficient samples compared to controls (Fig 8G). These results indicate that lariat processing is affected in *MPLKIP*‐deficient cells.

### Impaired protein expression in *MPLKIP* ‐deficient TTD HSEs

**Figure 9:**
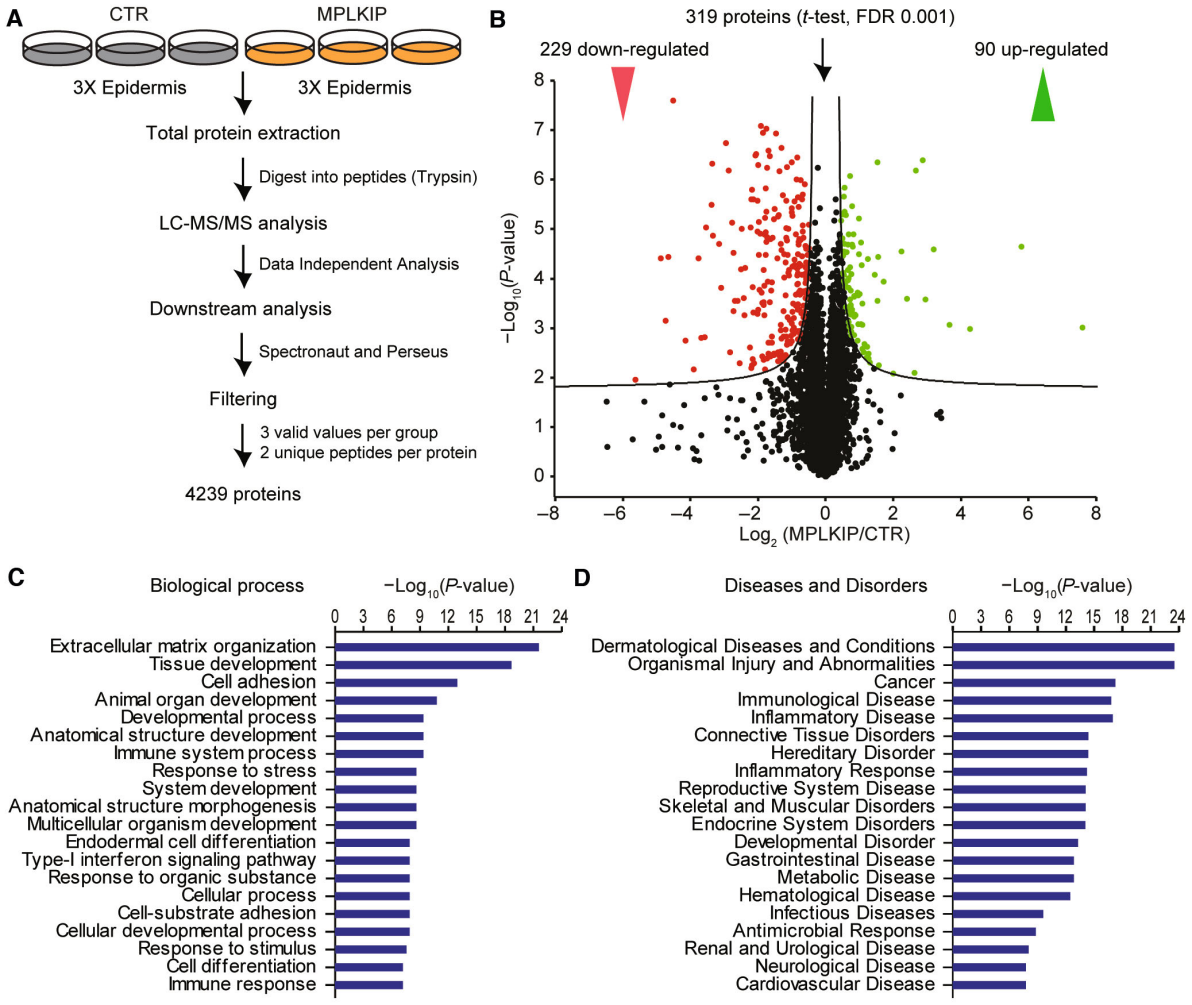
*MPLKIP*‐deficient TTD epidermis have imbalanced protein homeostasis Schematic overview of the experimental setup of label‐free quantitative proteomics and downstream analysis.Volcano plot displaying differentially expressed proteins, with log_2_ fold enrichment of MPLKIP‐associated proteins over control proteins plotted against their significance (*P*‐value, −Log_10_). Each red or green dot represents a significantly differentially down‐regulated or up‐regulated protein, respectively.Top‐ranked biological processes affected by MPLKIP loss in the epidermis are determined by ingenuity pathway analysis (IPA).Top‐ranked diseases and disorders affected by MPLKIP loss in the epidermis are determined by ingenuity pathway analysis (IPA). Source data are available online for this figure.

To gain insight into the functional relevance of the observed altered transcriptional profile by MPLKIP deficiency, we performed label‐free quantitative MS. We dissected the epidermis of *MPLKIP*‐deficient HSEs and prepared protein lysates from three biological replicates of *MPLKIP*‐deficient and normal control epidermis that were analysed by liquid chromatography‐tandem mass spectrometry (LC‐MS/MS) (Washburn *et al*, 2001; Fig 9A). We found in total 319 significant differentially expressed proteins, with 90 up‐regulated and 229 down‐regulated proteins in *MPLKIP*‐deficient epidermis compared to normal control epidermis (Fig 9B). Among the top‐ranked affected biological processes, we identified 47 proteins involved in extracellular matrix organization, including significant dysregulation of collagens such as COL17A1 and COL7A1. Furthermore, 88 dysregulated proteins were involved in tissue development, including keratins KRT4, KRT13, and KRT79, and proteins of the SPRR family like SPRR2G. These data show a clearly imbalanced tissue‐specific proteome profile in *MPLKIP*‐deficient epidermis, which is likely caused by the above‐described transcriptional and differentiation disturbances. Using biological pathway analysis, we also observed a large number of proteins associated with immunological pathways, including immune system processes and the type‐I interferon signalling pathway, in line with the transcriptome analysis (Fig 9C). Most of these proteins were down‐regulated, including DDX58, DDX60, and MX1. We also investigated disease associations and found that the top‐ranked diseases and disorders were predominantly involved in dermatological diseases (250 proteins), organismal injury and abnormalities (254 proteins), immunological disease (164 proteins), inflammatory disease (109 proteins), and connective tissue disorders (96 proteins) (Fig 9D).

## Discussion

We analysed a cohort of seven NPS‐TTD subjects from five non‐related families in Türkiye (Fig 1) and identified three biallelic truncating mutations in the *MPLKIP* gene. These include homozygous nonsense NM_138701.4(MPLKIP):c.85G>T (p.Gly29Ter) in three unrelated families (patients TTD287IS, TTD288IS, TTD303IS, and TTD295IS), homozygous frameshift c.505dup (p.Thr169AsnfsTer32) in another family (patient TTD299IS), and novel homozygous frameshift c.61del (p.Trp21GlyfsTer132) in one family (TTD289IS and TTD290IS). All individuals in this cohort displayed sparse and brittle hair with trichorrhexis nodosa and tiger‐tail banding pattern, keratosis pilaris, dry skin, dysplastic nails, hypogonadism, osteoporosis, microcephaly, and intellectual deficit, indicating these as the most consistent clinical findings of MPLKIP‐associated NPS‐TTD (Fig 2; Table EV1 and Materials and Methods). Central osteosclerosis, a common finding in TTD patients, was ruled out in all our patients (DiGiovanna *et al*, 2022). The bifid uvula and single central incisor were detected in one patient each, representing midline anomalies not reported before in the NPS‐TTD spectrum. However, we have not ruled out the presence of a concomitant genetic disorder that could possibly be attributed to the atypical midline findings in our patients. Thus, further patient reports are required to include these findings as a part of the TTD phenotype.

We previously proposed (Theil *et al*, 2019) that most of the key TTD features of brittle hair, nails, and scaly skin are a consequence of protein instability of factors involved in different steps of gene expression in either transcription (mutated *ERCC2*, *ERCC3*, *GTF2H5*, or *GTF2E2*), splicing (mutated *RNF113A*), or translation (mutated *AARS1*, *CARS1*, *MARS1*, or *TARS1*). This highlights a basic concept on how the fragility of vital gene expression factors leads to a surprising set of common clinical features. However, such a link with gene expression was thus far not identified for MPLKIP. Here, we showed that MPLKIP associates with pre‐mRNA processing factors, thus also linking this TTD‐associated factor to gene expression (Fig 3). This analysis further confirmed the association with the key cell‐cycle‐regulating protein PLK1. Previous observations suggested that MPLKIP is important for the maintenance of cell cycle integrity since overexpression or depletion of the protein results in deregulation of the cell cycle (Zhang *et al*, 2007). However, we did not observe any cell cycle or proliferation defects in *MPLKIP* patient‐derived primary fibroblasts or in *MPLKIP*‐deficient cell lines that we generated in this study. This apparent discrepancy may be related to the use of different cell types. Since we did not observe aberrant proliferation neither in the reconstituted HSE model generated from *MPLKIP*‐deficient fibroblasts nor in keratinocytes, we believe that possible cell cycle defects are not the root‐cause of the TTD‐specific ectodermal phenotypes.

MS analysis revealed the strongest interaction with: (i) the debranching complex (DBR1 and CWF19L1), and with (ii) the NTC (XAB2, AQR, PPIE, and ISY1), which was also recently found by others (Townley *et al*, 2023). DBR1 is an RNA lariat‐debranching enzyme that linearizes the splicing‐generated intron lariats at the 2′‐5′phosphodiester linkage at the intronic branch point when released from post‐splicing complexes by the DEAH‐box ATPases (Chapman & Boeke, 1991; Montemayor *et al*, 2014; Mohanta & Chakrabarti, 2021). Debranching further provides the coordinated release of splicing factors from post‐splicing complexes (Martin *et al*, 2002; Tanaka *et al*, 2007; Fourmann *et al*, 2013), such as the U2, U5, U6 snRNPs, and involves the handover of excised lariats from the NTC to the debranching complex (Yoshimoto *et al*, 2009; Fourmann *et al*, 2013). Proper lariat turnover is therefore important for efficient recycling of splicing proteins and the availability of ribonucleotides, impairment of which affects both splicing and transcription (Mohanta & Chakrabarti, 2021). Here, we demonstrate that MPLKIP is important for DBR1 stability. The cellular amount of this specific protein was severely reduced in both *MPLKIP*‐deficient primary fibroblasts (Fig 4) and *MPLKIP*‐deficient N/TERT keratinocytes (Fig 6). Conversely, MPLKIP protein levels were severely reduced upon siRNA‐mediated DBR1 depletion in primary fibroblasts, arguing for a mutual dependency for complex stability. This observation is consistent with previous work on TTD, in which an alteration in one subunit makes the entire protein complex, such as TFIIE and TFIIH unstable (Vermeulen *et al*, 2000; Botta *et al*, 2002; Kuschal *et al*, 2016; Theil *et al*, 2019). We further showed that MPLKIP is most likely required for bridging the NTC complex to DBR1 (Fig 5C).

Although no clear functional domain could be identified in the MPLKIP amino acid sequence, the protein is predicted to have intrinsically disordered regions (IDRs) at its N‐terminal that is enriched in charged and structure‐breaking residues (e.g., glycine and proline) and contains characteristic prion‐like domains. Perhaps this highly flexible and disordered N‐terminal part of MPLKIP is important to promote DBR1 debranching activity, which could be functionally regulated by post‐translational modifications (PTMs; Townley *et al*, 2023). In fact, IDRs are more prone to be modified by PTMs (Iakoucheva *et al*, 2004; Hansen *et al*, 2006), and MPLKIP was previously shown to be one of the most heavily methylated proteins in the cell (Larsen *et al*, 2016). It is therefore tempting to speculate that MPLKIP has two distinct functions: (i) stabilizing DBR1 cellular protein content through its physical interaction and (ii) stimulating DBR1 activity by coordinated binding to spliced lariat‐introns. This scaffolding role for DBR1 could be achieved by its potential to phase separate, *that is*, a common feature driven by IDRs (Posey *et al*, 2018; Martin & Holehouse, 2020) that promotes interactions with the NTC splicing complex, which would however require further investigation. DBR1 is a rate‐limiting enzyme that is crucial for RNA homeostasis in all organisms, but is especially vital in higher eukaryotes where the number and turnover of intron‐containing genes is high (Chapman & Boeke, 1991; Lynch & Richardson, 2002; Zheng *et al*, 2020).

It is thus expected that impaired DBR1 protein levels and function by MPLKIP‐deficiency will cause aberrant splicing and transcriptome changes and, consequently, in proteome content. The signature TTD features are mainly expressed in epidermal tissue, of which differentiation is controlled by a complex interplay between regulated transcription, epigenetics, and stromal‐derived cues (*i.e*., dermal and extracellular matrix [ECM]). We therefore investigated possible splicing and transcription changes in a three‐dimensional human skin equivalent that mimics TTD (Fig 7). *MPLKIP*‐deficient HSEs display impaired epithelial barrier formation with a decreased epidermal thickness and reduced number of corneocyte layers in the stratum corneum. To further investigate at the molecular level the changes in epidermal morphogenesis, we employed a combination of mRNA sequencing and mass spectrometry to examine changes in gene expression and protein abundance during the differentiation of keratinocytes.

Our analysis of the mRNA sequencing data revealed alterations in gene expression in *MPLKIP*‐deficient samples compared to the control samples (Fig 8A and B). We observed that down‐regulated genes generally had a higher number of exons, whereas up‐regulated genes had a lower number of exons and are skewed towards shorter transcripts (Figs 8D and E, and EV5A and B). Additionally, we identified an increased number of differentially expressed exons and alternative splicing events, including alternative 3′/5′splice sites (A3SSs and A5SSs), mutually exclusive exons (MXEs), retained introns (RIs), and skipped exons (SEs) in *MPLKIP*‐deficient samples (Figs 8F and EV5C and D). These differences were consistent with previous observations when core splicing factors were depleted (Tanis *et al*, 2018). Moreover, our investigation suggests that reduced DBR1 protein levels/activity in *MPLKIP* deficiency may hinder the proper processing of intron RNA lariats, resulting in their accumulation. Although no significant difference was found in intronic reads between *MPLKIP*‐deficient samples and controls, the increase in RNA lariat abundance in *MPLKIP*‐deficient samples (Fig 8G) supports previous observations in *DBR1*‐deficient cells (Salem *et al*, 2003; Li *et al*, 2016; Han *et al*, 2017; Zhang *et al*, 2018; Wan *et al*, 2021). It is worth noting that altering the levels of different splicing factors, as observed here for DBR1, the ratio of specific splicing events can be changed. For example, serine‐arginine (SR) proteins and heterogeneous nuclear ribonucleoproteins (hnRNPs) can antagonize each other in a concentration‐dependent manner. Altered splicing ratios will eventually result in the production of aberrant proteins that will jeopardize cellular homeostasis (Okunola & Krainer, 2009). Interestingly, we found that nonsense‐mediated mRNA decay (NMD) and SRP‐dependent protein targeting are both up‐regulated in *MPLKIP*‐deficient HSEs, most likely as a consequence of aberrant splicing (Wong *et al*, 2013). The upregulation of these pathways might suggest that cells are attempting to prevent the accumulation of improperly spliced RNA and its consequent abnormal or truncated proteins that could negatively impact cellular function, which demonstrates the crucial role of DBR1 in maintaining RNA and proteome homeostasis in higher eukaryotes. The predominance of ribosomal genes rather than core NMD/SRP‐associated genes in the STRING analysis raises the question of why specifically ribosomal proteins are upregulated. The efficiency of splicing varies between RPGs, and this variation helps adjust the expression levels of each RPG and determines the expression ratio of different RPGs. This splicing‐dependent regulation of RPGs is not static but responds dynamically to stress, allowing for adaptive adjustments in RPG expression ratios (Parenteau *et al*, 2011; Petibon *et al*, 2016, 2021; Ghulam *et al*, 2020). Possible explanations for this up‐regulation might be to increasing the availability of ribosomal components, enhancing the cell's capacity to detect and eliminate improperly spliced RNA, thereby preventing the generation of abnormal or truncated proteins. This would require further investigation.

Biallelic mutations in *DBR1* were previously found to be associated with aberrant splicing defects, RNA lariat accumulation, and susceptibility to viral infections in the brainstem (Zhang *et al*, 2018). Interestingly, we also found associations with impaired immunity in *MPLKIP*‐deficient samples, in which DBR1 is indirectly impaired. Biological pathway analysis revealed that the most significant group of down‐regulated genes in *MPLKIP*‐deficient HSE was associated with cellular immunity. It is striking to note that many TTD individuals die at a relatively young age due to recurrent infections. A history of frequent sinopulmonary infections and selectively high levels of serum IgA were observed in the majority of the patients in our cohort. High levels of serum IgA may indicate chronic infection as well as a primary immune deficiency or autoinflammatory disease. Down‐regulation of the immune response at the transcript level was recapitulated within the proteomic analysis. Several proteins within this pathway, including important RNA sensors such as DDX58, were down‐regulated in *MPLKIP*‐deficient HSEs. DDX58 activates a cascade of transcription factors that induce pro‐inflammatory cytokines and IFN‐I stimulated genes, such as *STAT1*, *STAT2*, *MX1*, *OAS2*, and *DDX60* (Honda *et al*, 2006; Sadler & Williams, 2008; Verhelst *et al*, 2012; Schneider *et al*, 2014). Pathogenic variants in *DDX58* have been associated with Singleton‐Merten syndrome 2 and many of the reported patients exhibit skin inflammation such as psoriasiform rash (Jang *et al*, 2015; Prasov *et al*, 2022). Differential expression of interleukins was, however, not observed in our MS, likely because of their general low abundance in the absence of inflammation. Epithelial dysfunction, as in TTD, is marked by changes in processes that regulate tissue homeostasis and repair, leading to a transition from a stable to a remodelling state. This state is characterized by alterations in cell proliferation, differentiation, and death, resulting in reduced differentiation signals and the accumulation of immature or abnormal cells. These changes contribute to the development of various epithelial disorders. Additionally, remodelling is associated with a downregulation of the innate immune system, which impairs the ability of epithelial tissues to respond to stressors and pathogens, further exacerbating tissue damage and contributing to the progression of epithelial dysfunction (Haensel *et al*, 2020). It would thus be interesting to monitor the expression of interleukins during an inflammatory response in the HSE models, in which both dendritic cells and macrophages should be included.

Proteome analysis further identified significant down regulation of a large group of keratinocyte‐specific structural and ECM‐associated key regulatory proteins, including collagens, keratins, desmocollins, transglutaminases, and members of the small proline‐rich protein (SPRR) family. Inherited mutations in most of the genes that encode for these dysregulated structural and ECM proteins are associated with severe skin pathology. For instance, collagens support skin and other tissues via the extracellular matrix (ECM) (Shoulders & Raines, 2009), and gene mutations can cause various cutaneous disorders. *COL7A1* mutations lead to epidermolysis bullosa (Varki *et al*, 2007), and *COL17A1* mutations cause bullous pemphigoid (McGrath *et al*, 1995). Additionally, keratins are vital for epidermal morphology, and mutations in these genes cause skin and hair disorders (Ho *et al*, 2022). For example, *KRT4* and *KRT13* mutations cause white sponge nevus with spongy patches on mucous membranes (Richard *et al*, 1995; Rugg *et al*, 1995). KRT79 protein is highly expressed in hair follicles and nails, and may regulate hair formation and maintenance (Veniaminova *et al*, 2013). Differential KRT79 expression may therefore contribute to the brittle hair phenotype. Moreover, desmocollins are essential structural components of desmosomes, maintaining tissue integrity. Mutations in *DSC2* cause palmoplantar keratoderma and woolly hair syndrome, characterized by thickened skin on the feet and hands and curly, brittle hair due to epidermal differentiation and cornification disorders (Simpson *et al*, 2009). It is worth noting that there is no full overlap between altered mRNA expression and protein abundance, making them more complementary rather than confirmatory or correlative. We also do not observe a clear correlation between the effects of gene length on alteration in RNA expression and changes in protein abundance. It is however known that in general terms, there is only a poor correlation (30–40%) between mRNA expression levels and protein abundance (Maier *et al*, 2009; Vogel & Marcotte, 2012). mRNA sequencing provides solely information about mRNA expression and lacks the dynamic nature of protein regulation, which is affected by post‐translational modifications, translation efficiency, protein degradation, and turnover rates, which adds further to the complex relationship between RNA and protein levels.

The human small proline‐rich protein (SPRR) family functions as crosslinking proteins that form bridges between other proteins that comprise the cornified cell envelope and thereby contribute to the skin host defence against systemic infections (Elder & Zhao, 2002; Lin *et al*, 2003; Zhang *et al*, 2022). For instance, uncontrolled expression of *SPRR2* genes has been linked to impaired responses to stress, affecting cell migration and wound healing (Lin *et al*, 2003). Interestingly, *SPRR2* expression was also severely reduced in the skin of the *ERCC2*‐mutated TTD mouse model (De Boer *et al*, 1998). It thus seems that dysregulated SPRR protein family expression observed in *MPLKIP*‐deficient HSEs, represents a common phenomenon linked to TTD pathology.

In summary, we have obtained important mechanistic insight into the biological function of MPLKIP, which has been a mystery since its discovery. MPLKIP is important for stabilizing DBR1 steady‐state protein levels and subsequent mRNA maturation. MPLKIP loss and associated DBR1 fragility cause impaired splicing and gene expression in highly differentiated epithelial 3D skin equivalents, most likely due to defects in primary transcript maturation. Epithelial barrier function seems to be severely “leaky” in *MPLKIP*‐deficient HSEs and many developmental processes are impaired. Interestingly, several pathways linked to the immune system were severely down‐regulated in these “leaky” epithelia, which may provide reliable targets for the development of diagnostics and therapeutics for pathological conditions linked to TTD, which is highly relevant for the pathogenesis of recurrent infections in TTD.

During the reviewing process of this manuscript, another paper was published describing some similar findings of MPLKIP function in RNA processing (splicing) and lariat turnover (Townley *et al*, 2023).

## Materials and Methods

### Clinical data

The subjects were recruited in the outpatient clinics of the Medical Genetics Department of Istanbul Medical Faculty. Informed consent was obtained from all human subjects, and the experiments conformed to the principles set out in the WMA Declaration of Helsinki and the Department of Health and Human Services Belmont Report. The legal representatives of the subjects were informed, and signed consent was obtained for genetic analyses, research, and the publication of clinical data and photographs. Medical and family history data was gathered for each family. Radiological and laboratory investigations including brain MRIs, electroencephalography, Denver II or WISC‐R, metabolic screening, hemogram, haemoglobin electrophoresis, sex hormones profile, bone mineral density, pelvic and/or scrotal ultrasonography, audiometric assessment, and routine ophthalmological examination were performed. All subjects were consulted by paediatric endocrinologist, a paediatric neurologist, and a dermatologist for long‐term follow‐up.

#### Subject TTD295IS

This boy (Fig 1A and F), firstborn to first‐degree cousins, was referred to the genetics outpatient clinics due to global developmental delay and abnormal hair texture. He was born at term following an uneventful pregnancy, with a weight of 3,500 g (0.22 SD). He stayed 15 days in the neonatal intensive care unit due to respiratory and feeding difficulties, which ameliorated after 4 months. The workup comprising cranial MRI, routine biochemistry, a complete blood count, thyroid function tests, and extensive metabolic screening, was unremarkable. He achieved head control at 9 months, sat without support at 18 months, walked at 3.5 years, spoke single words at 3 years, and was able to use three‐word sentences at 6 years of age.

Physical examination at the age of 2 years and 9 months showed a weight of 13.8 kg (−0.29 SD), height of 87 cm (−2.01 SD), and OFC of 44 cm (−3.81 SD). He had micro brachycephaly, hyperkeratotic plaques on the scalp, kinky, sparse, dry, and brittle hair and eyebrows, malar hypoplasia, strabismus, infraorbital creases with thin skin, a prominent nasal root, a narrow nasal tip, a low hanging columella, a short philtrum, a prominent nasolabial sulcus, saggy cheeks, a high arched palate, enamel hypoplasia, prominent ears, bilateral clinodactyly, and nail dystrophy. Skin was dry with ichthyosiform areas on the sun‐exposed areas, which was mistaken for photosensitivity at first. He displayed prominent truncal hypotonia. Hair examination revealed trichorrexis nodosa, trichoschisis, and a typical “tiger‐tail” banding pattern under a polarized light microscope (Fig 2A and B). Sanger sequencing of the *MPLKIP* gene identified a homozygous nonsense variant in *MPLKIP* [NM_138701.4:c.85G>T, p.(Gly29Ter)] (Fig 2C).

On the last follow‐up visit at age 14 years and 6 months, weight was 30.8 kg (−3.71 SD), height was 136 cm (−4.3 SD), and OFC was 46.5 cm (−6.47 SD). He had been operated on due to bilateral cryptorchidism at 8 years of age. Ichthyosiform changes on the sun‐exposed areas ameliorated over time, ruling out photosensitivity. In addition to his previously recorded findings, he displayed pseudo‐clubbing of nails, anhidrosis, keratosis pilaris on the extensor surfaces of extremities, cubitus valgus, pes planus, micropenis, and bilateral small testes with a volume of 4 cc each. The systemic and neurological examinations were otherwise unremarkable. The hormone profile revealed prepubertal gonadotropin levels with FSH level of 1.11 mlU/ml (normal: 1.5–12.4 mlU/ml), LH level of 1.01 lU/ml (normal: 1.7–8.6 lU/ml), and a testosterone level of 1.22 ng/ml (normal: 0.07–8 ng/ml). These results were compatible with delayed puberty. Lumbar bone mineral density showed osteoporosis (*Z* score: −3.2). Expressive speech and articulation were poor. His family reported recurrent middle ear infections, chronic constipation, functional daytime urinary incontinence, recurrent sinopulmonary and urinary infections, and persistent blepharitis. He showed moderate intellectual impairment and behaviour abnormalities including hyperactivity, poor eye contact, and aggressive behaviour. Additional features were obsessive‐compulsive behaviour, sleeping disturbances, and hyperacusis.

#### Subject TTD287IS

This boy (Fig 1B and F), was born at term, to parents originating from the same village. Pregnancy was complicated with oligohydramnios at the last trimester. Birth weight was 2,800 g (−1.34 SD). He achieved head control at 6 months, sat without support at 8 months, crawled at 14 months, walked at 2 years and 6 months, used single words at 8 years. He was referred to the genetics outpatient clinics due to psychomotor retardation and facial dysmorphic findings.

Physical examination at the age of 15 months showed a weight of 8,600 g [−1.90 SD], height of 75 cm [−1.54 SD], and an OFC of 46 cm [−1.21 SD]. He had sparse, woolly, hypopigmented, and brittle hair and eyebrows, left esotropia, down‐slanted palpebral fissures, malar hypoplasia, infraorbital creases with thin skin, anteverted nares, thick nasal alae, low hanging columella, short philtrum, prominent central incisors, saggy cheeks, retrognathia, prominent ears, dry skin, macular eczematous lesions on the external surfaces of extremities, and nail dystrophy. The systemic and neurological examination were otherwise unremarkable. Hair examination revealed trichorrexis nodosa, trichoschisis, pili torti, and a typical “tiger‐tail” banding pattern under polarized light microscope (Fig 2A and B). Sanger sequencing of the *MPLKIP* gene identified the a homozygous nonsense variant in *MPLKIP* [NM_138701.4:c.85G>T, p.(Gly29Ter)] (Fig 2C).

At last examination at the age of 15 years and 4 months, weight was 39 kg [−3.02 SD], height was 152 cm [−2.75 SD], and OFC was 49 cm [−5.17 SD]. Penis length was normal and testes were palpable in the scrotum with volumes of 8 cc each. He had hypergonadotropic hypogonadism, with FSH level of 21.7 mIU/ml (normal: 1.5–12.4 mIU/ml), LH level was 3.28 lU/ml (normal: 1.7–8.6 mIU/ml), and testosterone level was 0.02 ng/ml (normal: 0.07–8 ng/ml). Lumbar bone mineral density showed osteoporosis (*Z* score: −3.1). He experienced recurrent sinopulmonary infections, and poor expressive speech. Cranial MRI showed hypoplasia of corpus callosum. He had moderate intellectual impairment, pronounced expressive speech delay, and behaviour abnormalities including aloofness, stubbornness, and aggressive behaviour.

#### Subject TTD288IS

This girl (Fig 1C) is the second child of first‐degree cousins and was referred to the genetics outpatient clinics due to growth retardation, microcephaly, and abnormal hair texture at the age of 2 years. She has two similarly affected first‐degree cousins (Fig 1C and F). She was born at term with a weight of 2,800 g (−1.20 SD), length of 49 cm (−0.19 SD), and a head circumference of 34 cm (−0.36 SD). Extensive metabolic screening showed normal results. She had been hospitalized four times in the first 2 years of life due to recurrent bronchiolitis. She achieved head control at 6 months, sat without support at 12 months, walked at 2 years, spoke single words at 4 years, and built two‐word sentences at 10 years of age. Cranial MRI at the age of 15 months showed delayed myelination prominent on the frontotemporal region. An electroencephalogram was performed to exclude epileptic encephalopathies and epileptiform changes without clinical seizures. She has not had any seizures ever since.

Physical examination at the age of 2 years showed a weight of 9.5 kg (−1.85 SD), a height of 90 cm (0.90 SD), and an OFC of 42 cm (−4.2 SD). She had microcephaly, sparse, dry, and brittle hair and eyebrows; bilateral epicanthal folds; blue sclerae; anteverted nares; thick nasal alae a low hanging columella; short and smooth philtrum; prominent central incisors; high‐arched palate; retrognathia; attached ear lobes; hyperextensibility of elbows; bilateral clinodactyly of the fifth fingers and bilateral pes planus. Hair examination revealed trichorrhexis nodosa and a typical “tiger‐tail” banding pattern under a polarized light microscope (Fig 2A and B). Sanger sequencing of the *MPLKIP* gene identified a homozygous nonsense variant [NM_138701.4:c.85G>T, p.(Gly29Ter)] (Fig 2C).

On the last follow‐up visit at age 13 years and 7 months, weight was 54 kg (0.28 SD), height was 142 cm (−2.9 SD), and OFC was 50 cm (−3.72 SD). In addition to her previously recorded findings, she had capillary haemangiomata on the extensor surfaces of the right leg and arm. The gait was ataxic. She also had cold hands and feet at all times, indicative of vasomotor disturbance. The systemic and neurological examinations were otherwise unremarkable. She manifested symptoms of polyphagia and was clinically obese with a BMI of 26.8 (1.92 SD). Prader‐Willi syndrome was ruled out with methylation‐specific MLPA analysis. Lumbar bone mineral density showed osteoporosis (*Z* score: −3.6). She had delayed puberty with a FSH level of 6.1 mIU/ml [normal: 3.6–12.6 mIU/ml], LH level of 0.1 IU/ml [normal: 2.4–12.5 IU/ml], and an estradiol level of 5 pg/ml [normal: 10–100 pg/ml]. Follow‐up hormone studies at 14 years showed hypergonadotropic hypogonadism, with a FSH level of 39.85 mIU/ml (normal: 1.5–12.4 mIU/ml), LH level of 19.2 IU/ml (normal: 1.7–8.6 IU/ml), and an estradiol level of 5 pg/ml (normal: 10–100 pg/ml). She had moderate intellectual impairment, severe expressive speech delay, and behaviour abnormalities including obsessive‐compulsive features, excessive appetite, and obsession with eating. She was administered sertraline and carbamazepine as mood stabilizers by a child psychiatrist to control over‐activity at the age of eight, which partly ameliorated her findings. Carbamazepine was discontinued after 17 years of age, while sertraline is still in use.

#### Subject TTD303IS

This boy (Fig 1C; no photographs), the similarly affected first‐degree cousin of subject TTD288IS, was born at term after an uneventful pregnancy with a weight of 3,000 g (−0.89 SD). He was examined in our outpatient clinics at the age of 15 due to similar findings with his cousin. He gained head control at 3 months, sat without support at 8 months, walked at 4 years, and spoke single words at 6 years. He had poor expressive speech, and was diagnosed with hyperactivity‐attention deficit disorder with aggressive behaviour by a child psychiatrist and was treated by olanzapine.

Physical examination at the age of 17 years showed a weight of 38 kg (−4.55 SD), a height of 148 cm (−4.17 SD), and OFC of 48.5 cm (−6.2 SD). He had sparse, woolly, hypopigmented, and brittle hair and eyebrows; hyperpigmented, hyperkeratotic plaques on the scalp; prominent nasal root; prominent ears; bilateral clinodactyly of the fifth fingers; dry and loose skin; keratosis pilaris on extensor surfaces of extremities; and nail dystrophy. Penis length was normal, and testes were palpable in the scrotum with volumes of 4 cc each. The systemic and neurological examinations were otherwise unremarkable. Hair microscopy was not performed. Sanger sequencing of the *MPLKIP* gene identified the familial variant in homozygous form [NM_138701.4:c.85G>T, p.(Gly29Ter)]. Hormone work‐up at 17 years of age showed a FSH level of 1.4 mIU/ml (normal: 1.5–12.4), LH level of 1.6 IU/ml (normal: 1.7–8.6 IU/ml), and testosterone level of 1.36 ng/ml (normal: 0.07–8 ng/ml) compatible with delayed puberty.

#### Subject TTD289IS

This girl (Fig 1D and F), the second child of first‐degree cousins, was born at term following an uneventful pregnancy with a weight of 2,300 g (−2.36 SD) and a length of 52 cm (1.22 SD). She was referred to our outpatient clinics due to hair abnormalities and an intellectual deficit. She achieved head control at 2 months, unsupported sitting at 6 months, walking at 18 months, speaking single words at 18 months, two‐word sentences at 5 years, and three‐word sentences at 6 years of age. The family described hyperhidrosis with onset in early childhood.

Physical examination at 10 years and 10 months showed a weight of 25 kg (−2.02 SD), a height of 130 cm (−2.06 SD), and OFC of 49.5 cm (−2.85 SD). She had sparse, woolly, hypopigmented, and brittle hair and eyebrows; hyperpigmented, and hyperkeratotic plaques on the scalp; prominent nasal root; infraorbital creases with thin skin; malar hypoplasia; thick nasal alae; low hanging columella; short and smooth philtrum; thin upper lip vermilion; high‐arched palate; wide uvula; under folded helices with prominent antihelices; dry and loose skin; keratosis pilaris and nail dystrophy. She had horizontal nystagmus. The systemic and neurological examinations were otherwise unremarkable. Hair examination revealed trichorrhexis nodosa and a typical “tiger‐tail” banding pattern under a polarized light microscope (Fig 2A and B). Sanger sequencing of the *MPLKIP* gene identified a homozygous frameshift variant [NM_138701.4:c61delT, p.(Trp21GlyfsTer132)] (Fig 2C).

Cranial MRI at 11 years of age showed a dilated posterior fossa (with differential diagnoses of mega cisterna magna and arachnoid cyst) and periventricular hyperintense lesions on T2‐FLAIR images. Lumbar bone mineral density showed marked osteoporosis (*Z* score: −3.2, *T* score: −7.5) at the age of 11 years. 25‐OH vitamin D level was 13.3 ng/ml (< 20 ng/ml: deficiency), mild hypocalcaemia (Ca level of 8.9 mg/dl, normal: 9.2–11 mg/dl), and high levels of ALP (230 U/l, normal: 50–85 U/l), high phosphate levels (4.8 mg/dl, normal range: 2.7–4.5 mg/dl), indicative for vitamin D deficiency. Hormone work‐up showed a FSH level of 8.2 mIU/ml (normal: 3.6–12.6 mIU/ml), LH of 0.1 IU/ml (normal: 2.4–12.5 IU/ml) and an estradiol of 5 pg/ml (normal: 10–100 pg/ml). With these results, vitamin D replacement treatment was initiated, and she was administered estradiol patches. Further work‐up comprising a complete blood count, haemoglobin electrophoresis, thyroid function tests, echocardiography, abdominopelvic ultrasound, and audiogram showed unremarkable results.

On the last follow‐up visit at age 17 years and 7 months, weight was 40 kg (−3.3 SD), height was 152 cm (−1.9 SD), OFC was 51 cm (−4.0 SD). In addition to her previously recorded findings, she had kyphoscoliosis. Follow‐up hormone studies at that age showed hypergonadotropic hypogonadism, with a FSH level of 28.8 mIU/ml (normal: 1.5–12.4 mIU/ml), LH level of 17.4 IU/ml (normal: 1.7–8.6 IU/ml), and an estradiol of 91 pg/ml (normal: 10–100 pg/ml) under estradiol patch treatment.

#### Subject TTD290IS

This girl (Fig 1D and F), the similarly affected sister of subject TTD289IS, was born at term after an uneventful pregnancy with a weight of 2,200 g (−2.7 SD), length of 52 cm (1.22 SD), and head circumference of 33 cm (−1.09 SD). She was referred to our outpatient clinics due to having similar findings with her sister. She achieved head control at 3 months, unsupported sitting at 7 months, walking at 24 months, speaking single words at 24 months, two‐word sentences at 5 years, and three‐word sentences at 6 years of age.

Physical examination at 13 years and 3 months showed a weight of 28 kg (−4.9 SD), height of 133.5 cm (−4.17 SD), and OFC of 50 cm (−3.56 SD). She had sparse, woolly, hypopigmented; and brittle hair and eyebrows; hyperpigmented, and hyperkeratotic plaques on the scalp; prominent nasal root; infraorbital creases with thin skin; malar hypoplasia; narrow nasal tip; low hanging columella; short and smooth philtrum; thin upper lip; high‐arched palate; bifid uvula; under folded helices with prominent antehelices dry and loose skin; keratosis pilaris and nail dystrophy. The systemic and neurological examinations were otherwise unremarkable. Hair examination revealed trichorrhexis nodosa and a typical “tiger‐tail” banding pattern under a polarized light microscope (Fig 2A). Sanger sequencing of the *MPLKIP* gene identified a homozygous frameshift variant [NM_138701.4:c61delT, p.(Trp21GlyfsTer132)] (Fig 2C).

Cranial MRI at 13 years and 3 months of age showed hyperintense lesions of the periventricular white matter on the frontal regions in T2‐FLAIR images. Echocardiography showed mild aortic and mitral valve insufficiency. Hormone workup at 14 years of age revealed a FSH level of 4.82 mIU/ml (normal: 3.6–12.6 mIU/ml), LH of 0.1 IU/ml (normal: 2.4–12.5 IU/ml), and an estradiol of 5 pg/ml (normal range: 10–100). Lumbar bone mineral density showed osteoporosis (*Z* score: −6.1, *T* score: −8.2). At 16 years of age, a pelvic ultrasound showed bilateral hypoplastic ovaries (1 cc on the right and 0.8 cc on the left at 16 years of age). 25‐OH vitamin D level was 21.3 ng/ml (20–29 ng/ml: insufficiency) suggestive for vitamin D insufficiency. She had low normal serum calcium levels (Ca level of 9.1 mg/dl; normal: 9–10.5 mg/dl), a high level of ALP (250 U/l; normal: 50–85 U/l), and a high level of phosphate (4.6 mg/dl; normal: 2.7–4.5). With these results, vitamin D replacement treatment was initiated and she was administered estradiol patches. Further workup comprising an audiogram, complete blood count, haemoglobin electrophoresis, and thyroid function tests were normal.

On the last follow‐up visit at age 19 years and 6 months, weight was 41 kg (−3.3 SD), height was 152 cm (−1.9 SD), and OFC was 52 cm (−3.3 SD). Follow‐up hormone studies at that age showed hypergonadotropic hypogonadism, with a FSH level of 22 mIU/ml (normal: 1.5–12.4 mIU/ml), LH level of 13.7 IU/ml (normal: 1.7–8.6 IU/ml), and Estradiol of 67.9 pg/ml (normal: 10–100 pg/ml) under estradiol patch treatment.

#### Subject TTD299IS

This boy (Fig 1E and F), the third living child of first‐degree cousins, was referred to the outpatient clinic due to global developmental delay with hair and nail abnormalities. The parents had history of two stillbirths, three neonatal deaths with unknown aetiology, and a child with hypotonia and growth retardation who died at 16 months of age (Fig 1E). He was born at term via caesarean section after an unfollowed pregnancy. Birth measurements were not recorded. He walked without support at the age of 7 years, spoke single words at 12 years, and built two‐word sentences at 20 years. He had a febrile convulsion at the age of 7 years. He was not examined by a professional, until he was referred to a paediatric neurologist at the age of 12 years due to global developmental delay. Cranial MRI, extensive metabolic screening, and electroencephalography showed no abnormalities. He had frequent vomiting at the age of 13 years, due to lower oesophageal sphincter deficiency, diagnosed by oesophagogastroscopy.

Physical examination at the age of 17 years and 9 months showed a weight of 50 kg (−2.82 SD), a height of 173 cm (−0.52 SD), and a OFC of 51 cm (−4.44 SD). He had brachycephaly; coarse and brittle hair; long face; sparse eyebrows and beard; long and curved eyelashes; malar hypoplasia; strabismus; prominent nasal root; infraorbital creases with thin skin; low hanging columella; short philtrum; thin upper and lower lip vermilions; narrow and high‐arched palate; single central incisor; and small ears. He showed keratosis pilaris, hyperkeratotic plaques on extensor surfaces of the upper extremities, and nail dystrophy. The gait was ataxic. The systemic and neurological examinations were otherwise unremarkable. Hair examination revealed trichorrhexis nodosa and a mild typical “tiger‐tail” banding pattern under a polarized light microscope. Sanger sequencing of the *MPLKIP* gene identified a homozygous frameshift variant [NM_138701.4:c.505insA, p.(Thr169AsnfsTer75)] (Fig 2C).

Hormone workup at 17 years of age showed normogonadotropic hypogonadism, with a FSH level of 4.3 mIU/ml (normal: 1.5–12.4 mIU/ml), LH level of 1.5 IU/ml (normal: 1.7–8.6 IU/ml), and testosterone level of 0.96 ng/ml (normal: 0.07–8 ng/ml). Follow‐up hormone studies at 26 years showed hypergonadotropic hypogonadism, with a FSH level of 26.9 mIU/ml (normal: 1.5–12.4 mIU/ml), LH level of 16.8 IU/ml (normal: 1.7–8.6 IU/ml), and testosterone level of 3.76 ng/ml (normal: 2.2–9 ng/ml). Penis length was normal, and testes were palpable in the scrotum with volumes of 8 cc each. Lumbar bone mineral density showed osteoporosis (*Z* score: −3.4).

### Hair microscopy

Scalp hair of six subjects was examined under light and polarized microscopy to detect the specific hair pattern in comparison with TTDN reports from the literature.

### Mutation screening

DNA was isolated from a 2‐ml peripheral blood sample by a solution‐based kit (DNA Isolation Kit for Mammalian Blood, Roche, Germany). Primers were designed to cover the two coding exons and exon‐intron boundaries for *MPLKIP* (NM_138701.4). BigDye® Terminator v3.1 Cycle Sequencing Kit (Thermo Fisher, USA) was used according to the instructions of the manufacturer. Pathogenicity evaluation was performed based on inheritance mode, database entries (HGMD, ClinVar, dbSNP), and novel alteration according to the American College of Medical Genetics and Genomics and the Association for Molecular Pathology (ACMG/AMP) criteria. Population frequency was also evaluated according to gnomAD data. The following primers were used to sequence genomic DNA: MPLKIP Exon 1 FWD: CACTTAAATCCACTGAGTCTCTCG, MPLKIP Exon 1 REV: CTGCAAAATTGGGCTAACAATAG, MPLKIP Exon 2 FWD: CAATGTGATTCCCGCTAACC, and MPLKIP Exon 2 REV: TTGCAAAACATTTACCTATGAACTC.

### Cell culture, transfection and auxin‐inducible degron system

Primary fibroblasts were cultured from skin biopsies from individuals TTD287IS, TTD288IS, TTD289IS, TTD290IS, TTD295IS, and TTD299IS. All primary fibroblasts, including NER‐deficient XP25RO (XP‐A); NER‐proficient C5RO and 296IS (primary fibroblasts from father TTD295IS); and NER‐proficient GFP‐expressing C5RO (CTR^GFP^; Theil *et al*, 2019), were cultured in Ham's F10 medium (BE02‐014F, Lonza) supplemented with 15% foetal bovine serum (S1810, Biowest) and 1% penicillin–streptomycin (P0781, Sigma‐Aldrich) at 37°C, 20% O_2_, and 5% CO_2_. SV40‐immortalized MRC‐5, SV40‐immortalized MCR‐5 MPLKIP‐GFP, HCT116, and HCT116 MPLKIP‐mAID‐mClover (clone #14 and #19) were cultured in 1:1 mix of DMEM (11965092, Gibco) and Ham's F10 medium (BE02‐014F, Lonza) supplemented with 10% foetal bovine serum (S1810, Biowest) and 1% penicillin–streptomycin (P0781, Sigma‐Aldrich) at 37°C, 20% O_2_, and 5% CO_2_. N/TERT keratinocytes (from James G. Rheinwald, Harvard Institute of Medicine): wild‐type control, *MPLKIP* knock out (KO #A10 and KO #16), and *MPLKIP* knock in (KI #7; p.Trp21Glyfs132Ter) were cultured under low confluence (< 40%) in keratinocyte serum‐free medium (KSFM medium, Invitrogen; Dickson *et al*, 2000). All cell lines are frequently tested for mycoplasma contamination and have been found to be free of contamination.

The following crRNAs were used to generate knock‐in and knock‐out cell lines: AGCAAUACUCAAACAUUCACAGGC for U2OS MPLKIP‐GFP or HCT116 MPLKIP‐mAID‐mClover, UGGUCCGGGUGGAGGAGGUU for *MPLKIP*‐deficient N/TERT keratinocytes #7 and #16, and CCUAGAACCAGUAUCUGUAG for *MPLKIP*‐deficient N/TERT keratinocyte #7.

Individual siRNAs were purchased from Horizon Discovery and transfected overnight using Lipofectamine RNAiMAX (Invitrogen), according to the manufacturer's instructions. siRNAs used were: control (CTR; D‐001210‐05‐20; UGGUUUACAUGUCGACUAA), DBR1 #6 (J‐008290‐06‐0002; GACAAAUGCUUACCACAUA) and DBR1 #7 (J‐008290‐07‐0002; CCAUGUAACUUUAGUGUAA).

To deplete MPLKIP‐mAID‐mClover proteins using the auxin‐inducible degron system, HCT116 cells were grown overnight in the presence of 0.4 μg/ml doxycycline (DOX). After DOX incubation, auxin was added, resulting in rapid degradation of MPLKIP‐mAID‐mClover proteins via poly‐ubiquitination, as previously described (Natsume *et al*, 2016).

### Colony‐forming ability/survival

Fibroblasts were plated in 10 cm dishes (1,500 fibroblasts/dish), in triplicate. After 24 h, fibroblasts were irradiated with different doses of UV‐C irradiation (0–8 J/m^2^) and incubated for approximately 2 weeks. Colonies were fixed and stained with 0.1% Brilliant Blue R (Sigma) and counted (Gelcount, Oxford Optronix Ltd.). The survival was plotted as the percentage of colonies obtained after treatment compared to the mean number of colonies from the mock‐treated fibroblasts (set at 100%).

### Unscheduled DNA synthesis (UDS) assay

NER‐proficient GFP‐expressing C5RO fibroblasts (CTR^GFP^) were mixed with NER‐proficient fibroblasts C5RO (CTR), *MPLKIP*‐deficient fibroblasts, or NER‐deficient fibroblasts XP25RO (XP‐A) and seeded onto 24 mm coverslips. Two days later adherent fibroblasts were washed with PBS and UV‐C irradiated with 16 J/m^2^. Thereafter, fibroblasts were incubated for 3 h in medium containing 0.2 μM 5‐ethynyl‐2′‐deoxyuridine (EdU, A10044, Invitrogen) and 1 μM FUDR (Sigma). After EdU incorporation, fibroblasts were fixed in 3.7% formaldehyde/PBS + 0.5% Triton, washed with PBS^+^ (PBS containing 0.15% glycine and 1% BSA), permeabilized 20 min in 0.5% Triton/PBS, and rinsed with PBS^+^. Samples were incubated for 30 min with fluorescent dye coupling buffer containing 10 mM CuSO_4_ and Atto 594 azide (AD 594‐105, ATTO‐TEC), washed for 20 min in 0.5% Triton X‐100/PBS, rinsed in PBS^+^. For visualizing GFP‐expressing fibroblasts, samples were incubated for 3 h with monoclonal GFP antibody (11814460001, Roche) diluted 1:1,000 in PBS^+^ in a moist chamber, washed for 20 min in 0.5% Triton/PBS, and rinsed with PBS^+^. Samples were incubated for 1 h with Alexa Fluor 488 conjugated secondary antibodies (A11001, Invitrogen, dilution 1:1,000) and 0.1 μg/ml DAPI (D9542, Sigma) diluted in PBS^+^ in a moist chamber, washed 20 min in PBS/Triton X‐100, and rinsed in PBS. Samples were mounted using Aqua‐Poly/Mount (18606, Polysciences) and imaged using an LSM700 microscope equipped with a 40× Plan‐apochromat 1.3 NA oil immersion lens (Carl Zeiss). UDS levels were expressed as the average fluorescence intensity in the nucleus of the tested fibroblasts versus those measured in GFP‐expressing fibroblasts, which was set at 100%. The mean fluorescence is determined with a confocal microscope (Zeiss LSM 700) from at least 40 fibroblasts and three independent experiments. Images were processed using ImageJ.

### Immunofluorescence

For immunofluorescence experiments, cells were seeded on coverslips, fixed with 2% paraformaldehyde, washed with PBS^+^ (PBS containing 0.15% glycine and 0.5% BSA), permeabilized for 20 min in 0.1% Triton/PBS and rinsed with PBS^+^. Samples were incubated for 2 h with primary antibody diluted in PBS^+^ in a moist chamber, washed for 20 min in 0.1% Triton/PBS, and rinsed with PBS^+^. Samples were incubated for 1 h with secondary antibodies and 0.1 μg/ml DAPI (D9542, Sigma) diluted in PBS^+^ in a moist chamber, washed for 20 min in PBS/Triton X‐100, and rinsed in PBS. Samples were mounted using Aqua‐Poly/Mount (18606, Polysciences) and imaged using an LSM700 microscope equipped with a 40× Plan‐apochromat 1.3 NA oil immersion lens (Carl Zeiss). Primary antibodies used were against MPLKIP (ab34309, Abcam, dilution 1:1,000) and DBR1 (Sigma, HPA035365, dilution 1:1,000). Secondary antibody used was Alexa Fluor 594 goat anti‐mouse (A11032, Invitrogen, dilution 1:1,000) and Alexa Fluor 594 goat anti‐rabbit (A11012, Invitrogen, dilution 1:1,000).

### RNA isolation and real‐time quantitative PCR (RT‐qPCR)

Total RNA was extracted from primary fibroblasts and the whole FTMs with the RNEasy Kit (Qiagen). Sufficient amounts of total RNA were available for cDNA synthesis using the iScript™ cDNA Synthesis Kit (Bio‐Rad) according to the manufacturer's instructions. All primers were designed and evaluated with the amplification efficiency (determined by a dilution range of cDNA) and specificity (determined by gel electrophoresis). For all qPCR reactions, an IQ SYBR Green Supermix (BioRad) was used. qPCR was performed by using a CFX384 system (BioRad) according to the PCR program as described before (Hogervorst *et al*, 2018). The reference genes were selected based on stability using the Genorm program. The expression analysis was performed within the BioRad Software (CFX manager). Primers used for qPCR are: DBR1 FWD: GGAAACCATGAAGCCTCAAA, DBR1 REV: CCGATCCTTACACCTCGGTA, TUBG2 FWD: ACATGAACAATGACCTCATCG, and TUBG2 REV: ATCCTCTGCAGGCTCTTGTG.

### Immunoprecipitation

Immunoprecipitation was performed as previously described (Pines *et al*, 2018). Briefly, cell lysate from a 2 × 14.5 cm dish (80% confluent) was prepared using IP buffer (30 mM Tris pH 7.5, 150 mM NaCl, 2 mM MgCl2, 0.5% Triton X‐100, and protease inhibitor cocktail [Roche]) supplemented with 250 U/ml Benzonase® nuclease. XAB2 was precipitated by anti‐XAB2 (anti‐HCNP, sc‐271037, Santa Cruz Biotechnology, 0.6 μg antibody used) and GFP‐tagged (as well as mClover‐tagged) proteins were precipitated by GFP‐Trap®_A beads (Chromotek). Fractions were then analysed by immunoblot or mass spectrometry (see below).

### Immunoblot

To prepare cell lysates, cells were collected in 2× sample buffer (125 mM Tris–HCl pH 6.8, 20% Glycerol, 10% 2‐β‐Mercaptoethanol, 4% SDS, and 0.01% Bromophenol Blue) and boiled at 98°C for 5 min. Protein lysate was separated by SDS‐PAGE and transferred to a PVDF membrane (0.45 μm, Merck Millipore). The membrane was blocked in 3% BSA and then incubated overnight with the primary antibody. The membrane was washed three times in 5 min with 0.1% Tween/PBS and incubated 1 h with secondary antibodies, and washed three times in 5 min with 0.1% Tween/PBS. Primary antibodies used were anti‐MPLKIP (anti‐TTDN1, sc‐393079, Santa Cruz, dilution 1:1,000), anti‐DBR1 (HPA035365, Sigma, dilution 1:1,000), anti‐GFP (11814460001, Roche, dilution 1:1,000), anti‐H2B (07–371, Millipore, dilution 1:1,000), anti‐XAB2 (anti‐HCNP, sc‐271037, Santa Cruz Biotechnology, dilution 1:2,000), anti‐AQR (anti‐IBP160, A302‐547A, Bethyl Laboratories, dilution 1:2,000), anti‐Cyclin H (MA3‐025, Thermo Scientific, dilution 1:2,000), anti‐CWF19L1 (ab150842, Abcam, dilution 1:1,000), anti‐PRPF6 (A302‐773A, Bethyl Laboratories, dilution 1:1,000), anti‐PRPF8 (sc‐30207, Santa Cruz Biotechnology, dilution 1:1,000), anti‐PRPF31 (ab188577, Abcam, dilution 1:1,000), anti‐PLK1 (33‐1700, Thermo scientific, dilution 1:1,000) and anti‐GTF2H1 (WH0002965M1, Sigma, dilution 1:1,000). Secondary antibodies were conjugated with CF IRDye 680 (sab4600215, Sigma, dilution 1:10,000) and CF IRDye 770 (sab4600199, Sigma, dilution 1:10,000) and visualized using the Odyssey CLx Infrared Imaging System (LI‐COR Biosciences).

### Identification of MPLKIP interactors by SILAC‐based MS analysis

Sample treatment was performed as previously described (Pines *et al*, 2018). For SILAC, cells were cultured at 37°C, 20% O_2_, and 5% CO_2_ in DMEM containing 10% dialysed FBS (Gibco), 10% GlutaMAX (Life Technologies), 1% penicillin–streptomycin (P0781, Sigma‐Aldrich), unlabelled L‐arginine‐HCl, and L‐lysine‐HCL (control, “light”) or ^13^C_6_,^15^N_4_l‐arginine‐HCl and ^13^C_6_,^15^N_2_l‐lysine‐2HCl (MPLKIP‐GFP, “heavy”) (Cambridge Isotope Laboratories). For immunoprecipitation, cells were incubated for 10 min on ice in HEPES buffer containing 30 mM HEPES pH 7.5; 130 mM NaCl; 1 mM MgCl2; 0.5% Triton X‐100; 1× EDTA‐free Protease Inhibitor Cocktail (Roche). After 10 cycles of sonication using the Bioruptor Sonicator (Diagenode) (15 s on; 45 s off) at 4°C, 500 U Benzonase® nuclease (Merck Millipore) was added and samples were kept in rotation for 1–2 h at 4°C. The insoluble fraction was pelleted at 13,200 r.p.m. for 10 min at 4°C, and the soluble fraction was applied for immunoprecipitation for 90 min at 4°C, using 25 μl of slurry GFP‐Trap®A beads (Chromotek). Bound proteins were eluted with SDS–PAGE loading buffer and separated on 4–12% Bis‐Tris NuPAGE® gels (Invitrogen). Lanes were cut into 2‐mm slices and subjected to in‐gel reduction with dithiothreitol, alkylation with iodoacetamide (98%; D4, Cambridge Isotope Laboratories) and digested with trypsin (sequencing grade; Promega). To perform nanoflow liquid chromatography tandem mass spectrometry (LC–MS/MS), an 1,100 series capillary liquid chromatography system (Agilent Technologies) coupled to a Q‐Exactive mass spectrometer (Thermo Scientific) operating in positive mode was used. ReproSil C18 reversed phase column (1.5 cm × 100 μm) at a rate of 8 μl/min was used to trap the peptide mixtures that was then separated by a linear gradient of 0–80% acetonitrile (in 0.1% formic acid) during 60 min at a rate of 200 nl/min using a splitter. The eluate was sprayed into the electrospray ionization (ESI) source of the mass spectrometer and spectra were acquired in continuum mode while a data‐dependent mode was used to perform fragmentation of the peptides. MaxQuant software (version 1.5.4.1) and Perseus software (version 1.6.14.0) were used to analyse the data. The mass spectrometry proteomics data have been deposited to the ProteomeXchange Consortium via the PRIDE (Perez‐Riverol *et al*, 2022) partner repository with the dataset identifier PXD044434.

### Dermal equivalents

Dermal equivalents were generated as described earlier (Wu *et al*, 2022). In short, 1 ml of cell‐free collagen (1 mg/ml) solution was pipetted into a six‐well‐filter insert (Corning Life Sciences, Tewksbury, MA). After polymerization, 3 ml of fibroblast‐populated (0.4 × 10^5^ fibroblasts/ml) collagen (2 mg/ml) solution was pipetted onto the previous collagen layer. After polymerization, the dermal equivalents were submerged in medium consisting of Dulbecco's modified Eagle's medium (Gibco), 5% foetal calf serum, and 1% penicillin–streptomycin (P0781, Sigma‐Aldrich). The medium was refreshed twice a week. Dermal equivalents were cultured under submerged conditions for 3 days before seeding the N/TERT keratinocytes.

### Generation of human skin equivalents (HSEs)

HSEs were generated by seeding 0.25 × 10^6^ N/TERT keratinocytes onto the dermal equivalent, as described elsewhere (Van Drongelen *et al*, 2014). Briefly, after 2 days of submerged culturing in medium containing 5% FCS, the FCS concentration was reduced to 1% for one additional day. Subsequently, HSEs were lifted to the air‐liquid interface and cultured for 14 days with FCS‐free medium supplemented with 1 ng/ml EGF, 3 ng/ml KGF, 2 μM L‐serine, 10 μM L‐carnitine, 1 μM DL‐α‐ tocopherol‐acetate, 50 μM ascorbic acid, a free fatty acid supplement which contained 25 μM palmitic acid, 30 μM linoleic acid and 7 μM arachidonic acid, and 2.4 × 10^−5^ M bovine serum albumin. The culture medium was refreshed twice a week.

### Morphological and immunohistochemical analysis of HSEs

From the HSEs, one part was snap‐frozen in liquid nitrogen while the other part was fixed in 4% paraformaldehyde, dehydrated, and paraffin embedded. Global histological analysis was performed on 5‐μm sections through haematoxylin and eosin (HE) staining. For fluorescence staining, sections were labelled with the primary antibody and counterstaining was performed with DAPI. Primary antibodies used: anti‐Ki67 (Clone MIB1, Dako, Glostrup, Germany, dilution 1:1,000), anti‐KRT10 (Clone DE‐K10, Labvision/neomarkers, California, USA, dilution 1:1,000), anti‐LOR (Clone AF62, Covance, USA, dilution 1:1,000), and anti‐FLG (Clone FLG01, Thermo Fisher Scientific, dilution 1:1,000). Secondary antibodies included Goat Anti‐Mouse IgG Cy3 (Jackson immunoresearch, dilution 1:1,000), Goat Anti‐Rabbit IgG Cy3 (Jackson immunoresearch), and Donkey anti‐Rabbit IgG Alexa 488 (Invitrogen, dilution 1:1,000).

### Quantification of epidermal thickness, cell proliferation index, and stratum corneum

The epidermal thickness was determined through counting the viable layers in 6–8 images of each HSE of various tissue regions after HE staining with 200x magnification. The proliferation index was determined by counting the number of Ki67‐positive nuclei among the total number of cells in the basal layer. A minimum of 100 basal cells were counted at three different regions of each section. The resulting proliferation index is the percentage of positive stained nuclei. For both estimations, the data are presented as the mean of three independent experiments ± SD. Counting of the stratum corneum layers was performed on 5‐mm sections that were stained for 1 minute with a 1% (w/v) safranin O (Sigma) solution dissolved in Millipore water. After water washout, a 2% (w/v) KOH solution was applied on the sections for 25 min to swell the corneocytes. After removal of the KOH solution, the glass slides were washed with Millipore water and enclosed with Kaiser's glycerine. Layers were counted at 400× magnification.

### RNA sequencing analysis on *MPLKIP* ‐deficient HSEs

Total RNA was isolated in triplo from 3 *MPLKIP*‐deficient epidermis (KI #7) and 3 normal control epidermis (CTR) with the RNeasy Lipid Tissue Mini Kit (QIAGEN). RNA quantity and quality were evaluated using the NanoDrop 8000 spectrophotometer and (Thermo Scientific) and the Agilent 2100 Bioanalyzer (Agilent Technologies), respectively. RNA samples were prepped at the Human Genomics Facility of the Genetic Laboratory of the Department of Internal Medicine at Erasmus MC with the CORALL mRNA‐Seq v2 Library Prep Kit and sequenced on an Illumina NovaSeq 6000. An average of 30–40 M 2 × 150 bp reads were sequenced per sample. We used STAR v2.7.10a (Dobin *et al*, 2013) to align the samples against the human reference GRCh38 with GENCODE v38 annotation, followed by Picard MarkDuplicates v2.27.5 (Github, 2019). Quality control was assessed by fastqc v0.11.9 (https://github.com/s‐andrews/FastQC/), RSeQC v5.0.1 (Wang *et al*, 2012, 2016), and MultiQC v1.15.dev0 (Ewels *et al*, 2016). All samples passed quality control, and contained on average, 20 M unique mapping reads. R v4.1.2 (R Foundation for Statistical Computing, Vienna, Austria. https://www.R‐project.org/) (R Core Team, 2021) was used for downstream analysis. Principal Component Analysis showed that all samples clustered sufficiently well together. No samples were discarded from the analysis.

Data was analysed as follows:

#### Differential gene expression (DESeq2)

Read counts overlapping the entire gene body were generated using htseq‐count v2.0.2 (Putri *et al*, 2022). We used the R package DESeq2 v1.34.0 (Love *et al*, 2014) to perform differential gene expression analysis between *MPLKIP*‐deficient samples and controls. We deemed as significant all genes with adjusted *P*‐value < 0.05 and at least 50% change in expression (absolute log_2_ Fold Change ≥ log_2_(1.5)). The maximum transcript length and number of exons among all the isoforms in each gene were derived from the annotation file. The statistical significance of the differences in transcript length and number of exons was obtained with a two‐sided Mann–Whitney test.

#### Differential transcript expression (swish)

We used kallisto v0.46.1 (Bray *et al*, 2016) to generate pseudoalignments to the reference genome and annotation file directly from the fastq files. We used the Swish method (R package Fishpond v2.0.1; Zhu *et al*, 2019) to measure differential transcript expression. We deemed as significant all transcripts with adjusted *P*‐value < 0.05 and abs (log_2_ Fold Change) > log_2_(1.5).

#### Differential exon expression (DEXSeq)

We used DEXSeq v1.40.0 (Anders *et al*, 2012; Reyes *et al*, 2013) to analyse differential exon expression. We followed their recommended protocol to adapt the annotation file by collapsing the exons present in multiple transcripts and to count the reads overlapping such fragments. We deemed as significant all exons with adjusted *P*‐value < 0.05 and abs (log_2_ Fold Change) > log_2_(1.5).

#### Alternative splicing (rMATS)

We analysed alternative splicing events with rMATS‐turbo v4.1.2. (Shen *et al*, 2014). We ran rMATS on the STAR‐generated alignment files using the GENCODE v38 annotation and the parameters ‐t paired ‐‐readLength 151 ‐‐nthread 8 ‐‐allow‐clipping. Additionally, we ran the analysis with and without the parameter novelSS to detect novel aberrant or not‐annotated splicing events.

### Lariat detection

We developed a pipeline to detect lariats adapting a previously published method (Pineda & Bradley, 2018), which, in time, was adapted from a split‐read alignment strategy (Mercer *et al*, 2015). The basis of this method for detecting circular fragments of introns is detecting reads overlapping the branching point of the lariat, commonly an Adenine (Gao *et al*, 2008). In reads overlapping such points the 3′ fragment of the intronic sequence will be found upstream of the 5′ intronic fragment, strongly suggesting of a circular lariat.

#### Intronic sequence

First, we built a database of introns. The occasional presence of overlapping genes and/or multiple transcripts in a gene using alternative 5′ and 3′ splicing sites can lead to duplicated or overlapping non‐unique intronic sequences. To prevent this, we generated a list of exclusively intronic regions by subtracting from the gene's body the coordinates of any exon from any gene in a strand‐aware manner.

Next, we built two FASTA files, one containing the first 20 nt immediately downstream from the 5′ splicing site and the other containing the 250 nt immediately upstream from the 3′ splicing site, in a strand‐aware manner. We removed from the data any intronic region shorter than 300 bp to prevent overlapping sequences between the 5′ and 3′ ends. During this processing, we kept in each FASTA read header information about the gene of origin, strand, and genomic coordinates to later easily identify the sequence's origins.

#### Mapping the 5′ splice sites to the sample's reads

Starting from the alignments produced for the differentially expressed genes analysis, for each sample we obtained their unmapped reads. We then used seqkt v1.4 (https://github.com/lh3/seqtk) to remove reads with > 5% ambiguous reads. Next, we aim to map the database of 5′‐intronic 20 nt fragments to our samples' unmapped reads. To do so, we used bowtie2 v2.5.1 (https://doi.org/10.1038/nmeth.1923) to build an index for our samples' unmapped reads, and we mapped the 5′ splicing sites to this index using the command: bowtie2 ‐x <sample_reads_index> ‐‐end‐to‐end ‐‐sensitive ‐‐k 10000 ‐‐no‐unal ‐‐no‐sq ‐p 8 ‐f ‐U <intronic_5'ss_fasta>. We filtered these results to keep for further analysis only those reads where the alignment had no mismatches, no indels, and where the reads of our sample map only to a single intronic 5'splice site.

Next, we trimmed off the intronic 5′ splicing site sequence from the reads to leave only the 3′‐intronic side of these putatively‐lariat reads and further analyse them. Our samples were prepared using the CORALL mRNA‐Seq v2 Library Prep Kit. This protocol generates paired‐end stranded reads where the 1^st^ mate of the pair (read1) is a copy of the original RNA template, while the second mate (read2) is in the complementary strand. We leveraged this information to introduce an improvement not described in the original protocol: we used the mate of origin information of each read to trim the sequence in the proper direction (5′ to 3′ in read1, 3′ to 5′ in read2). We also trimmed the UMI barcode from the first 12 nt from read1. Finally, we removed from further analysis any read with < 20 nt remaining after trimming.

#### Mapping to the 3′ splice sites

We built a bowtie index for the intronic 3′ splice site 250 nt sequences. We then mapped the remaining trimmed reads from our samples to these 3′ introns with the command: bowtie2 ‐x <intronic_3'ss_index> ‐‐end‐to‐end ‐‐sensitive ‐k 10 ‐‐no‐unal ‐‐no‐sq ‐f ‐U <sample_trimmed_reads>. We filtered these results to remove reads with > 5 mismatches, a mismatch rate > 10%, or an indel longer than 3 bp. We then restricted these remaining reads to those mapping only to a single intronic 3′ splicing site, and, among those, only the reads mapping to the same gene and strand in the 3′ and 5′ splicing sites, and where the mapped introns were in the proper order, in a strand aware manner. As a novelty for our adaptation of the previously published method, we used the read pair mate information available in our reads to make sure that the reads actually originated from a lariat sequence. We leveraged the mate information from the read, the annotated strand of the gene in the reference, and the strandness of the mapping of the read against the intron's 5′/3′ splicing sites. For reads originating from mate 1, we only kept those where the gene annotated strand and the mapping strand matched; and for reads from mate 2, we only kept those where the strand information of the gene's and the mapping was opposite. A detailed illustration of the lariat analysis can be seen in Fig EV6.

### Label‐free mass spectrometry analysis on *MPLKIP* ‐deficient epidermis

Protein lysates from three biological replicas and isolated epidermal layers from HSEs were prepared as described previously (Huttlin *et al*, 2010). Briefly, tissues were homogenized in a Bioruptor (Diagenode) in urea lysis buffer (8 M urea, 25 mM Tris–HCl pH 8, 100 mM sodium chloride) containing a phosphatase inhibitor (Sigma) and a protease inhibitor cocktail (Roche). Crude lysates were benzonase treated, and insoluble material was removed by centrifugation. Protein quantitation was performed using the colorimetric absorbance BCA protein assay kit (Thermo). Proteins were reduced using 5 mM 1,4‐dithiothreitol for 30 min at 50°C and subsequently alkylated using 10 mM iodoacetamide for 15 min in the dark. Proteins were first digested for 4 h with Lys‐C (Wako Pure Chemicals; 1:200 enzyme:substrate ratio) and then overnight with trypsin (Thermo; 1:50 enzyme:substrate ratio) at 30°C.

### Nanoflow liquid chromatography tandem mass spectrometry (nLC–MS/MS)

Extracted proteolytic peptides were analysed by nanoflow LC–MS/MS. nLC‐MS/MS was performed on an EASY‐nLC 1200 coupled to an Orbitrap Fusion Lumos Tribrid mass spectrometer (Thermo Scientific) operating in positive mode and equipped with a nanospray source. Peptides were separated on a ReproSil C18 reversed phase column (Dr Maisch GmbH; column dimensions 15 cm × 50 μm, packed in‐house) using a linear gradient from 0 to 80% B (A = 0.1% formic acid; B = 80% (v/v) acetonitrile, 0.1% formic acid) in 180 min and at a constant flow rate of 200 nl/min using a splitter. The column eluent was directly sprayed into the ESI source of the mass spectrometer. The MS1 acquisition parameters were set as follows: full scan range, 300–1,650 *m*/*z*; MS1 resolution, 120,000; AGC, 4E5; maximum injection time, 50 ms; wide quad isolation, true. MS data acquisition in DIA (data‐independent analysis) mode was performed using 64 variable windows covering a mass range of 300–1,400 *m*/*z*. The MS2 acquisition settings were set as follows: MS2 resolution, 30,000; AGC, 5E4; maximum injection time, 54 ms. The normalized collision energy (NCE) was set to 30%, and the overall cycle time was 3 s.

#### Data analysis

Data were analysed with Spectronaut (Biognosys), and output was processed in Perseus (version 1.6.14.0). The search parameters of Spectronaut (version 16.2.220903.53000) were set as follows: decoy generated method, mutated; precursor PEP cutoff 0.2; precursor q value cutoff 0.01; protein q value cutoff 0.01 at experiment level and 0.05 at run level; precursor filtering set to Q value; single hit definition by stripped sequence. The LFQ method was MaxLFQ and no cross‐run normalization was used. IDpicker was used as the protein inference algorithm. Downstream analysis including t testing was performed in Perseus (MaxQuant software suite). Proteins with ≥ 2 unique identified peptides were visualized by volcano plot, and t‐test analysis was performed by permutation‐based FDR calculation (*P* < 0.001). The mass spectrometry proteomics data have been deposited with the ProteomeXchange Consortium via the PRIDE (Perez‐Riverol *et al*, 2022) partner repository with the dataset identifier PXD044779.

### Statistics

Statistical analysis was performed using an ordinary one‐way ANOVA or a Mann–Whitney‐test in Graph Pad Prism version 8.2.1 for Windows (GraphPad Software, La Jolla, California, USA). *P* values expressed as <0.05 were considered to be significant, otherwise as not significant (ns). Data are presented as mean values, as well as each individual value, and SD error bars are shown for each experiment. The experiments were all based on at least three biological replications, which are specifically mentioned in the respective figure legends.

R v4.1.2 (R Foundation for Statistical Computing, Vienna, Austria, https://www.R‐project.org/) (R Core Team, 2021) was used for downstream analysis of RNA sequencing data and specifically mentioned in the respective figure legends and Materials and Methods. *P* values expressed as <0.05 and abs (log_2_ Fold Change) > log_2_(1.5) were considered to be significant, otherwise as not significant (ns).

The statistical analysis of the proteomics data in Fig 9B was performed with Spectronaut (Biognosys), and the output was processed in Perseus (version 1.6.14.0) and visualized in a volcano plot where the significant data points were determined by permutation‐based FDR calculation (*P* < 0.001). In Fig 3B, the proteins were classified as specific MPLKIP interactors (marked in colours) when log_2_ (SILAC ratio) > 0.92 (indicated by dashed vertical line).

## Author contributions

**Wim Vermeulen:** Conceptualization; resources; supervision; funding acquisition; writing – original draft; project administration; writing – review and editing. **Hannes Lans:** Resources; writing – review and editing. **Umut Altunoglu:** Resources; data curation; formal analysis; validation; investigation; visualization; writing – original draft; writing – review and editing. **Tuğba Kalayci:** Resources; data curation; formal analysis; validation; investigation; visualization; writing – original draft; writing – review and editing. **Sriram Sridharan:** Software; formal analysis; writing – review and editing. **Sabine EJ Tanis:** Data curation; software; formal analysis; investigation; methodology; writing – review and editing. **Zehra O Uyguner:** Data curation; formal analysis; validation; investigation; writing – review and editing. **Marion H Rietveld:** Data curation; formal analysis; validation; investigation; writing – review and editing. **Klaas W Mulder:** Resources; methodology; writing – review and editing. **Anja Raams:** Data curation; formal analysis; validation; investigation; writing – review and editing. **Joris Pothof:** Resources; funding acquisition; methodology; writing – review and editing. **Jan HJ Hoeijmakers:** Resources; funding acquisition; writing – review and editing. **José M Heredia‐Genestar:** Conceptualization; data curation; software; formal analysis; validation; investigation; methodology; writing – original draft; writing – review and editing. **Jeroen AA Demmers:** Resources; data curation; formal analysis; validation; investigation; methodology; writing – review and editing. **Hülya Kayserili:** Data curation; formal analysis; validation; investigation; writing – review and editing. **Nesimi Büyükbabani:** Data curation; formal analysis; validation; investigation; writing – review and editing. **Birsen Karaman:** Data curation; formal analysis; validation; investigation; writing – review and editing. **Arjan F Theil:** Conceptualization; data curation; formal analysis; validation; investigation; visualization; methodology; writing – original draft; project administration; writing – review and editing. **Alex Pines:** Data curation; formal analysis; validation; investigation; methodology; writing – review and editing. **Abdoelwaheb El Ghalbzouri:** Resources; funding acquisition; methodology; writing – review and editing.

## Disclosure and competing interests statement

The authors declare that they have no conflict of interest.

## For more information

Authors' homepage: www.vermeulenlab.com.

## Supporting information



Expanded View Figures PDF

Table EV1

Source Data for Expanded View

PDF+

Source Data for Figure 2

Source Data for Figure 3

Source Data for Figure 4

Source Data for Figure 5

Source Data for Figure 6

Source Data for Figure 7

Source Data for Figure 8

Source Data for Figure 9

## Data Availability

The datasets produced in this study are available in the following databases:
RNA‐Seq data are available via Sequence Read Archive (SRA), BioProject ID PRJNA1004451 (https://www.ncbi.nlm.nih.gov/sra/?term=PRJNA1004451). Proteomics data are available via ProteomeXchange with identifier PXD044779 for the SILAC‐MS data (https://www.ebi.ac.uk/pride/archive/projects/PXD044779) and PXD044434 for the nLC‐MS/MS data (https://www.ebi.ac.uk/pride/archive/projects/PXD044434).
