# Supplementary material for: Trichothiodystrophy‐associated MPLKIP maintains DBR1 levels for proper lariat debranching and ectodermal differentiation
Source: EMBO Mol Med. 2023 Oct 6;15(11):e17973. doi: 10.15252/emmm.202317973 (PMC10630875; doi:10.15252/emmm.202317973)
Supplement: Supplementary file 1 — Expanded View Figures PDF [file EMMM-15-e17973-s013.pdf]

Expanded View Figures

Figure EV1. Molecular analysis of *MPLKIP* variants in family members.

Sequence chromatograms are shown for the indicated family members. Coloured boxes show the position of the altered nucleotide sequence.

Source data are available online for this figure.

© 2023 The Authors

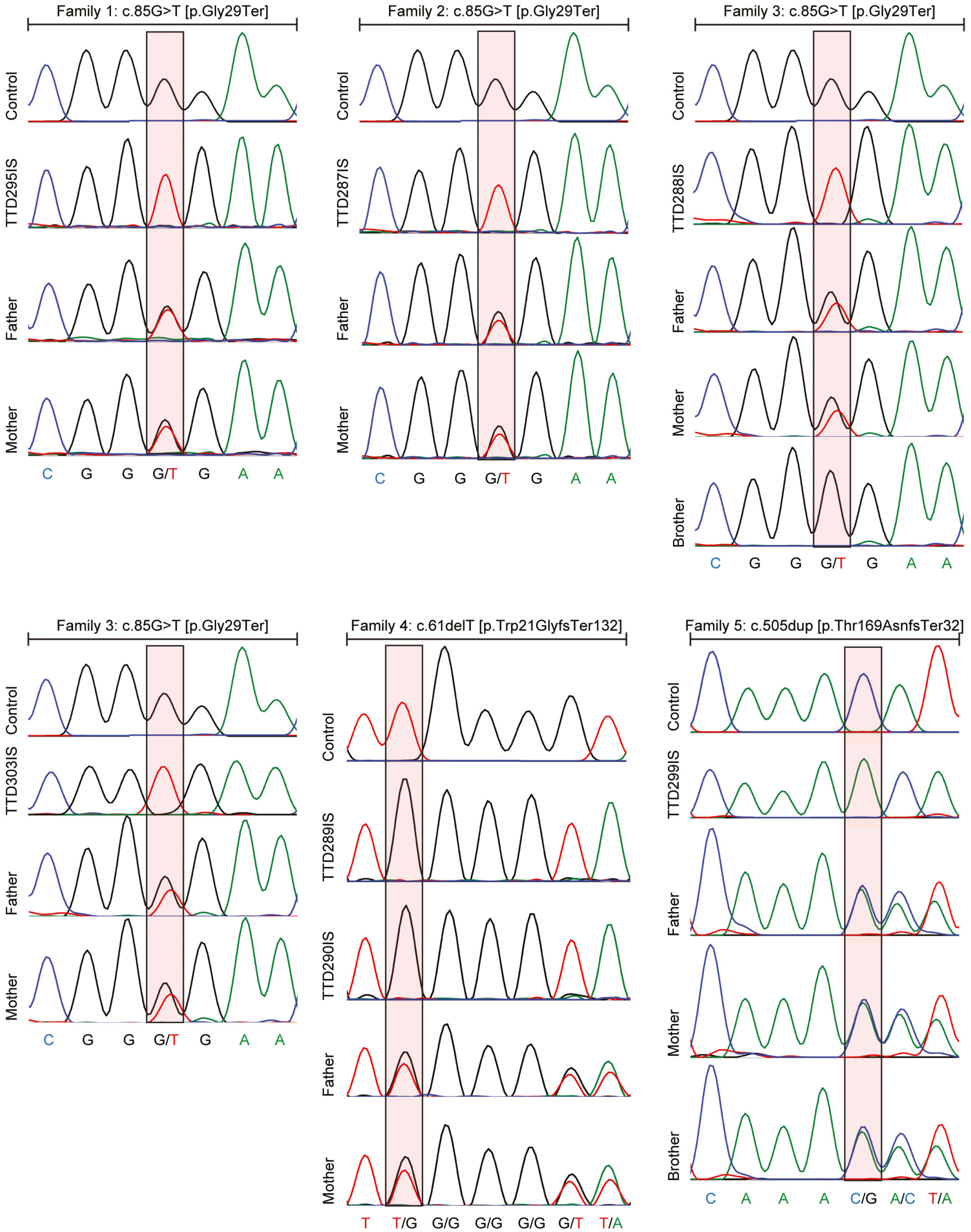
Family 1: c.85G>T [p.Gly29Ter]
Control
TTD295IS
Father
Mother
C G G G/T G A A
Family 2: c.85G>T [p.Gly29Ter]
Control
TTD287IS
Father
Mother
C G G G/T G A A
Family 3: c.85G>T [p.Gly29Ter]
Control
TTD288IS
Father
Mother
Brother
C G G G/T G A A
Family 3: c.85G>T [p.Gly29Ter]
Control
TTD303IS
Father
Mother
C G G G/T G A A
Family 4: c.61delT [p.Trp21GlyfsTer132]
Control
TTD289IS
TTD290IS
Father
Mother
T T/G G/G G/G G/G G/T T/A
Family 5: c.505dup [p.Thr169AsnfsTer32]
Control
TTD299IS
Father
Mother
Brother
C A A A C/G A/C T/A

Figure EV1.

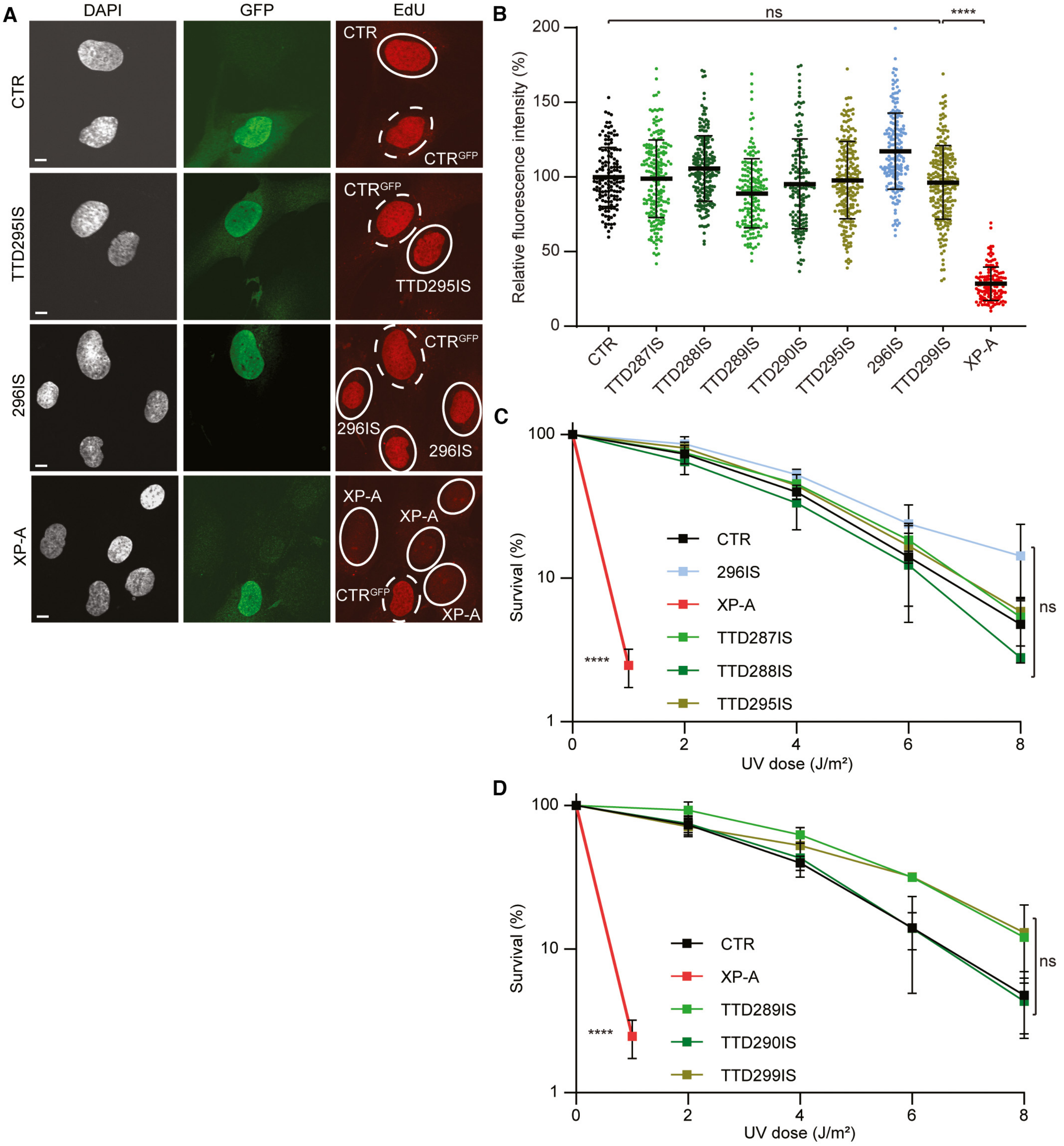

Figure EV2.

© 2023 The Authors

Figure EV2. *MPLKIP*-deficient fibroblasts are DNA repair proficient.

A Representative pictures from the UV-induced unscheduled DNA synthesis (UDS) experiment performed on primary fibroblasts from MPLKIP-deficient TTD295IS, the father of TTD295IS (296IS), NER-deficient XP25RO (XP-A), and NER-proficient control (CTR). Wild-type control fibroblasts that stably express GFP (CTRGFP, dashed circles) were mixed with the test fibroblasts (dashed circles). Global NER activities were measured using EdU incorporation after UV-irradiation, visualized by fluorescence-conjugated azide (Click-iT assay), and subsequently stained for GFP and DNA (DAPI). Scale bars: 20 μm.

B Mean UDS-derived fluorescence intensities of at least 50 nuclei were expressed as percentage of the mean intensity in CTR fibroblasts assayed in parallel (n = 3 biological replicates).

C, D Clonogenic UV survival to measure UV sensitivity. One day after seeding, primary fibroblasts were irradiated with the indicated doses of UV, and cultures were incubated for 2 weeks to grow colonies. Survival was blotted as a percentage of colonies obtained after treatment compared to mock-treated fibroblasts, set at 100% (n = 3 biological replicates).

Data information: (B). Data are represented as mean ± SD, ordinary one-way ANOVA. ****P < 0.0001; ns, not significant. (C, D). Data are represented as mean ± SD, nested one-way ANOVA. ****P < 0.0001; ns, not significant.
Source data are available online for this figure.

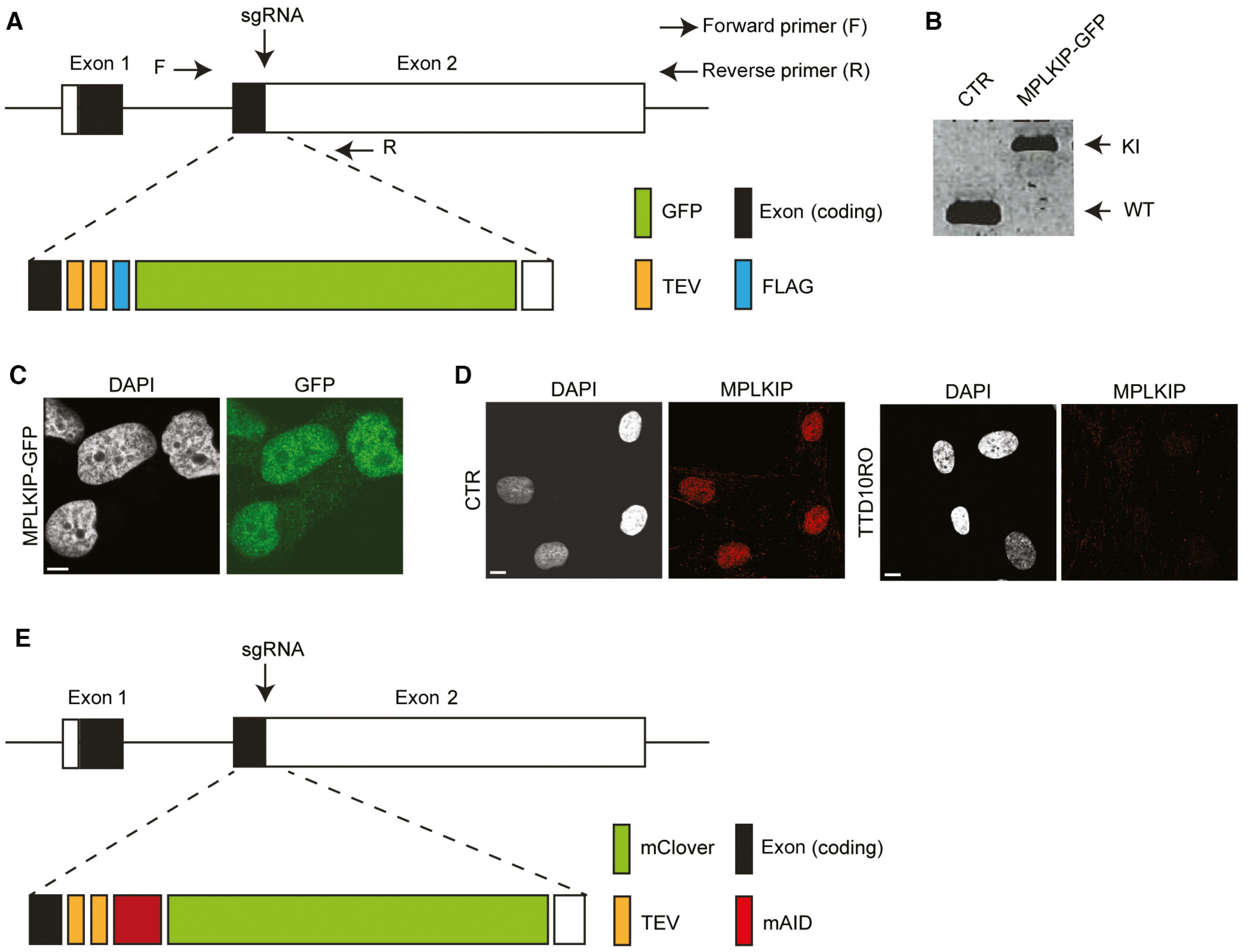

Figure EV3.

Figure EV3. Generation of fluorescently tagged *MPLKIP* knock-in cells.

A Schematic presentation of a knock-in strategy to target the coding sequencing of enhanced GFP at the 3′ end of the coding sequencing of the MPLKIP gene using the CRISPR-Cas9 technology in SV40-immortalized MRC-5 cells. The dotted line indicates the DNA fragment TEV(2x)-FLAG-GFP targeting construct containing homology arms on both sides. The translational stop codon (in exon 2) was mutated to allow in-frame fusion with the fluorescent marker and additional FLAG and TEV (2x) tags.

B PCR amplification of genomic DNA from MRC-5 (CTR) cells and MRC-5 MPLKIP-TEV(2x)-FLAG-GFP (MPLKIP-GFP) knock-in cells, forward (F) and reversed (R) PCR primers are indicated in panel S2A.

C Representative immunofluorescence analysis of MRC-5 cells expressing MPLKIP-GFP. DNA was stained with DAPI. Scale bars: 10 μm

D Representative immunofluorescence analysis was stained for MPLKIP and DNA (DAPI) to determine steady-state protein amounts in control fibroblasts (CTR) and MPLKIP-deficient primary fibroblast TTD10RO. Scale bars: 20 μm

E Schematic presentation of a knock-in strategy to target the coding sequencing of mClover in frame fused to the mAID tag at the 3′ end of the coding sequencing of the MPLKIP gene using the CRISPR-Cas9 technology in HCT116 cells. The dotted line indicates the DNA fragment TEV(2x)-mAID-mClover targeting construct containing homology arms on both sides. The translational stop codon (in exon 2) was mutated to allow in-frame fusion with the fluorescent marker and additional mAID and TEV (2x) tags.

Source data are available online for this figure.

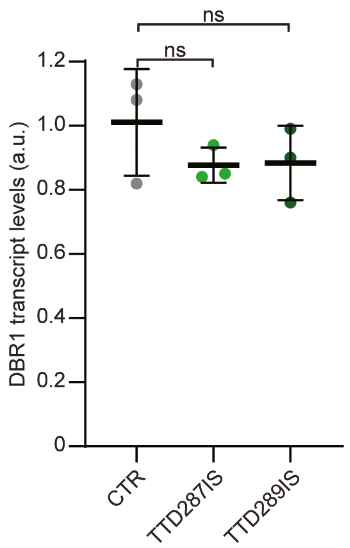

Figure EV4. Analysis of *DBR1* transcript levels in *MPLKIP*-deficient fibroblasts.

Relative DBR1 transcript levels were assessed by qRT-PCR in MPLKIP-deficient primary fibroblasts (TTD287IS and TTD298IS) and control fibroblasts (CTR). Total DBR1 transcript levels were first normalized to the levels of TUBG2 mRNA and then expressed as percentages of the corresponding value in the control fibroblasts (n = 3 biological replicates).

Data information: Data are represented as mean ± SD, ordinary one-way ANOVA; ns, not significant.
Source data are available online for this figure.

Figure EV5. RNA-seq analysis of MPLKIP-deficient TTD HSEs.

A, B Differential gene expression when limiting the analysis only to protein-coding genes with at least two exons. Violin plots of the gene's longest transcript in bp (A) the gene's transcript with the most exons (B) in all significantly down-regulated (red), up-regulated (green), and non-significant (ns, grey) genes (n = 3 biological replicates).

C Number of significant altered splice events detected by rMATS across all gene types when exploring novel un-annotated splicing sites. Alternative 3′ and 5′ Splice Sites (A3SS, A5SS), Mutually eXclusive Exons (MXE), Retained Introns (RI), and Skipped Exons (SE).

D Overview of identified altered splicing events by rMATS between *MPLKIP*-deficient samples (MPLKIP) and control, based on annotated splicing sites (novel Splicing Sites = no) or also including novel splicing sites (novel Splicing Sites = yes). This analysis includes alternative 3′ splice sites (A3SSs), alternative 5′ splice sites (A5SSs), mutually exclusive exons (MXEs), intron retentions (RI), and skipped exons (SEs).

Data information: (A, B). The boxplot boxes and mark denote the first, second, and third quartiles. The boxplot whiskers extend no further than 1.5 * the group's inter-quartile range. The grey dashed line denotes the median of the non-significant group. The dark lines in the violin plots denote the median of the kernel density estimates for each group. The statistical significance of the differences in transcript length and number of exons was obtained with a two-sided Mann–Whitney test.
*P < 0.05; **P < 0.01; *****P < 0.00001; ns, not significant.
Source data are available online for this figure.

© 2023 The Authors

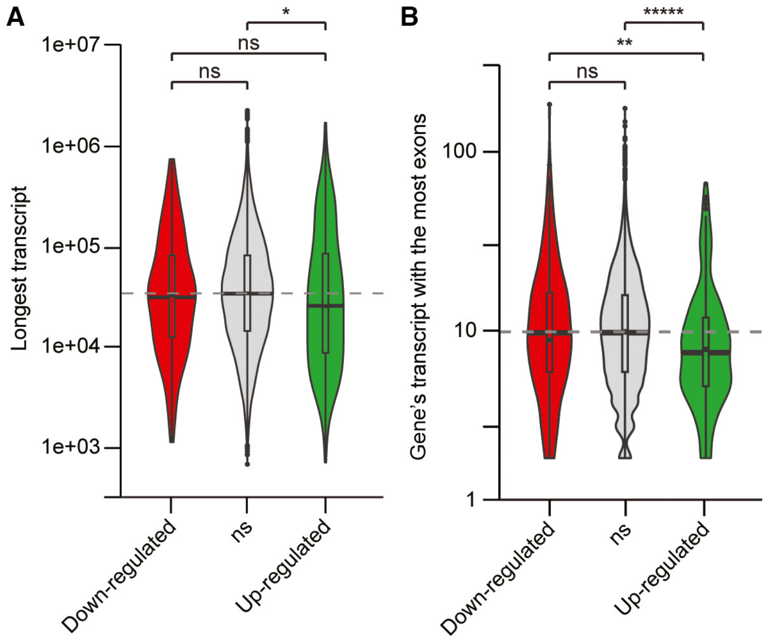

C

Event type	Splice events	Annotated splice events				Annotated and novel splice events			
		Total events	Significant events	Total events	Significant events (%)	Total events	Significant events	Total events	Significant events (%)
A3SS<br>A3SS	> MPLKIP<br>< MPLKIP	4,129<br>3,646	37<br>31	68	0.87%	28,708<br>21,724	102<br>89	191	0.38%
A5SS<br>A5SS	> MPLKIP<br>< MPLKIP	2,803<br>2,340	31<br>31	62	1.21%	22,238<br>17,251	91<br>71	162	0.41%
MXE<br>MXE	> MPLKIP<br>< MPLKIP	3,837<br>2,924	153<br>67	220	3.25%	29,764<br>22,318	563<br>824	1,387	2.66%
RI<br>RI	> MPLKIP<br>< MPLKIP	3,238<br>2,413	86<br>45	131	2.32%	10,969<br>7,757	169<br>91	260	1.39%
SE<br>SE	> MPLKIP<br>< MPLKIP	29,489<br>24,581	331<br>272	603	1.12%	51,040<br>42,955	588<br>493	1,081	1.15%

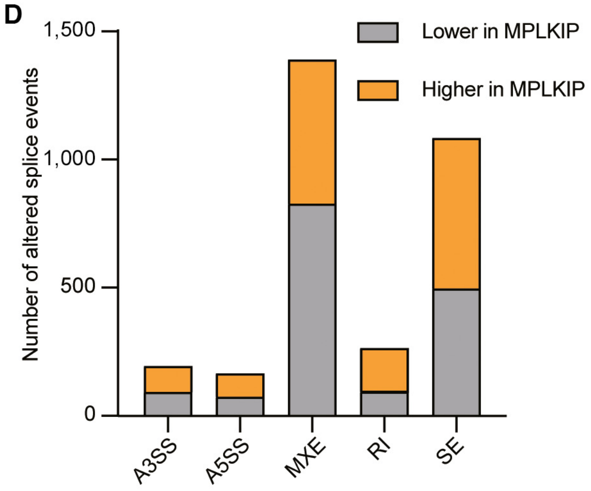

Figure EV5.

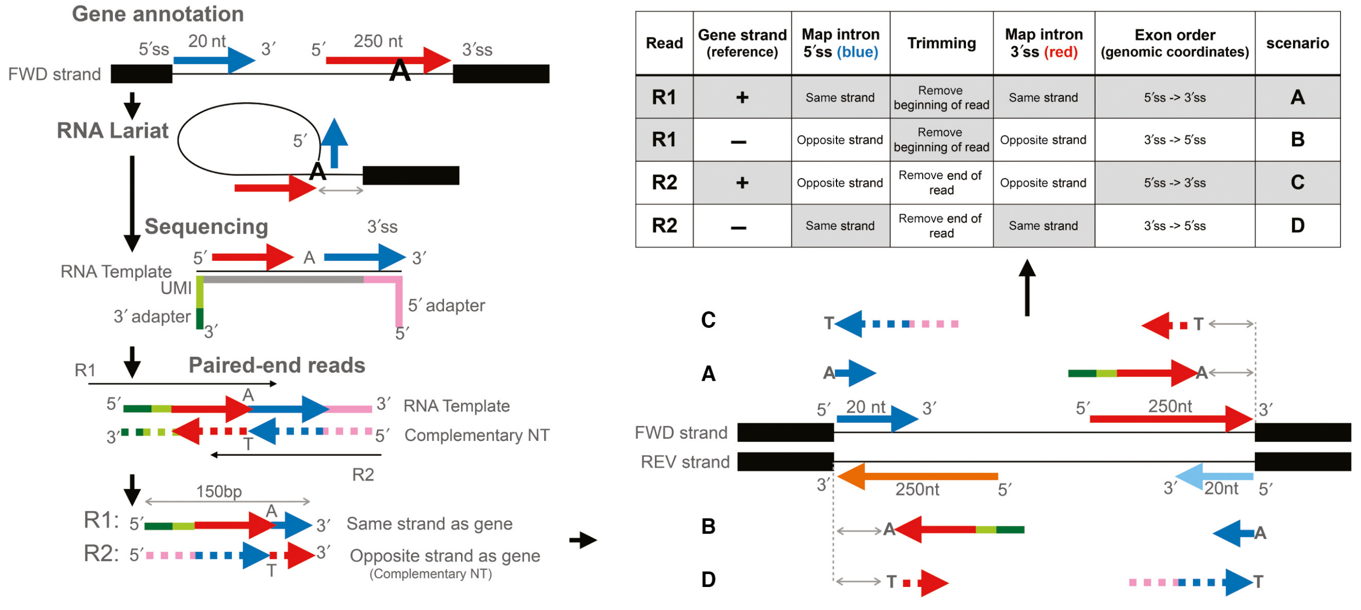

Read	Gene strand (reference)	Map intron 5′ss (blue)	Trimming	Map intron 3′ss (red)	Exon order (genomic coordinates)	scenario
R1	+	Same strand	Remove beginning of read	Same strand	5′ss -> 3′ss	A
R1	–	Opposite strand	Remove beginning of read	Opposite strand	3′ss -> 5′ss	B
R2	+	Opposite strand	Remove end of read	Opposite strand	5′ss -> 3′ss	C
R2	–	Same strand	Remove end of read	Same strand	3′ss -> 5′ss	D

Figure EV6. Schematic of the lariat detection process.

Schematic of the lariat detection process. Lariat loops originate during splicing from the intronic regions adjacent to the 5′ (blue) and 3′ (red) splicing sites. The spliced intron creates a loop at a branchpoint location (chiefly an adenine). Lariats can be identified by RNAseq when sequencing reads overlap this branching point. This creates a read hybrid between the intronic region adjacent to the 3′ splicing site upstream and the one adjacent to the 5′ splicing site. Paired-end sequencing might give rise to 150 bp-long reads overlapping the branchpoint region that are in the complementary strand (dashed arrows) and will map to the opposite strand in the genome. The combination of the gene's annotated strand and the two read pairs gives rise to four different scenarios to computationally map and identify reads originating from a lariat.

Source data are available online for this figure.

 © 2023 The Authors
