# Supplementary figures and images for: Trichothiodystrophy‐associated MPLKIP maintains DBR1 levels for proper lariat debranching and ectodermal differentiation
Source: EMBO Mol Med. 2023 Oct 6;15(11):e17973. doi: 10.15252/emmm.202317973 (PMC10630875; doi:10.15252/emmm.202317973)

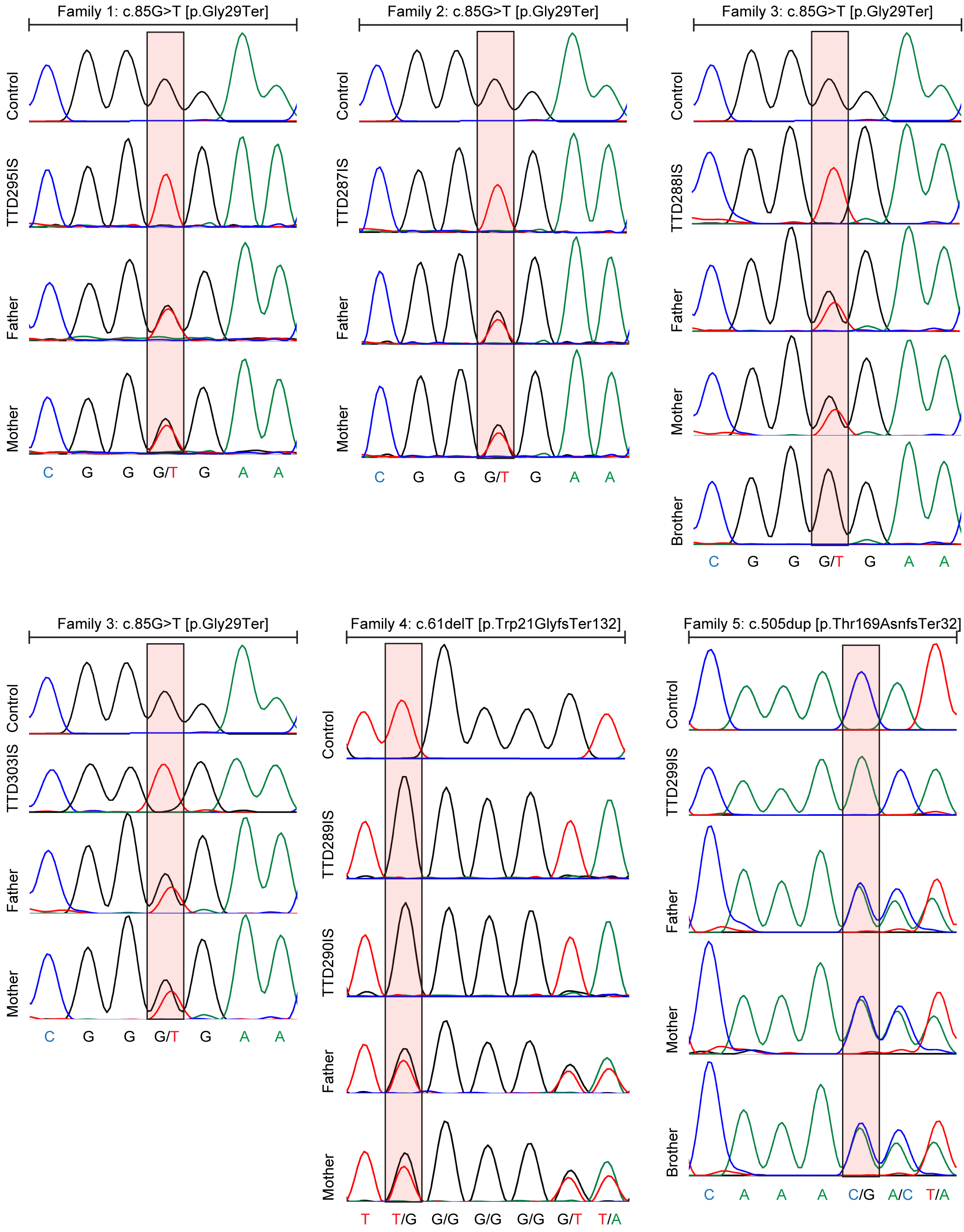

Supplement: Supplementary file 3 — Source Data for Expanded View [file EMMM-15-e17973-s005.zip › EMM-2023-17973_SourceDataForFigureEV1/EMM-2023-17973_SourceDataForFigureEV1.tif]

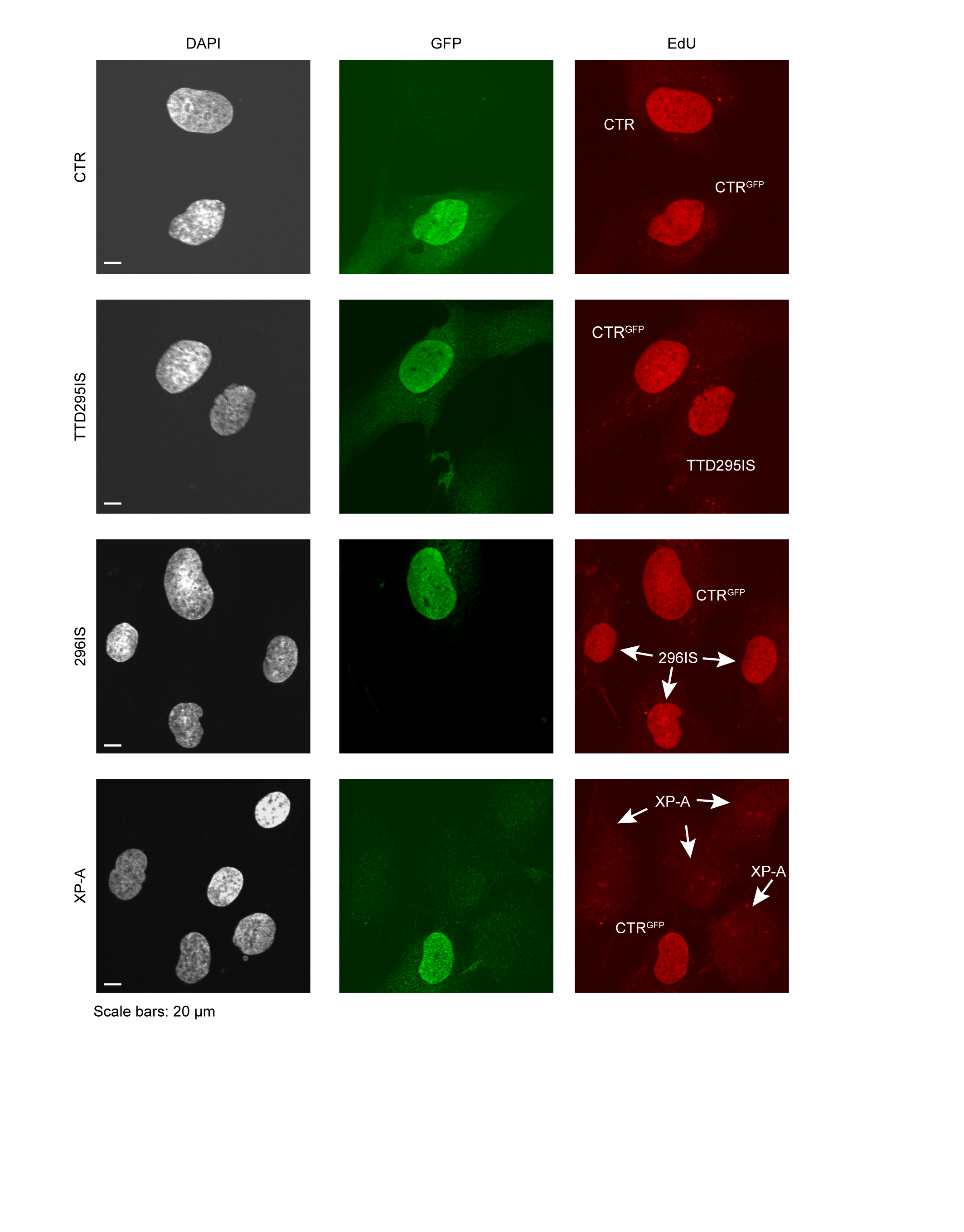

Supplement: Supplementary file 3 — Source Data for Expanded View [file EMMM-15-e17973-s005.zip › EMM-2023-17973_SourceDataForFigureEV2/EMM-2023-17973_SourceDataForFigureEV2A.tif]

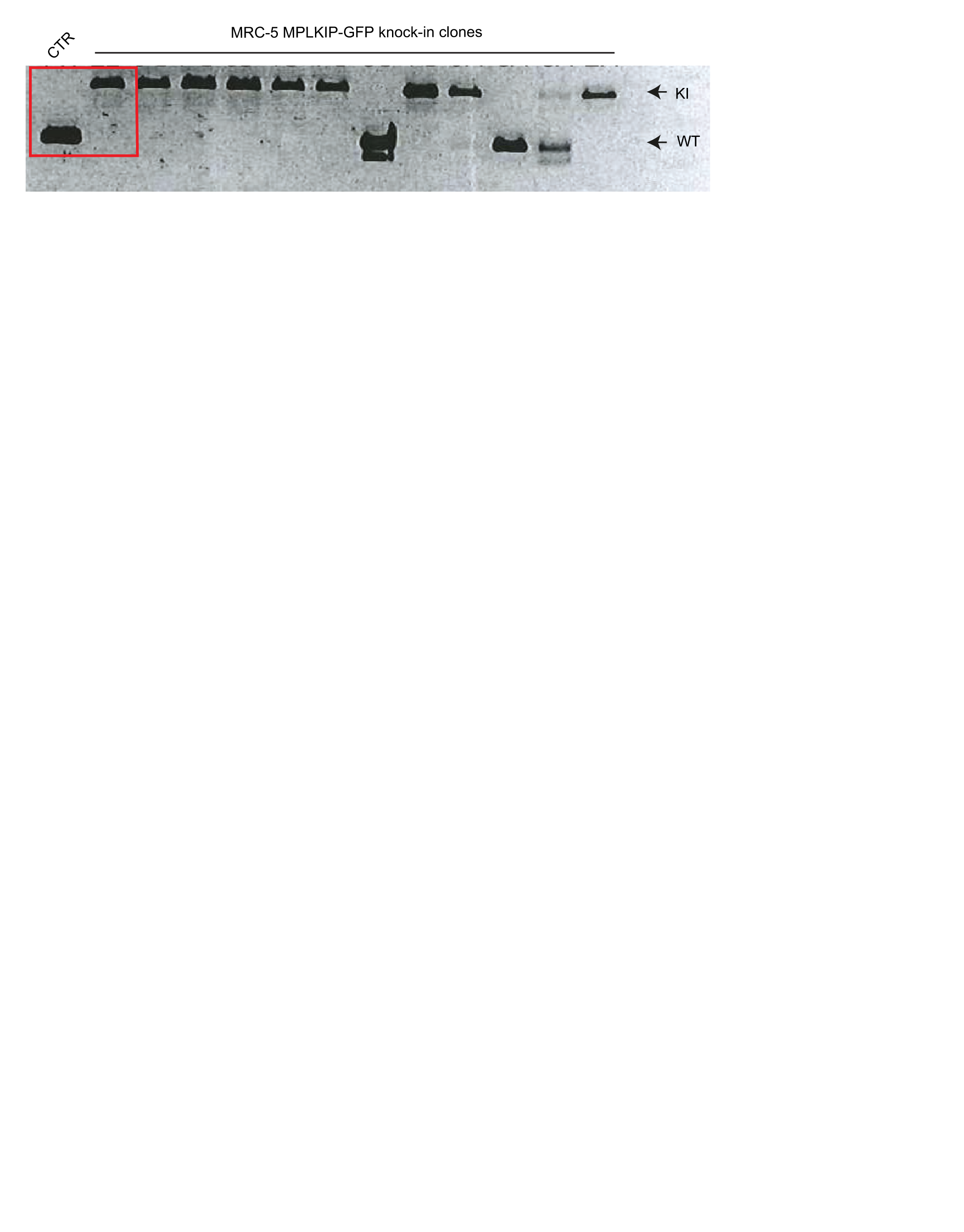

Supplement: Supplementary file 3 — Source Data for Expanded View [file EMMM-15-e17973-s005.zip › EMM-2023-17973_SourceDataForFigureEV3/EMM-2023-17973_SourceDataForFigureEV3B.tif]

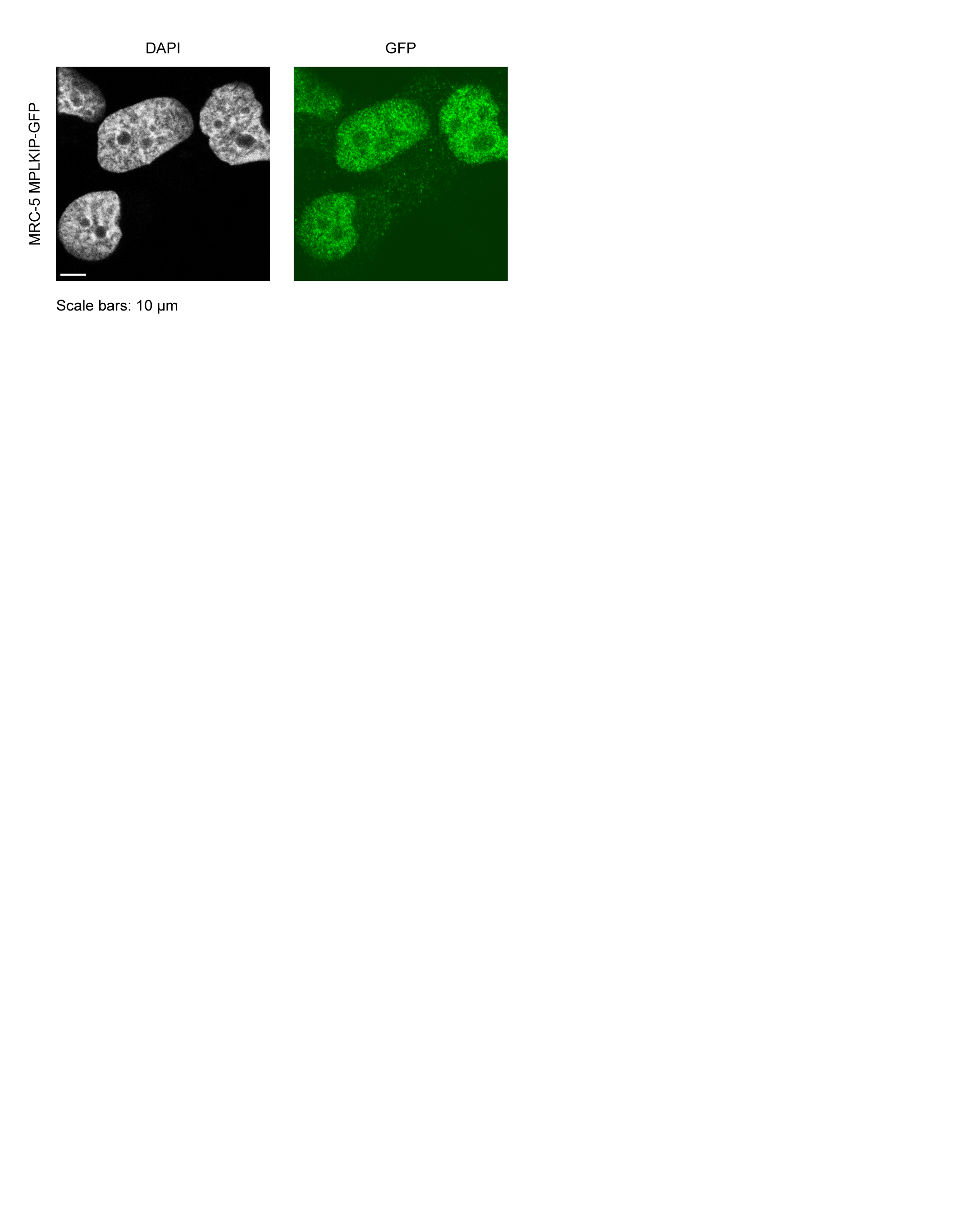

Supplement: Supplementary file 3 — Source Data for Expanded View [file EMMM-15-e17973-s005.zip › EMM-2023-17973_SourceDataForFigureEV3/EMM-2023-17973_SourceDataForFigureEV3C.tif]

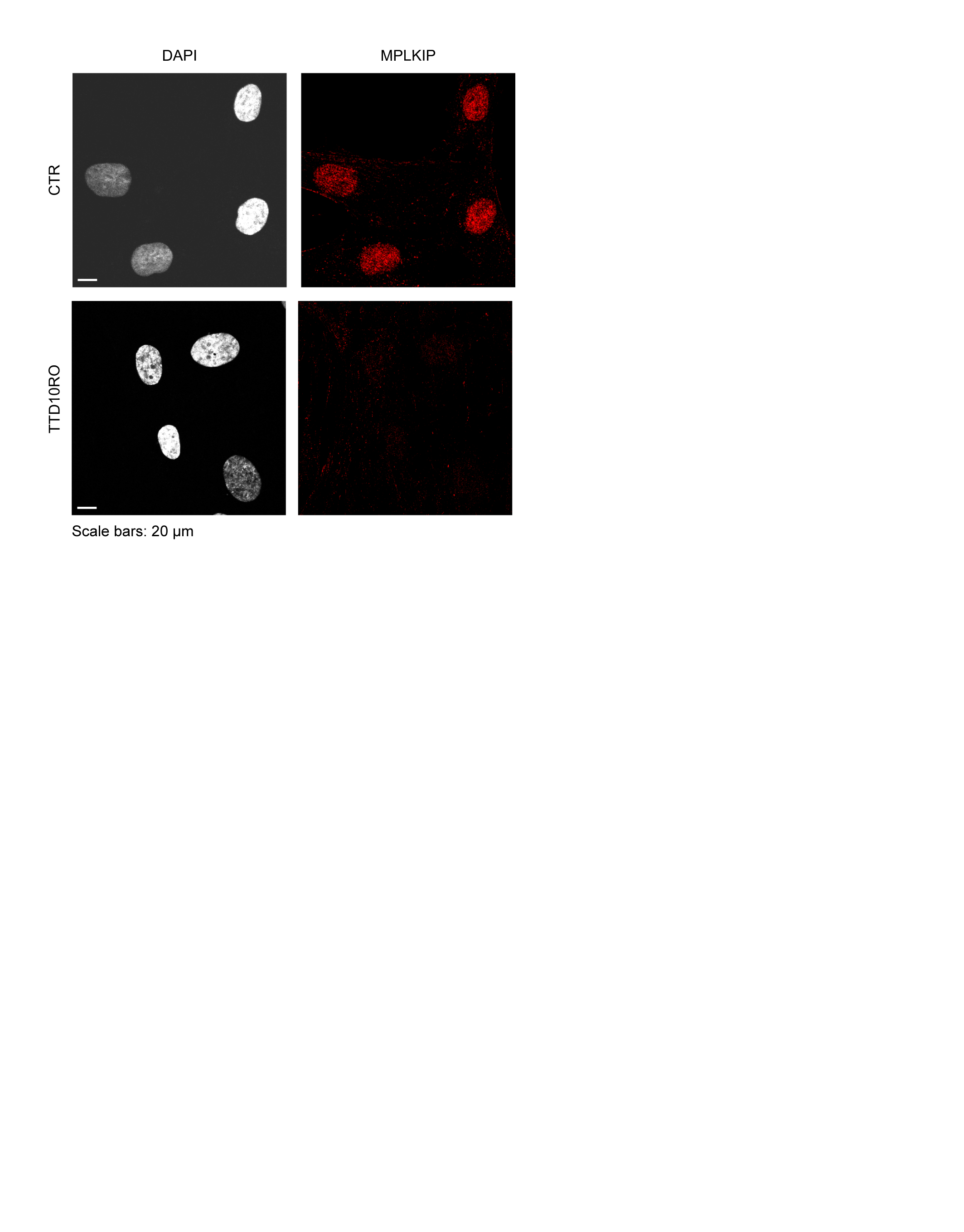

Supplement: Supplementary file 3 — Source Data for Expanded View [file EMMM-15-e17973-s005.zip › EMM-2023-17973_SourceDataForFigureEV3/EMM-2023-17973_SourceDataForFigureEV3D.tif]

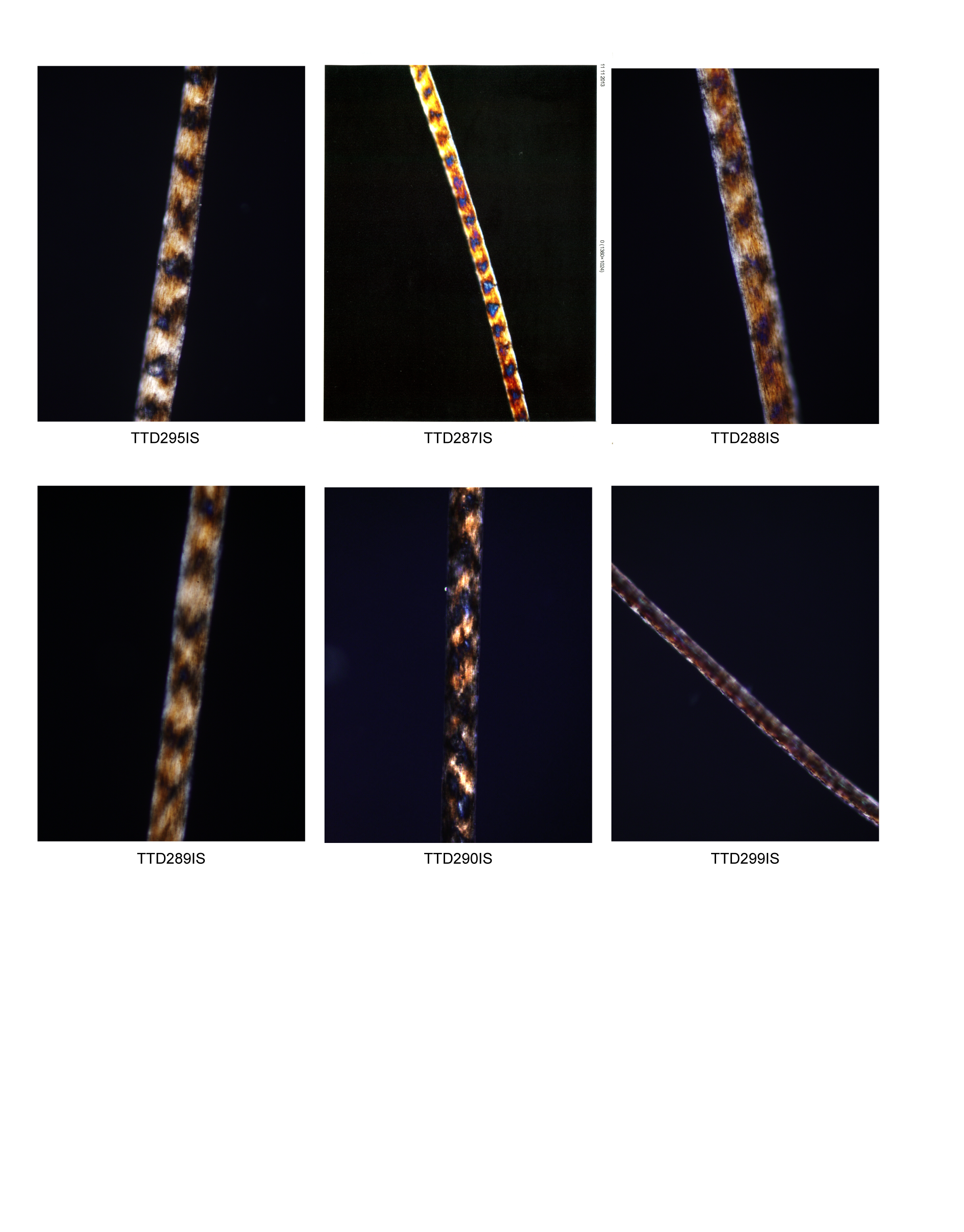

Supplement: Supplementary file 5 — Source Data for Figure 2 [file EMMM-15-e17973-s012.zip › EMM-2023-17973_SourceDataForFigure2A.tif]

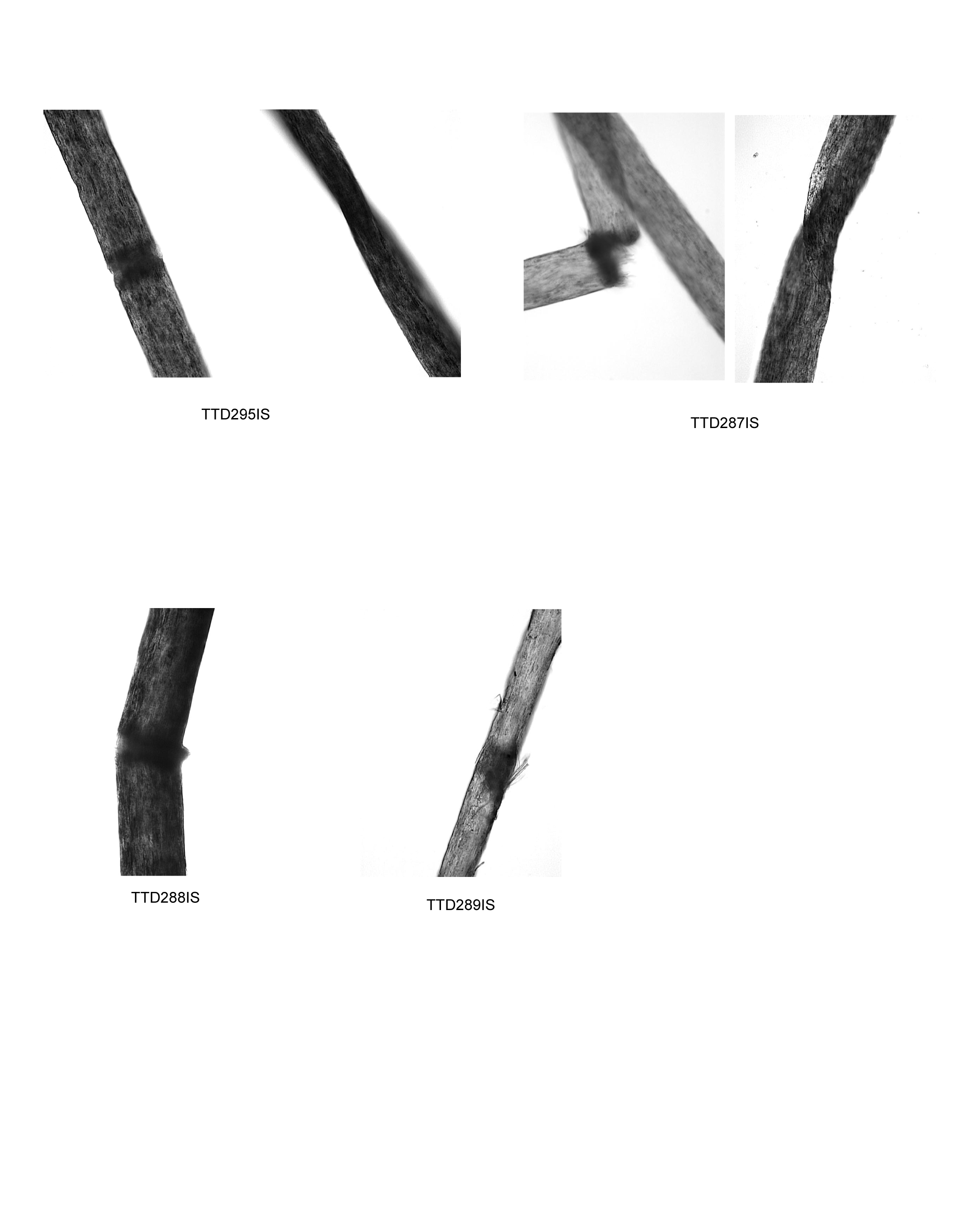

Supplement: Supplementary file 5 — Source Data for Figure 2 [file EMMM-15-e17973-s012.zip › EMM-2023-17973_SourceDataForFigure2B.tif]

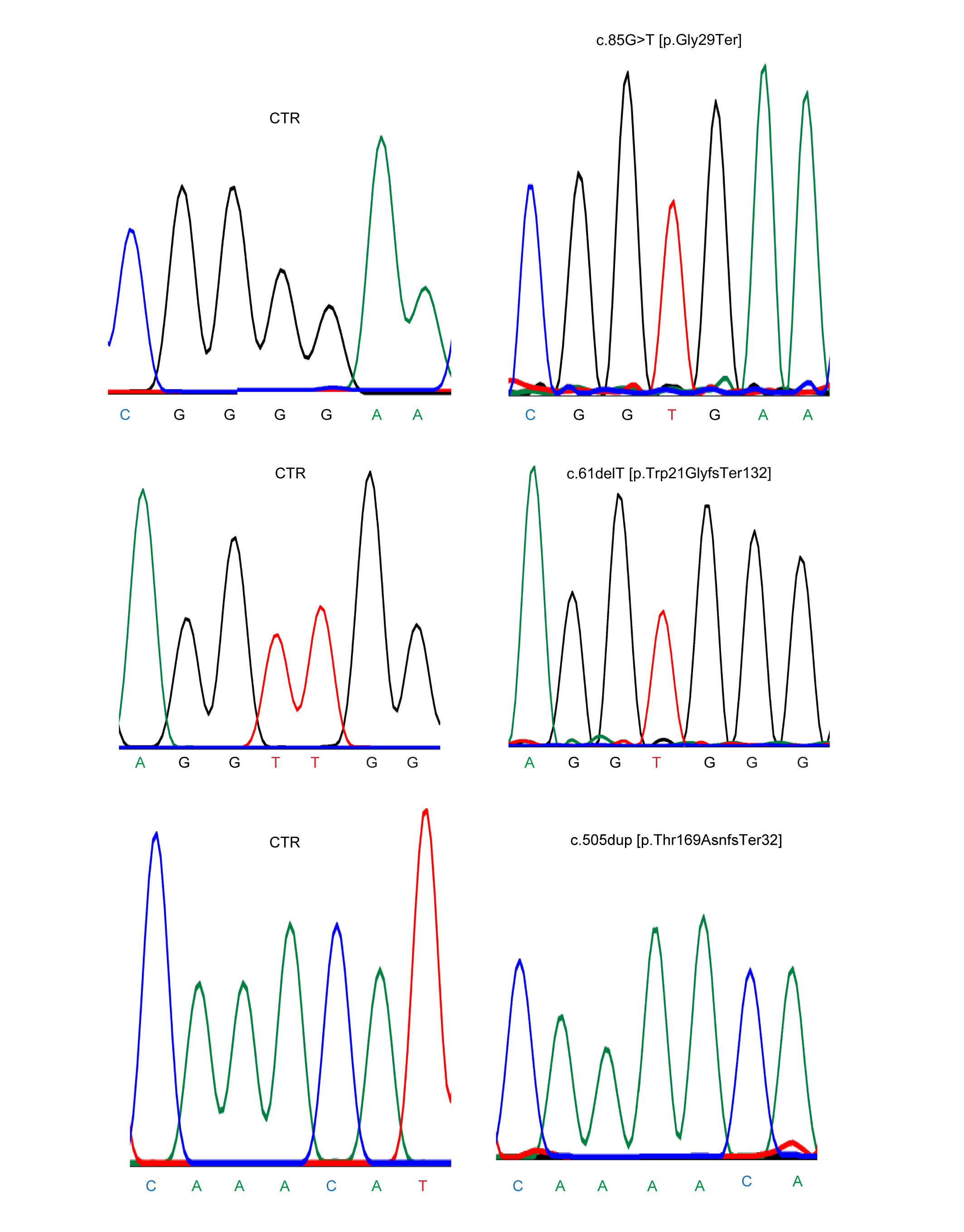

Supplement: Supplementary file 5 — Source Data for Figure 2 [file EMMM-15-e17973-s012.zip › EMM-2023-17973_SourceDataForFigure2C.tif]

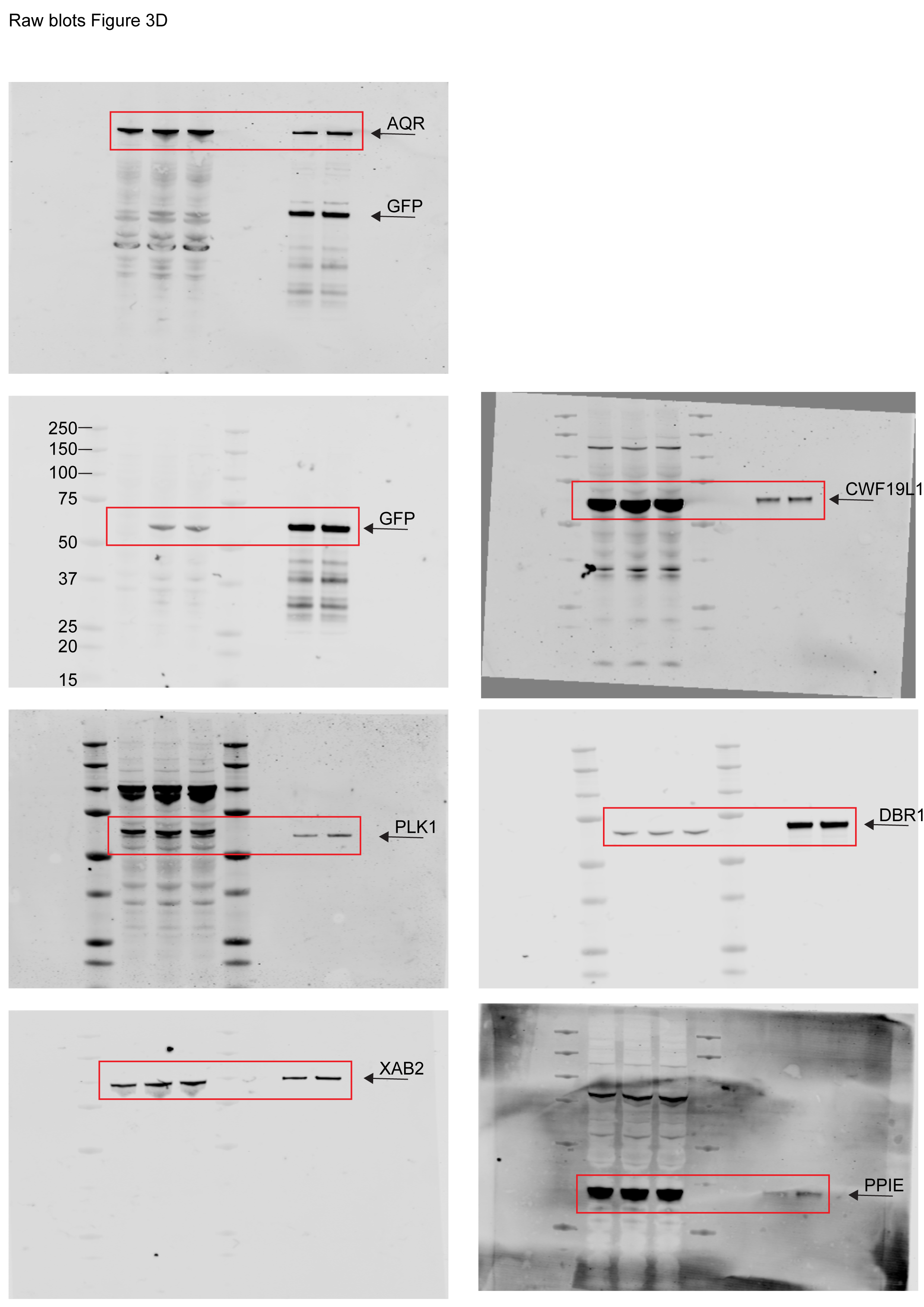

Supplement: Supplementary file 6 — Source Data for Figure 3 [file EMMM-15-e17973-s006.zip › EMM-2023-17973_SourceDataForFigure3D.tif]

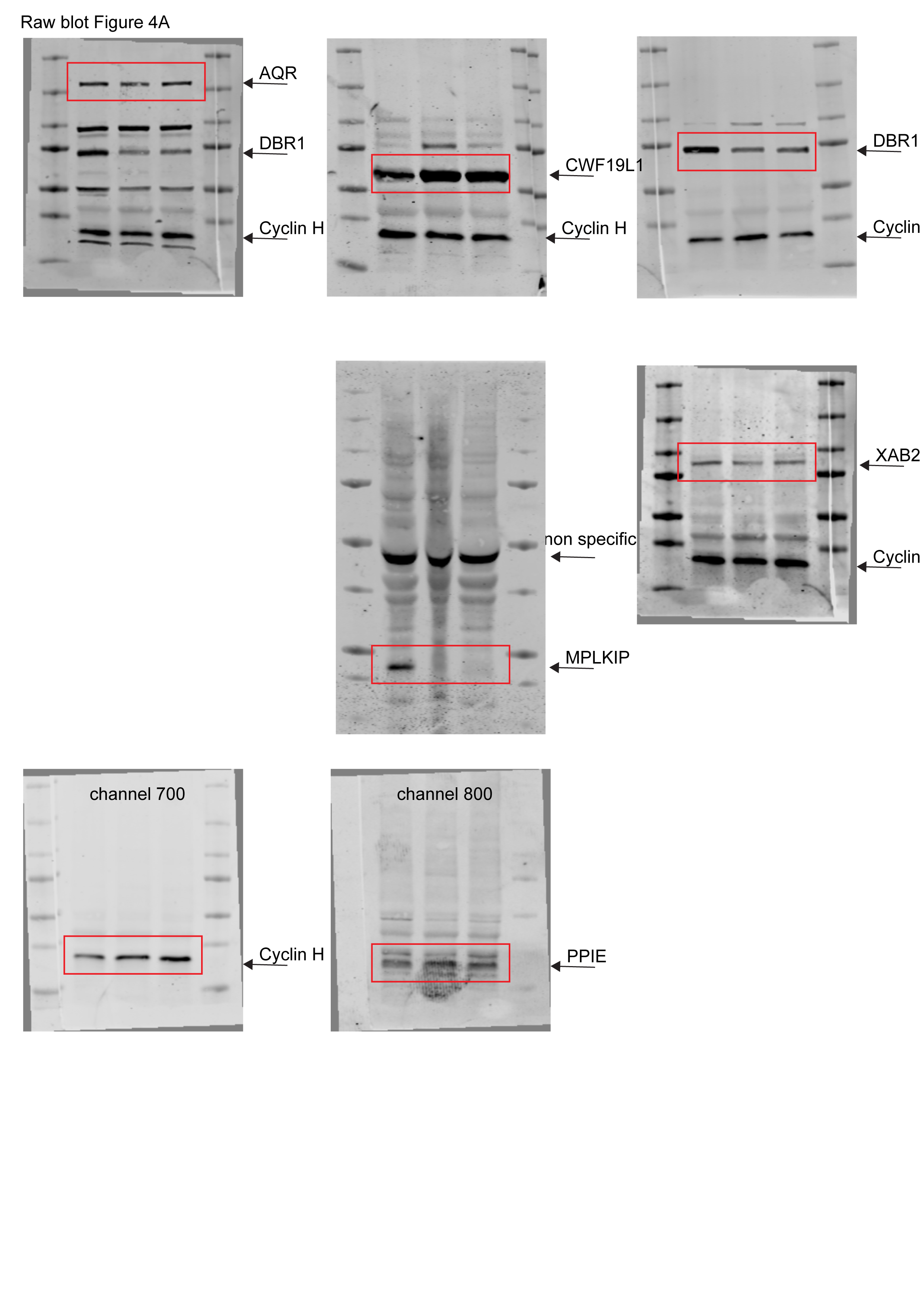

Supplement: Supplementary file 7 — Source Data for Figure 4 [file EMMM-15-e17973-s002.zip › EMM-2023-17973_SourceDataForFigure4A.tif]

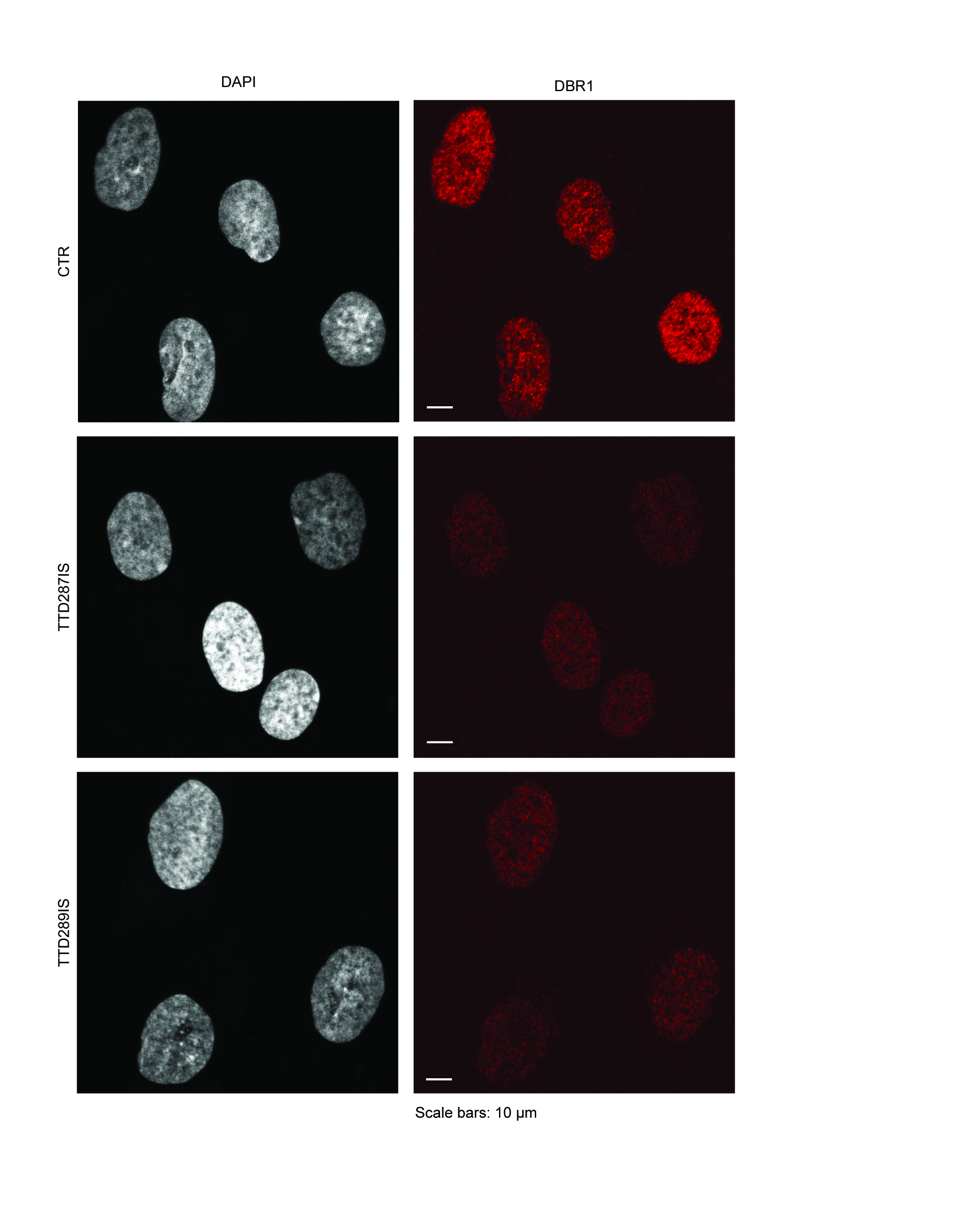

Supplement: Supplementary file 7 — Source Data for Figure 4 [file EMMM-15-e17973-s002.zip › EMM-2023-17973_SourceDataForFigure4C.tif]

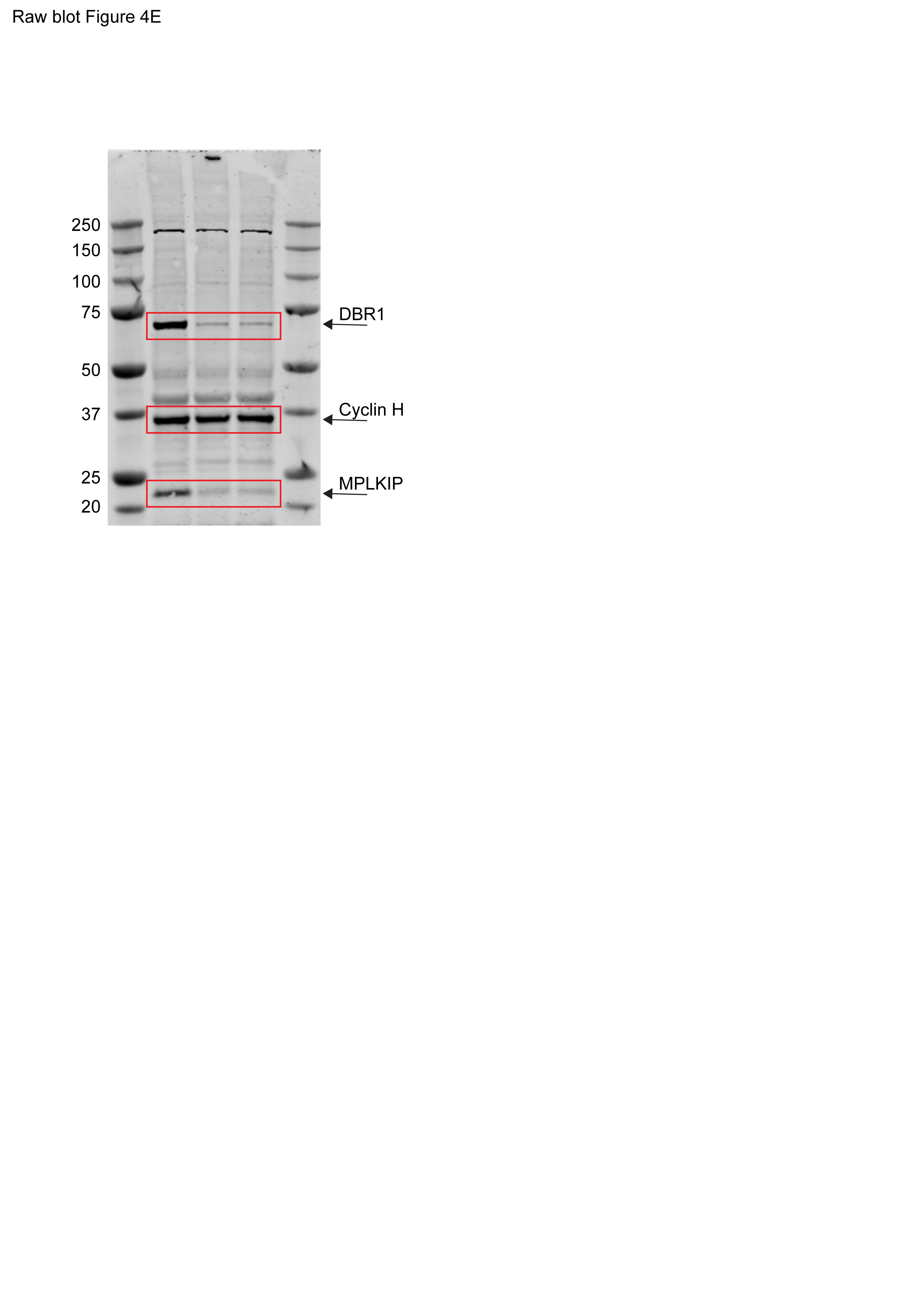

Supplement: Supplementary file 7 — Source Data for Figure 4 [file EMMM-15-e17973-s002.zip › EMM-2023-17973_SourceDataForFigure4E.tif]

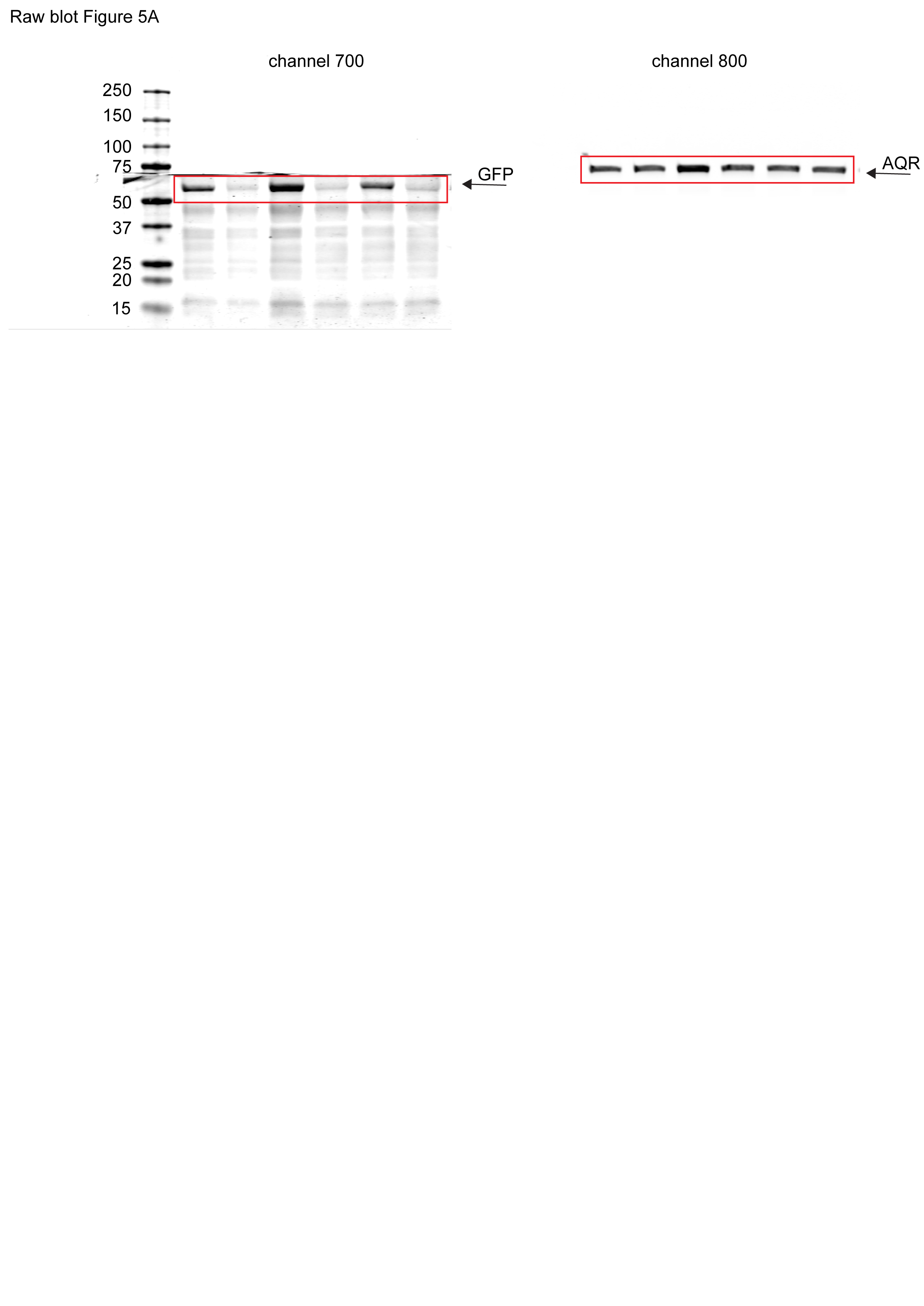

Supplement: Supplementary file 8 — Source Data for Figure 5 [file EMMM-15-e17973-s007.zip › EMM-2023-17973_SourceDataForFigure5A.tif]

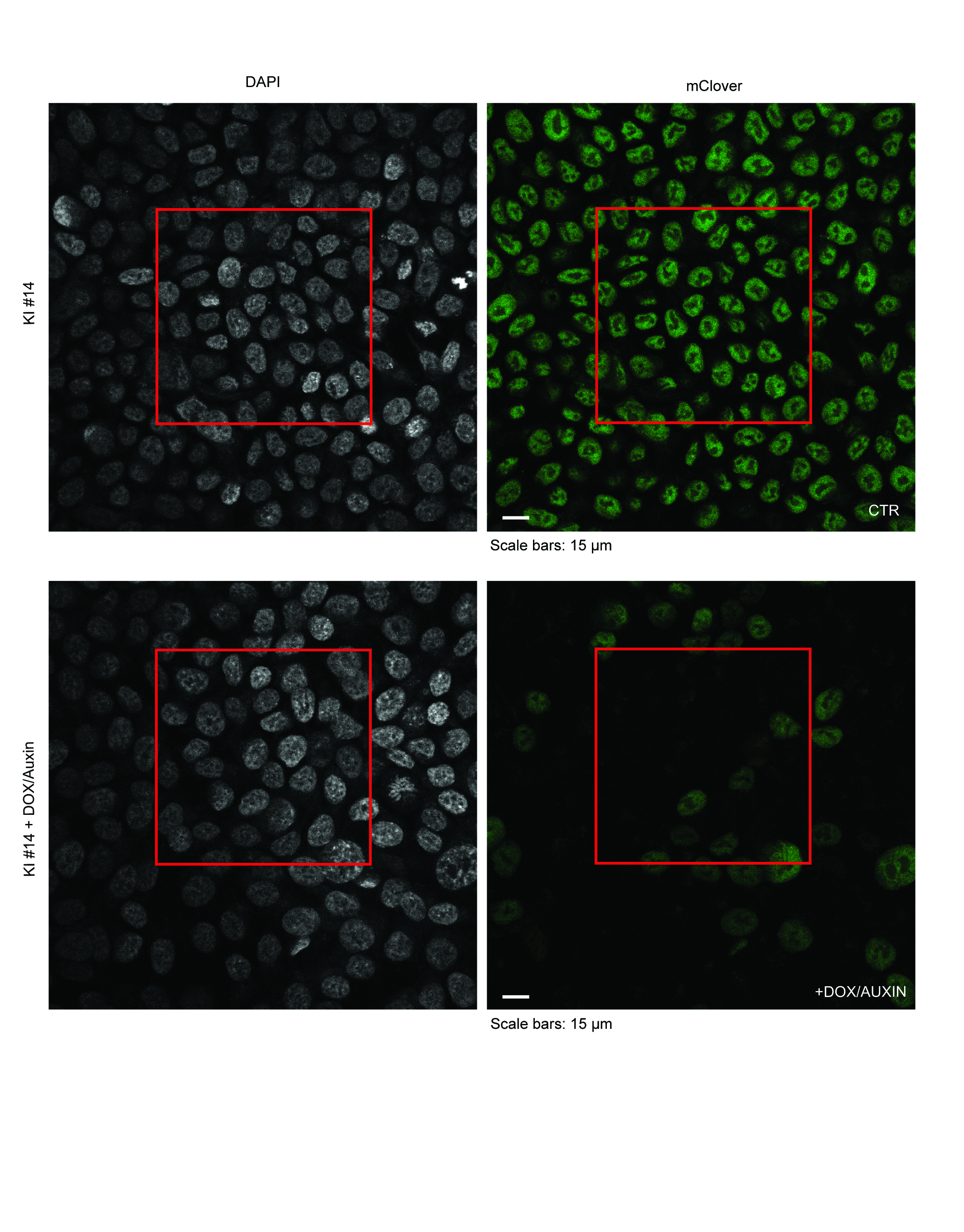

Supplement: Supplementary file 8 — Source Data for Figure 5 [file EMMM-15-e17973-s007.zip › EMM-2023-17973_SourceDataForFigure5B.tif]

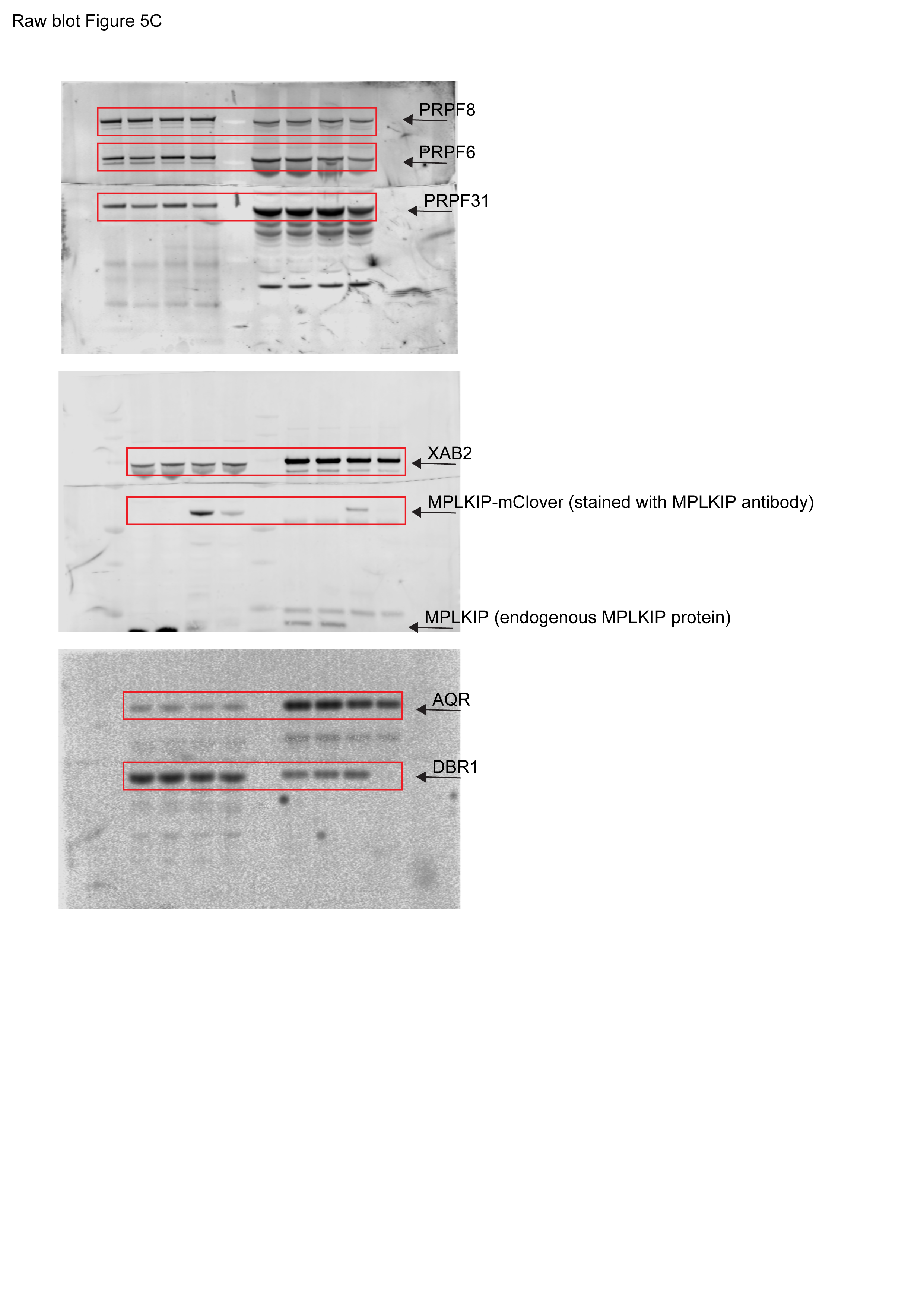

Supplement: Supplementary file 8 — Source Data for Figure 5 [file EMMM-15-e17973-s007.zip › EMM-2023-17973_SourceDataForFigure5C.tif]

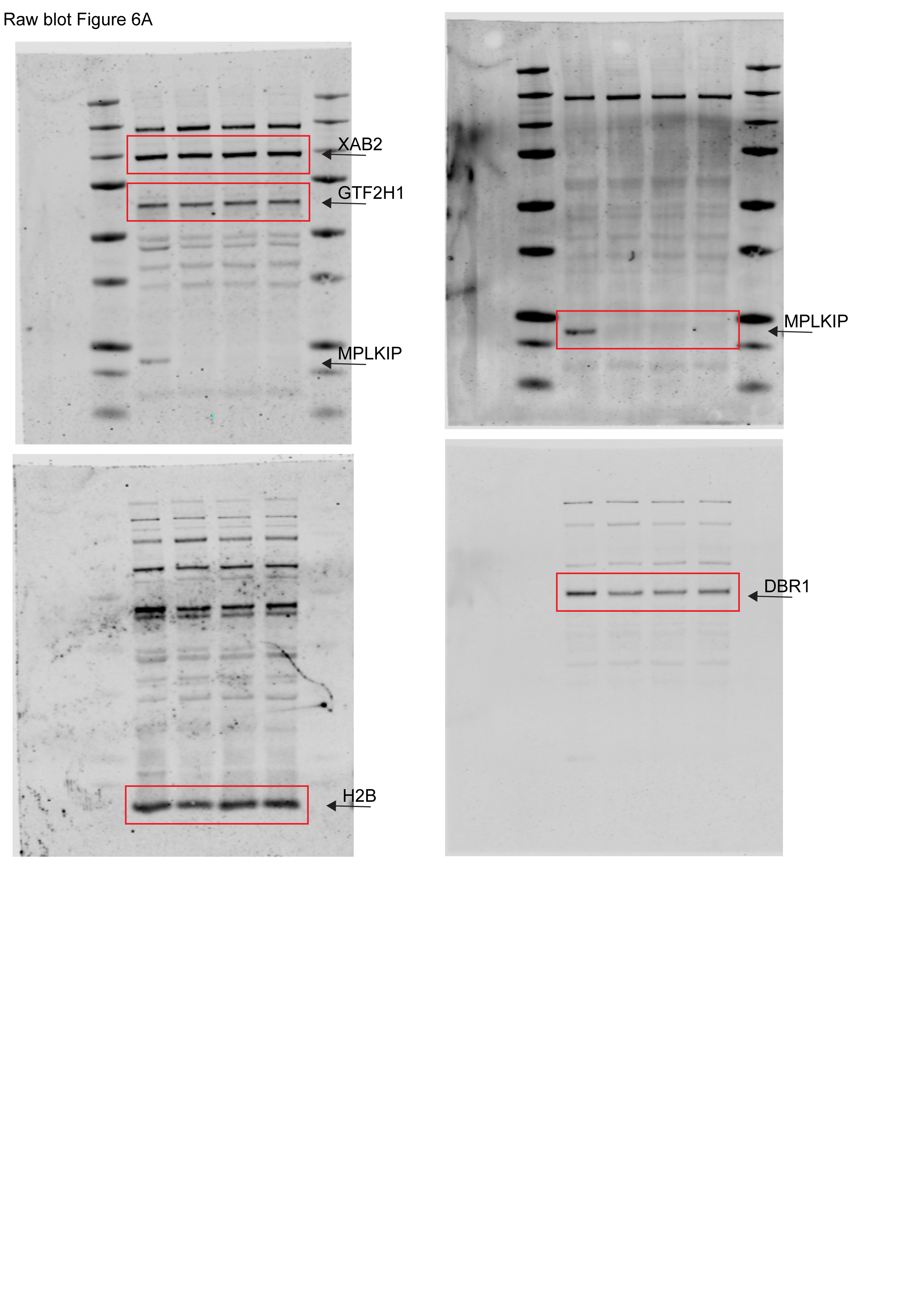

Supplement: Supplementary file 9 — Source Data for Figure 6 [file EMMM-15-e17973-s008.zip › EMM-2023-17973_SourceDataForFigure6A.tif]

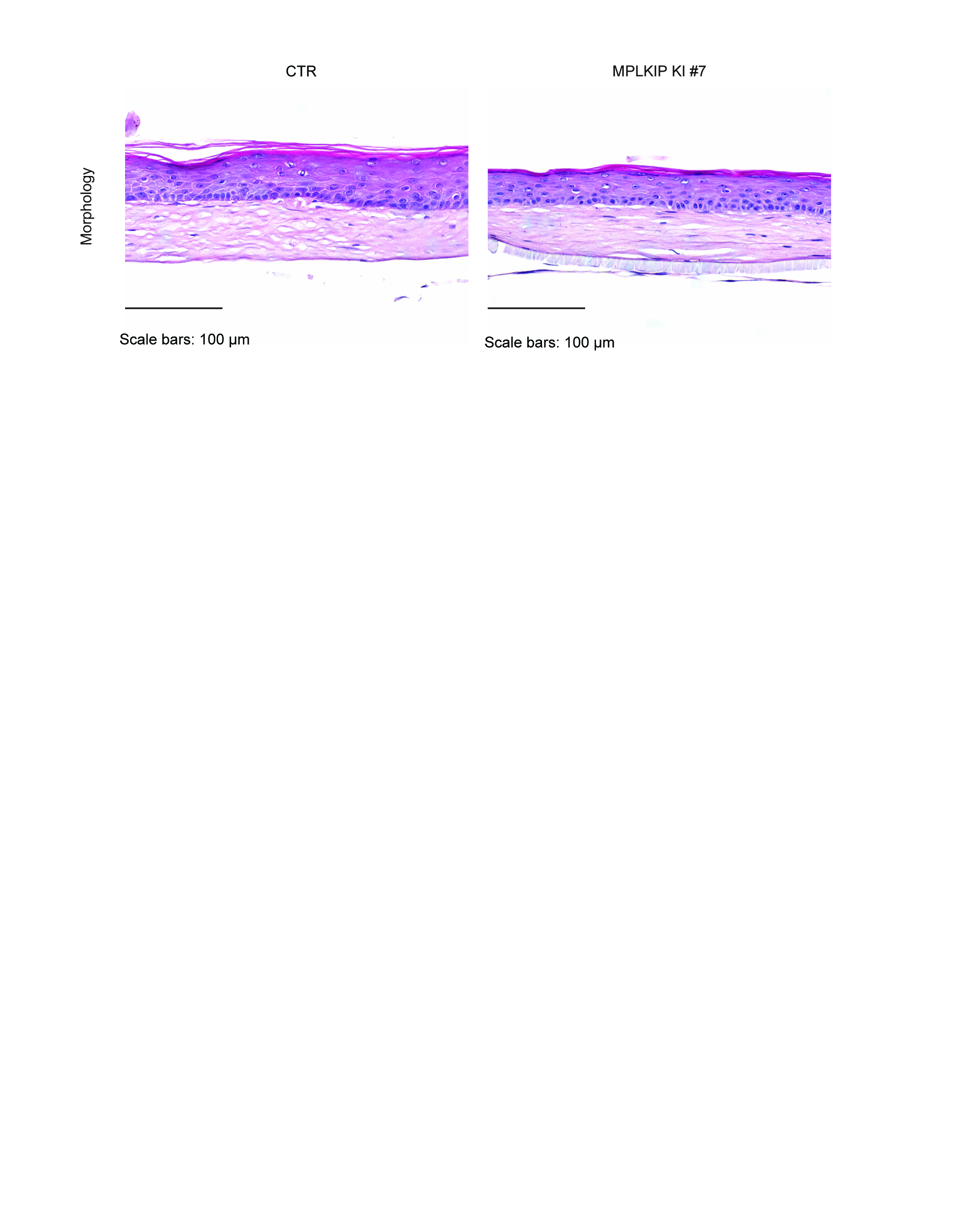

Supplement: Supplementary file 10 — Source Data for Figure 7 [file EMMM-15-e17973-s003.zip › EMM-2023-17973_SourceDataForFigure7A.tif]

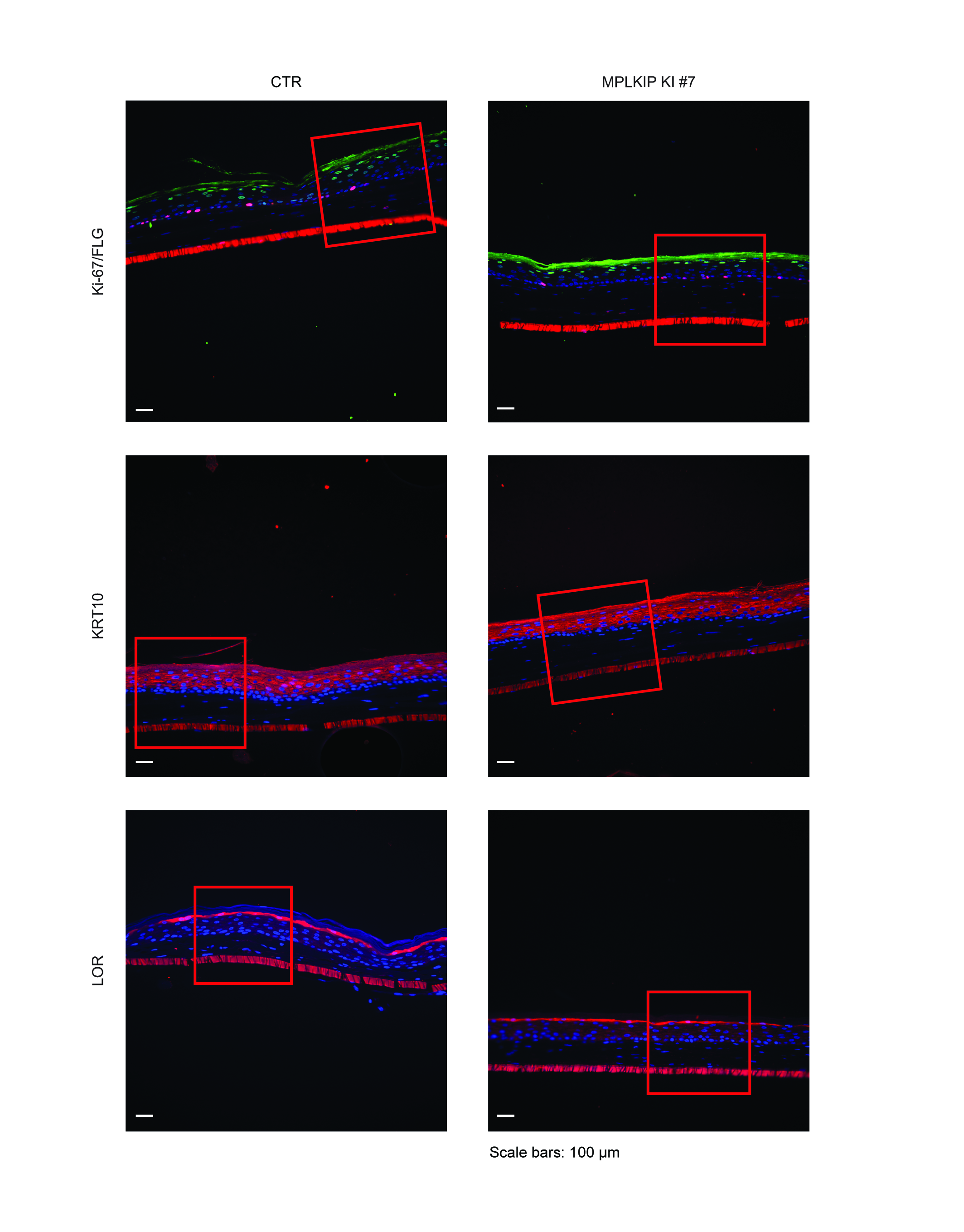

Supplement: Supplementary file 10 — Source Data for Figure 7 [file EMMM-15-e17973-s003.zip › EMM-2023-17973_SourceDataForFigure7D.tif]

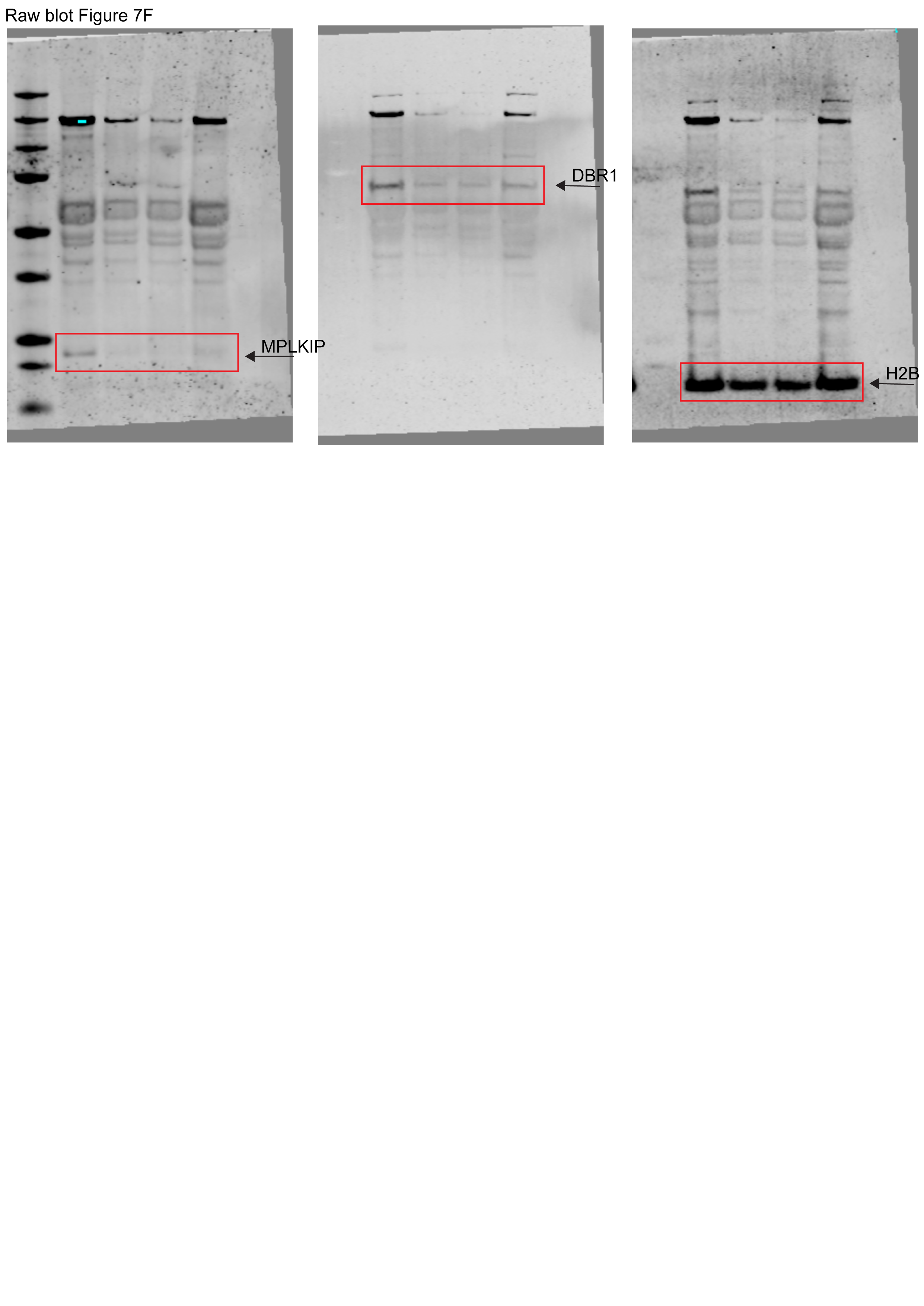

Supplement: Supplementary file 10 — Source Data for Figure 7 [file EMMM-15-e17973-s003.zip › EMM-2023-17973_SourceDataForFigure7F.tif]
