# Supplementary material for: Trichothiodystrophy‐associated MPLKIP maintains DBR1 levels for proper lariat debranching and ectodermal differentiation
Source: EMBO Mol Med. 2023 Oct 6;15(11):e17973. doi: 10.15252/emmm.202317973 (PMC10630875; doi:10.15252/emmm.202317973)
Supplement: Supplementary file 13 [file EMMM-15-e17973-s001.pdf]

EMBO Molecular Medicine

Trichothiodystrophy-associated MPLKIP maintains DBR1 levels for proper lariat debranching and ectodermal differentiation

Wim Vermeulen, Arjan Theil, Alex Pines, Tuğba Kalayci, Umut Altunoglu, Hülya kayserili, Nesimi Büyükbabani, Anja Raams, Marion Rietveld, Abdoel El Ghalbzouri, Sabine Tanis, Klaas Mulder, Joris Pothof, Jeroen Demmers, Hannes Lans, José Heredia-Genestar, Birsen Karaman, Sriram Sridharan, Zehra Uyguner, and Jan H.J. Hoeijmakers
DOI: 10.15252/emmm.202317973

Corresponding authors: Wim Vermeulen (W.Vermeulen@erasmusmc.nl)

Review Timeline:

Submission Date:	10th May 23
Editorial Decision:	31st May 23
Revision Received:	23rd Aug 23
Editorial Decision:	8th Sep 23
Revision Received:	15th Sep 23
Accepted:	18th Sep 23

Editor: Zeljko Durdevic

Transaction Report:

(Note: With the exception of the correction of typographical or spelling errors that could be a source of ambiguity, letters and reports are not edited. Depending on transfer agreements, referee reports obtained elsewhere may or may not be included in this compilation. Referee reports are anonymous unless the Referee chooses to sign their reports.)

31st May 2023

Dear Prof. Vermeulen,

Thank you for the submission of your manuscript to EMBO Molecular Medicine. We have now received feedback from the two reviewers who agreed to evaluate your manuscript. While referee #1 is overall supportive of the study raising important but minor concerns, referee #2 recognizes the interest of the study but also raises serious concerns that should be addressed in a major revision. Addressing referee #2 points 1-3 experimentally will be essential for further consideration of the manuscript. If you would like to discuss further the points raised by the referees, I am available to do so via email or video. Let me know if you are interested in this option.

Further consideration of a revision that addresses reviewers' concerns in full will entail a second round of review. EMBO Molecular Medicine encourages a single round of revision only and therefore, acceptance or rejection of the manuscript will depend on the completeness of your responses included in the next, final version of the manuscript. For this reason, and to save you from any frustrations in the end, I would strongly advise against returning an incomplete revision.

I look forward to receiving your revised manuscript.

Yours sincerely,

Zeljko Durdevic

Zeljko Durdevic
Editor
EMBO Molecular Medicine

*****

When submitting your revised manuscript, please carefully review the instructions that follow below. We perform an initial quality control of all revised manuscripts before re-review; failure to include requested items will delay the evaluation of your revision.

We require:

1) A .docx formatted version of the manuscript text (including legends for main figures, EV figures and tables). Please make sure that the changes are highlighted to be clearly visible.

2) Individual production quality figure files as .eps, .tif, .jpg (one file per figure). For guidance, download the 'Figure Guide PDF': (https://www.embopress.org/page/journal/17574684/authorguide#figureformat).

3) A .docx formatted letter INCLUDING the reviewers' reports and your detailed point-by-point responses to their comments. As part of the EMBO Press transparent editorial process, the point-by-point response is part of the Review Process File (RPF), which will be published alongside your paper.

4) A complete author checklist, which you can download from our author guidelines (https://www.embopress.org/page/journal/17574684/authorguide#submissionofrevisions). Please insert information in the checklist that is also reflected in the manuscript. The completed author checklist will also be part of the RPF.

5) Please note that all corresponding authors are required to supply an ORCID ID for their name upon submission of a revised manuscript.

6) It is mandatory to include a 'Data Availability' section after the Materials and Methods. Before submitting your revision, primary datasets produced in this study need to be deposited in an appropriate public database, and the accession numbers and database listed under 'Data Availability'. Please remember to provide a reviewer password if the datasets are not yet public (see

https://www.embopress.org/page/journal/17574684/authorguide#dataavailability).

In case you have no data that requires deposition in a public database, please state so in this section. Note that the Data Availability Section is restricted to new primary data that are part of this study.

7) For data quantification: please specify the name of the statistical test used to generate error bars and P values, the number (n) of independent experiments (specify technical or biological replicates) underlying each data point and the test used to calculate p-values in each figure legend. The figure legends should contain a basic description of n, P and the test applied. Graphs must include a description of the bars and the error bars (s.d., s.e.m.). See also 'Figure Legend' guidelines: https://www.embopress.org/page/journal/17574684/authorguide#figureformat

8) At EMBO Press we ask authors to provide source data for the main manuscript figures. Our source data coordinator will contact you to discuss which figure panels we would need source data for and will also provide you with helpful tips on how to upload and organize the files.

9) Our journal encourages inclusion of *data citations in the reference list* to directly cite datasets that were re-used and obtained from public databases. Data citations in the article text are distinct from normal bibliographical citations and should directly link to the database records from which the data can be accessed. In the main text, data citations are formatted as follows: "Data ref: Smith et al, 2001" or "Data ref: NCBI Sequence Read Archive PRJNA342805, 2017". In the Reference list, data citations must be labeled with "[DATASET]". A data reference must provide the database name, accession number/identifiers and a resolvable link to the landing page from which the data can be accessed at the end of the reference. Further instructions are available at .

10) We replaced Supplementary Information with Expanded View (EV) Figures and Tables that are collapsible/expandable online. A maximum of 5 EV Figures can be typeset. EV Figures should be cited as 'Figure EV1, Figure EV2" etc... in the text and their respective legends should be included in the main text after the legends of regular figures.

- For the figures that you do NOT wish to display as Expanded View figures, they should be bundled together with their legends in a single PDF file called *Appendix*, which should start with a short Table of Content. Appendix figures should be referred to in the main text as: "Appendix Figure S1, Appendix Figure S2" etc.

- Additional Tables/Datasets should be labeled and referred to as Table EV1, Dataset EV1, etc. Legends have to be provided in a separate tab in case of .xls files. Alternatively, the legend can be supplied as a separate text file (README) and zipped together with the Table/Dataset file.

See detailed instructions here:

.

11) The paper explained: EMBO Molecular Medicine articles are accompanied by a summary of the articles to emphasize the major findings in the paper and their medical implications for the non-specialist reader. Please provide a draft summary of your article highlighting

- the medical issue you are addressing,

- the results obtained and

- their clinical impact.

This may be edited to ensure that readers understand the significance and context of the research. Please refer to any of our published articles for an example.

12) For more information: There is space at the end of each article to list relevant web links for further consultation by our readers. Could you identify some relevant ones and provide such information as well? Some examples are patient associations, relevant databases, OMIM/proteins/genes links, author's websites, etc...

13) Author contributions: You will be asked to provide CRediT (Contributor Role Taxonomy) terms in the submission system. These replace a narrative author contribution section in the manuscript.

14) A Conflict of Interest statement should be provided in the main text.

15) Every published paper now includes a 'Synopsis' to further enhance discoverability. Synopses are displayed on the journal webpage and are freely accessible to all readers. They include a short stand first (maximum of 300 characters, including space)

as well as 2-5 one-sentences bullet points that summarizes the paper. Please write the bullet points to summarize the key NEW findings. They should be designed to be complementary to the abstract - i.e. not repeat the same text. We encourage inclusion of key acronyms and quantitative information (maximum of 30 words / bullet point). Please use the passive voice. Please attach these in a separate file or send them by email, we will incorporate them accordingly.

Please also suggest a striking image or visual abstract to illustrate your article as a PNG file 550 px wide x 300-600 px high.

EMBO Molecular Medicine has a "scooping protection" policy, whereby similar findings that are published by others during review or revision are not a criterion for rejection. Should you decide to submit a revised version, I do ask that you get in touch after three months if you have not completed it, to update us on the status.

Please note: When submitting your revision you will be prompted to enter your funding and payment information. This will allow Wiley to send you a quote for the article processing charge (APC) in case of acceptance. This quote takes into account any reduction or fee waivers that you may be eligible for. Authors do not need to pay any fees before their manuscript is accepted and transferred to the publisher.

EMBO Press participates in many Publish and Read agreements that allow authors to publish Open Access with reduced/no publication charges. Check your eligibility: https://authorservices.wiley.com/author-resources/Journal-Authors/open-access/affiliation-policies-payments/index.html

***** Reviewer's comments *****

Referee #1 (Comments on Novelty/Model System for Author):

The human skin model system is appropriate.

Referee #1 (Remarks for Author):

Trichothiodystrophy-associated MPLKIP maintains DBR1 levels for mRNA splicing and skin
Development: Arjan F. Theil, Alex Pines, Tuğba Kalayci et.al.
This is a very interesting paper which characterizes the role of MPLKIP in the development of trichothiodystrophy (TTD). Since TTD is a genetically heterogeneous disease caused by bi-allelic mutations in one of at least 10 different genes from multiple pathways. Patients with MPLKIP mutations have been termed as having TTDN1 - for non-photosensitive 1. The authors report the clinical findings of 7 TTD patients from 5 families with bi-allelic MPLKIP mutations. The authors used mass spectrometry-based interaction proteomics to determine the biological role of MPLKIP. They reported that MPLKIP interacts with core splicing factors and the lariat debranching protein DBR1. Interestingly, the MPLKIP deficient primary fibroblasts from their patients showed reduced steady-state DBR1 protein levels but not at mRNA levels. Their data shows that MPLKIP, through its DBR1 stabilizing role, is implicated in mRNA splicing, which is of particular importance in highly differentiated tissue such as skin. They suggest that the TTD-phenotype may be linked to instability of proteins involved in multiple steps of gene expression.
Suggestions for authors:
1) Abstract - The information about the cohort of patients from five unrelated families with characteristic features of TTD and defects in the TTDN1 gene should be presented in this section.
2) Introduction: A major finding of this manuscript (binding of MPLKIP protein to DBR1) was previously reported by others: Townley et al A condensate forming tether for lariat debranching enzyme is defective in non-photosensitive trichothiodystrophy https://doi.org/10.1101/2022.11.03.515106 also an abstract was published by the same group in 2021 https://doi.org/10.1096/fasebj.2021.35.S1.02893 This information should be cited.
3) Introduction is too long and has repetition of information which is presented in the results section.
4) Page 4, last paragraph, "In this paper, we report on seven novel individuals with MPLKIP deficiency, each displaying TTD associated ectodermal features, which triggered us to disclose the biological function of the MPLKIP protein to identify a rational mechanistic basis for this phenotypic expression." The "seven novel individuals" should be replaced with "seven individuals with novel germline mutations in the TTDN1 gene" as the mutations identified in these patients are novel.
5) Most of the patients were homozygous for the changes in the TTDN1 gene. Were the mutations identified in the patients also detected in their respective parents?
6) Some patients harbor identical mutations but are reported as not related. They may have common ancestor that can be confirmed by doing WES to determine if they have a shared haplotype.

Table 1. Patient Characteristics and Supplemental Data:
1. The detailed clinical evaluations and descriptions are very complete.
2. The + and - signs as well as ++, +++ should be defined. Are there objective differences between these? Although the meaning can be inferred, author defined definitions of the symbols should be in the table legend.
3. Patient TTD288IS is described as having obesity, however her weight is only 0.3 SD; is this correct? Is this a typographic error? The WHO definition of obesity is greater than +3 SD for children.
4. In subject TTD2881S - Although her BMI falls below the WHO definition for obesity, she is overweight. Based on some of her

other features (obsessive eating) was Prader-Willi Syndrome considered?
5. The patients in family 4 both have uvula abnormalities. Have they been investigated for submucous cleft palate? Please comment.
6. All patients reported in this paper have low bone density scores (all Z scores are below -2.0 SD). This has been reported in other TTD patients with MPLKIP mutations (Heller 2015; DiGiovanna 2022) and should be commented on in the discussion. This may be another example of abnormalities in terminally differentiated tissues. Which bones were assessed? Some TTD patients have peripheral osteopenia along with central osteosclerosis while others have diffuse osteopenia. (DiGiovanna 2022 - DOI: 10.1002/ajmg.a.62962) This paper should be cited.
7. Since 4 of the 5 families exhibit consanguinity, some of the patient characteristics not commonly seen in TTD may be the result of other recessive conditions. Please comment.
8. In subject TTD287IS - The diagnosis of hypogonadotropic hypogonadism may be difficult to make in a child aged 8. Were the hormone levels adjusted for age?
9. In subject TTD288IS - Why was the EEG performed? Were seizures suspected; did the EEG show epileptiform changes - if so, please specify in the text. It would be unclear why carbamazepine was started if no seizure activity was documented.
10. In subject TTD299IS - Please reconcile the pedigree with the narrative. In the pedigree only one stillbirth is noted, 4 neonatal deaths are listed not 2, the missed AB is not listed and the child who died at 16 months is not listed.
11. In pedigree C there is one additional affected patient who is dead (no number identifier). How was it determined this person was affected? Any additional information? Please describe in narrative.

Referee #2 (Comments on Novelty/Model System for Author):

Model systems employed in the study are adequate in my opinion.

Referee #2 (Remarks for Author):

In this manuscript, Theil and colleagues describe their characterization of trichothiodystrophy associated MPLKIP, its molecular functions in splicing and its role in skin development. Using affinity purification and SILAC based mass spec to define the interactome of CRISPR-tagged MPLKIP, they identify that MPLKIP interacts with components of U5 snRNP, Nineteen-complex (NTC) and debranching complex. They identify a mutual dependence of MPPLKIP and DRB1 for protein stability in variety of settings - patient derived fibroblasts, immortalized keratinocytes and human skin equivalents (HSEs), the latter also exhibiting impaired development. They find that acute depletion of MPLKIP reduces interaction of NTC component XAB2 with DBR1 but increases interaction with PPIE (U5 snRNP component). Finally, their transcriptomic and proteomic characterization of MPLKIP-deficient HSEs identifies altered gene expression profile in this model of skin development.

The study takes a systematic approach toward understanding MPLKIP function starting from patient mutations all the way to molecular characterization by employing carefully designed, facile and appropriate in vitro cell culture and organoid models. While the data that are presented are of high quality, the manuscript disappointingly falls short of providing a clear support for the main conclusion of MPLKIP's function in splicing that it is required for faithful handover from NTC to DBR1-complex. Moreover, it also fails to connect altered gene expression profiles upon MPLKIP loss with its molecular function. If the authors can remedy at least one (or both) of these issues, it will significantly improve the significance and impact of their overall work and may become suitable for this journal. Without such deeper insights, the manuscript is more suitable for a specialized journal. Below are some specific comments for consideration:

1.For a deeper more careful examination of the effect of MPLKIP loss on spliceosome subcomplexes, more U5 components should be tested in Figure 5C. In addition, it will be important to test the altered balance between NTC and debranching complex in a more direct manner. One possible way is to test if there is a higher accumulation of intron lariats in MPLKIP mutant cells. Another possibility is to examine spliceosomal complexes via glycerol gradient fractions of splicing intermediates and products (e.g., as in PMID: 27377154). If more direct and functional evidence cannot be provided for the conclusion that MPLKIP is required for faithful handover from NTC to DBR1-complex, then such a strong conclusion cannot be drawn and the text should be altered accordingly.

2.Can a more careful analysis of intronic and/or intron-exon junction reads in control versus mutant RNA-Seq samples in Fig 8 can shed more light on splicing function of MPLKIP? Previous studies have also mined for branch-sites from RNA-Seq samples (e.g., PMID: 22705790), which can be another strategy to investigate the effect of MPLKIP loss induced DBR1 reduction on splicing.

3.While the GO terms enriched among the genes upregulated at RNA level in Fig 8 include NMD and SRP-dependent protein targeting, neither the components of the NMD pathway (UPF proteins, SMG factors) nor the signal recognition particle components are among the genes upregulated >1.5 fold (padj <0.05). It is possible that these GO terms show an enrichment because several components of the ribosome are among this upregulated list. Indeed, all terms shown in the left plot of Fig 8C

are likely ribosome-linked. This underlying issue will be more apparent if term "ribosome" shows enrichment in cellular component category of the DAVID analysis. This inconsistency also points out that a deeper dive into the transcriptomic (and proteomic) data is necessary to better comprehend the altered trends in MPLKIP depleted cells and to start uncovering more mechanistic details of MPLKIP function.

4. While in Fig 8D and 8E the shorter gene length and fewer exon number of the upregulated genes is an interesting trend, could this again be driven by ribosomal protein genes that are short and have fewer exons?

5.How well do up- and down-regulated genes or RNA (Fig 8) correlate with proteins (Fig 9)?

6.Is reduced DBR1 level observed in the mass spec data in Figure 8? Are any other DBR1-assocoated proteins also affected in this analysis?

7.It is likely that MPLKIP loss alters expression of one or a few key regulators which then cause downstream effects on skin development and associated gene expression. Does a close examination of the omic data point to any such regulators? If yes, is such a defect due to altered splicing? Also, if such regulators are identified, restoration of their expression in MPLKIP deficient cells may rescue the developmental phenotypes (at least partially). Such an insight will further increase the impact of the work.

Response to the decision letter of EMM-2023-17973

Referee #1 (Comments on Novelty/Model System for Author):

The human skin model system is appropriate.

Referee #1 (Remarks for Author):

Trichothiodystrophy-associated MPLKIP maintains DBR1 levels for mRNA splicing and skin Development: Arjan F. Theil, Alex Pines, Tuğba Kalayci et.al.
This is a very interesting paper which characterizes the role of MPLKIP in the development of trichothiodystrophy (TTD). Since TTD is a genetically heterogeneous disease caused by bi-allelic mutations in one of at least 10 different genes from multiple pathways. Patients with MPLKIP mutations have been termed as having TTDN1 - for non-photosensitive 1. The authors report the clinical findings of 7 TTD patients from 5 families with bi-allelic MPLKIP mutations. The authors used mass spectrometry-based interaction proteomics to determine the biological role of MPLKIP. They reported that MPLKIP interacts with core splicing factors and the lariat debranching protein DBR1. Interestingly, the MPLKIP deficient primary fibroblasts from their patients showed reduced steady-state DBR1 protein levels but not at mRNA levels. Their data shows that MPLKIP, through its DBR1 stabilizing role, is implicated in mRNA splicing, which is of particular importance in highly differentiated tissue such as skin. They suggest that the TTD-phenotype may be linked to instability of proteins involved in multiple steps of gene expression.
Suggestions for authors:

We thank the reviewer for the appreciation of our work and experimental procedures.

1) Abstract - The information about the cohort of patients from five unrelated families with characteristic features of TTD and defects in the TTDN1 gene should be presented in this section.

We thank the reviewer for pointing out this, we agree that it is indeed important to implement this information. We have therefore implemented the following sentence in the abstract:
"*Here, we present seven additional TTD individuals with MPLKIP mutations from five consanguineous families, with a newly identified MPLKIP variant in one family*"

2) Introduction: A major finding of this manuscript (binding of MPLKIP protein to DBR1) was previously reported by others: Townley et al A condensate forming tether for lariat debranching enzyme is defective in non-photosensitive trichothiodystrophy https://doi.org/10.1101/2022.11.03.515106 also an abstract was published by the same group in 2021 https://doi.org/10.1096/fasebj.2021.35.S1.02893 This information should be cited.

Indeed, a non-peer reviewed manuscript by Townley et al was deposited to bioRxiv, in which also MPLKIP association to DBR1 was reported. We thank the reviewer for pointing out this and have now included a citation to this work. During the reviewing process of our manuscript the work by Townly et al has now been published as a peer-reviewed manuscript in Molecular Cell and we have included the citation to this work.

3) Introduction is too long and has repetition of information which is presented in the results section.

Our manuscript aims to ensure accessibility to a wide range of readers, taking into consideration the complexity of the genetic disease Trichothiodystrophy (TTD) discussed in this study. We believe that the introduction provides necessary information without being excessively lengthy or repetitive.

Given the intricate nature of the topic, it is essential to guide readers comprehensively and engage them throughout the narrative. Regarding the last paragraph of the introduction, we acknowledge that it summarizes the main results presented in the paper, which may appear repetitive. However, we followed the general format commonly found in papers published in EMBO Molecular Medicine, which often include a concise summary of the main data and conclusions at the end of the Results section. After consultation with the editor, it is decided to leave the summary part in.

4) Page 4, last paragraph, "In this paper, we report on seven novel individuals with MPLKIP deficiency, each displaying TTD associated ectodermal features, which triggered us to disclose the biological function of the MPLKIP protein to identify a rational mechanistic basis for this phenotypic expression." The "seven novel individuals" should be replaced with "seven individuals with novel germline mutations in the TTDN1 gene" as the mutations identified in these patients are novel.

We apologize for this confusing phrasing. We have slightly rephrased/adpated the sentence. It should be noted however, that only in two sibs we have identified a novel mutation, the mutations found in the other five patients, from four different families, were actually earlier reported, as we have mentioned in the first part of the results session (cited from there: "*The latter two mutations were previously found to be associated with TTD*.")

5) Most of the patients were homozygous for the changes in the TTDN1 gene. Were the mutations identified in the patients also detected in their respective parents?

Yes, we did perform DNA sequencing on several family members to investigate disease segregation. We did not include this data in the original manuscript, as two mutations (found in four families) have been reported earlier and shown to be pathogenic. The newly identified mutations result in an early frameshift. These findings strongly support the conclusion that expression of the full length MPLKIP is compromised in all TTD individuals described in this manuscript (as shown in the Western blot, Figure 4A, no MPLKIP protein is detected) and is causative for the genetic disorder TTD. We mentioned this in the section of the results: "Considering allele frequency, predicted pathogenicity of the identified variants, patient phenotypes that are highly specific for TTD, and ***co-segregation*** with disease in multiple family members."
In the revised manuscript we have now implemented the segregation data in the Expanded View (Fig EV1) and refer to that in the text.

6) Some patients harbor identical mutations but are reported as not related. They may have common ancestor that can be confirmed by doing WES to determine if they have a shared haplotype.

Indeed, it would be interesting to chase for a common haplotype among the different families that carry the same mutation. However, our focus in this manuscript is not so much on searching the origin of the specific mutation but more on disclosing the biological function of the MPLKIP/TTDN1 protein.
After consultation with the editor, it is decided not to include this in revised manuscript, as the search for a common ancestor of identical mutations from different families is beyond the scope of this manuscript.

Table 1. Patient Characteristics and Supplemental Data:
1. The detailed clinical evaluations and descriptions are very complete.

We thank the reviewer for this nice supportive comment.

2. The + and - signs as well as ++, +++ should be defined. Are there objective differences between

these? Although the meaning can be inferred, author defined definitions of the symbols should be in the table legend.

The '+' and '-' signs were now defined in the Table 1 legends.

Formal assessments regarding the degree of speech delays are based on severity rating scales performed by developmentalists & speech specialists, which could not be performed in our patients. Our assessments were informal. According to the informal definition, severe expressive language deficit is described as "very limited functional language skills where communication requires serious effort". According to this, all of our patients have severe expressive language deficit (e.g. none of them could build three-word sentences and were limited to below 50 words at best). To avoid confusion, we now refrain from using "+", "++", and "+++" to describe severity, and just use a "+" to denote the presence of a severe language delay. The difference in severity among our patients could be inferred from the upper rows describing the time at first words and first two-word sentences.

3. Patient TTD288IS is described as having obesity, however her weight is only 0.3 SD; is this correct? Is this a typographic error? The WHO definition of obesity is greater than +3 SD for children.

We apologize for the confusion, the descriptor "obese" was changed to "overweight" according to the WHO definition in the text and table.

4. In subject TTD2881S - Although her BMI falls below the WHO definition for obesity, she is overweight. Based on some of her other features (obsessive eating) was Prader-Willi Syndrome considered?

Prader-Willi syndrome can be considered in the differential diagnosis of Patient TTD2881S due to history of neonatal hypotonia, global developmental delay, hypogonadism and excessive appetite. However, we had not considered it to be the clinical diagnosis, because: (1) all of these findings were also characteristic for TTD except hyperphagia; (2) dysmorphic findings were not reminiscent of PWS and characteristic PWS findings such as small and narrow hands and feet were not present. Still, a methylation specific MLPA for the Prader-Willi critical region (PWCR) was performed with SALSA MLPA Probemix ME028 Prader-Willi/Angelman and the disease was molecularly ruled out. This is now included in the clinical narrative.

5. The patients in family 4 both have uvula abnormalities. Have they been investigated for submucous cleft palate? Please comment.

By physical examination including inspection and palpation, apparent submucosal clefts were ruled out in both patients. Absence of findings comprising nasal speech, recurrent otitis media, and neonatal respiratory & feeding difficulties were also supportive for an intact palate.

6. All patients reported in this paper have low bone density scores (all Z scores are below -2.0 SD). This has been reported in other TTD patients with MPLKIP mutations (Heller 2015; DiGiovanna 2022) and should be commented on in the discussion. This may be another example of abnormalities in terminally differentiated tissues. Which bones were assessed? Some TTD patients have peripheral osteopenia along with central osteosclerosis while others have diffuse osteopenia. (DiGiovanna 2022 - DOI: 10.1002/ajmg.a.62962) This paper should be cited.

We thank to the reviewer for bringing this to our attention. This is an interesting finding reported in several cases with TTD. In this light, we reviewed our patients' files for the results of BMD evaluations. Unfortunately, only lumbar bone densities were recorded, showing reduced BMD in all patients. This gives us the chance to exclude central osteosclerosis. We now mentioned this in the

first paragraph of discussion as "*Central osteosclerosis, a common finding in TTD patients, was ruled out in all our patients*"; and cited the paper reported by DiGiovanna *et al.*.

7. Since 4 of the 5 families exhibit consanguinity, some of the patient characteristics not commonly seen in TTD may be the result of other recessive conditions. Please comment.

We agree that part of these features may be the result other recessive conditions. Indeed, in the presence of consanguinity, multiple Mendelian disorders in the same patient can cause a blended phenotype, complicating diagnosis and disease characterization. Since the molecular diagnosis of our patients were ascertained with Sanger sequencing of MPLKIP, we did not rule out the presence of a concomitant genetic disorder that could possibly be attributed to the atypical midline findings in our patients. Further patient reports would be required to include bifid uvula and single central incisor as part of the TTDN phenotype.
In the revised manuscript we now mention this in the relevant part of the discussion, which reads: "*However, we have not ruled out the presence of a concomitant genetic disorder that could possibly be attributed to the atypical midline findings in our patients. Thus, further patient reports are required to include these findings as a part of the TTDN phenotype*."

8. In subject TTD287IS - The diagnosis of hypogonadotropic hypogonadism may be difficult to make in a child aged 8. Were the hormone levels adjusted for age?

We thank the reviewer to point this out. We made a mistake in prematurely classifying some of our patients as having hypogonadotropic hypogonadism. According to the 2015 European Consensus Statement on congenital hypogonadotropic hypogonadism [PMID: 26194704], the diagnosis is difficult to discern from pubertal delay. At this stage, a definitive diagnosis is only enabled when pubertal induction is commenced and followed up by a clinical and biochemical reassessment following final height. Below that age, the condition should be more properly defined as pubertal delay.

To clarify the diagnosis, we repeated the clinical examination and reevaluated gonadotropin levels in three of our patients: patient TTD287IS (at age $15^{4/12}$ years), TTD289IS (at $17^{7/12}$ years) and TTD290IS (at $19^{6/12}$ years) in June 2023. We also checked patient files of Patient TTD288IS at 14 years for gonadotropin levels. Rather than having hypogonadotropic hypogonadism, all of these four patients had endocrinological results compatible with hypergonadotropic hypogonadism, increasing the number of patients with hypergonadotropic hypogonadism to five.

Two patients (patients TTD295IS and TTD303IS) were last evaluated at $14^{6/12}$ (the current age is $15^{3/12}$ years) and 17 years (the current age is 24 years), and both had prepubertal gonadotropin levels with immature sexual characteristics. While the former had bilateral cryptorchidism and micropenis suggestive of congenital hypogonadotropic hypogonadism, the latter had normal testicular position and penis size for age. More recent evaluations could not be performed. According to the 2015 Consensus Statement and per the Reviewer's reminder, it will be more appropriate to use the term "delayed puberty" instead of "hypogonadotropic hypogonadism" in these two male patients. Please note that the age of pubertal onset is predicted as 10.5±0.5 years in Turkish boys [PMID: 19209617].

Within the revised Table and the clinical narrative these findings were described.

9. In subject TTD288IS - Why was the EEG performed? Were seizures suspected; did the EEG show epileptiform changes - if so, please specify in the text. It would be unclear why carbamazepine was started if no seizure activity was documented.

In small children with global developmental delay and delayed myelination on MRI, EEG is included in the neurology work-up to rule out epileptic encephalopathies. This is why a pediatric neurologist had performed an EEG at 15 months of age, even though the family did not describe any clinical seizures. The EEG showed epileptiform changes, but the neurologist did not administer an antiepileptic drug. The child has not had any seizures ever since. Carbamazepine was administered as a mood stabilizer to control over-activity and aggressive behaviour at the age of eight years and was discontinued after the age of 17. We have now included this in the patient's history in "Appendix Supplemental clinical data".

10. In subject TTD299IS - Please reconcile the pedigree with the narrative. In the pedigree only one stillbirth is noted, 4 neonatal deaths are listed not 2, the missed AB is not listed and the child who died at 16 months is not listed.

We thank the reviewer for alerting us to the omissions. The family has a history of two stillbirths, three neonatal deaths with unknown aetiology, and a child with hypotonia and growth retardation who eventually died at 16 months of age. We revised the pedigree in that direction (Figure 1E) and the clinical narrative is now compatible with the pedigree.

11. In pedigree C there is one additional affected patient who is dead (no number identifier). How was it determined this person was affected? Any additional information? Please describe in narrative.

This deceased individual and his family had been living in Bremen, Germany. Although we could not evaluate him in our clinics, we had the chance to review his patient files from the University of Bremen. The physician noted that he had "hair shaft anomaly, short stature, microcephaly, developmental delay, and epilepsy" which raised suspicion for a diagnosis of "Tay syndrome". This is why we expect the patient as affected. We have not included the patient in our cohort, since we have not had the chance to make further contact with the family.

Referee #2 (Comments on Novelty/Model System for Author):

Model systems employed in the study are adequate in my opinion.

Referee #2 (Remarks for Author):

In this manuscript, Theil and colleagues describe their characterization of trichothiodystrophy associated MPLKIP, its molecular functions in splicing and its role in skin development. Using affinity purification and SILAC based mass spec to define the interactome of CRISPR-tagged MPLKIP, they identify that MPLKIP interacts with components of U5 snRNP, Nineteen-complex (NTC) and debranching complex. They identify a mutual dependence of MPPLKIP and DRB1 for protein stability in variety of settings - patient derived fibroblasts, immortalized keratinocytes and human skin equivalents (HSEs), the latter also exhibiting impaired development. They find that acute depletion of MPLKIP reduces interaction of NTC component XAB2 with DBR1 but increases interaction with PPIE (U5 snRNP component). Finally, their transcriptomic and proteomic characterization of MPLKIP-deficient HSEs identifies altered gene expression profile in this model of skin development.

The study takes a systematic approach toward understanding MPLKIP function starting from patient mutations all the way to molecular characterization by employing carefully designed, facile and appropriate in vitro cell culture and organoid models. While the data that are presented are of high quality, the manuscript disappointingly falls short of providing a clear support for the main conclusion of MPLKIP's function in splicing that it is required for faithful handover from NTC to DBR1-complex. Moreover, it also fails to connect altered gene expression profiles upon MPLKIP loss with its molecular function. If the authors can remedy at least one (or both) of these issues, it will significantly improve the significance and impact of their overall work and may become suitable for this journal. Without such deeper insights, the manuscript is more suitable for a specialized journal. Below are some specific comments for consideration:

We appreciate the reviewer's appreciation on the experimental design and acknowledging the high quality of the experimental work. We also thank her/him for the valuable feedback and main suggestions/comments below and will make sure to clarify these points in the revised manuscript.

Firstly, we acknowledge that our study does not provide a detailed molecular mechanism of MPLKIP's function in splicing or intron lariat dynamics. This was not the primary objective of our research, which aimed to elucidate the biological function of the previously uncharacterized MPLKIP gene product. We want to clarify that our title, abstract, and introduction-associated summary of conclusions do not make any explicit claims or draw conclusions regarding the faithful handover from the NTC to the DBR1 complex. We think that it is fair to say that our provided evidence is sufficient to proof that MPLKIP functions in transcript maturation and required for proper skin differentiation. Our conclusions are based on the following evidence: (I) MPLKIP's association with spliceosome complexes. (II) Its requirement for stabilizing DBR1. (III) The observed alterations in the transcriptome, including aberrant splice products, in the absence of MPLKIP. (IV) The dysregulated proteome and differentiation observed in our 3D skin model in the absence of MPLKIP. Understanding the biological consequences of this role is particularly important in relation to epithelial differentiation and the recurrent infections commonly associated with TTD.

1.For a deeper more careful examination of the effect of MPLKIP loss on spliceosome subcomplexes, more U5 components should be tested in Figure 5C. In addition, it will be important to test the altered balance between NTC and debranching complex in a more direct manner. One possible way is to test if there is a higher accumulation of intron lariats in MPLKIP mutant cells. Another possibility is to examine spliceosomal complexes via glycerol gradient fractions of splicing intermediates and

products (e.g., as in PMID: 27377154). If more direct and functional evidence cannot be provided for the conclusion that MPLKIP is required for faithful handover from NTC to DBR1-complex, then such a strong conclusion cannot be drawn and the text should be altered accordingly.

In accord to this reviewer's suggestion, we have conducted a thorough analysis of spliceosome subcomplexes in an XAB2 pull-down experiment following MPLKIP depletion, as depicted in Figure 5C. Specifically, we focused on U5 components such as PRPF6, PRPF8, and PRPF31. Our findings indicate that the U5 components do not exhibit a higher association with the NTC complex, but mainly shows a decreased association with DBR1 upon MPLKIP depletion. We have incorporated these new data into the manuscript (see figure below and revised Fig 5C in the revised manuscript). We acknowledge the reviewer's remark on our previous suggestion of an impaired balance between the NTC and the debranching complex, in accordance with the newly added experiments we have revised our conclusions on this matter. Instead of emphasizing the overall impact of MPLKIP deficiency on the handover from NTC to DBR1-complex, we focus on the decreased interaction of the AQR/XAB2 complex with DBR1 in the absence of MPLKIP. This adjustment will indeed more accurately reflect the specific effect observed in our study. We thank the reviewer for pointing this out.

We have also implemented new experimental data to identify intron lariats, based on a pipeline as described by Pineda and Bradley *et al*. (2018) to detect these structures in our RNA sequencing data. Our analysis revealed a significant increase (11.8-fold enrichment) in lariat sequences in MPLKIP-deficient samples compared to control samples. We have included these results and their implications in the manuscript (see Figure below and the newly added Figure EV5E).

Additionally, the observed accumulation of lariat introns in our study is consistent with ample literature evidence that depletion or mutant forms of DBR1 result in lariat-intron accumulation and are associated with severe human disease conditions (e.g., Mohanta et al., 2021; Zhang et al., 2018). These findings further support the functional relevance of MPLKIP, through its protein-stabilizing interaction with DBR1, in transcript maturation processes.

2.Can a more careful analysis of intronic and/or intron-exon junction reads in control versus mutant RNA-Seq samples in Fig 8 can shed more light on splicing function of MPLKIP? Previous studies have also mined for branch-sites from RNA-Seq samples (e.g., PMID: 22705790), which can be another strategy to investigate the effect of MPLKIP loss induced DBR1 reduction on splicing.

While we acknowledge that a detailed molecular mechanism describing MPLKIP's function in splicing is not extensively addressed in this study, our primary objective was to demonstrate the impact of MPLKIP deficiency on splicing, transcriptome, and eventually proteome consequences in highly differentiated tissue, which is applied as a relevant experimental proxy to investigate part of the clinical features of TTD.

However, we appreciate the reviewer's suggestion to explore the RNA sequencing data further if possible. In an attempt to address this, we specifically analysed the branch-point nucleotide of the lariats obtained from our RNA sequencing data. However, we encountered a limitation in our analysis due to the low number of lariats detected in the splicing-proficient control samples. As a result, we lacked the statistical power to thoroughly investigate differences in the branch-point location between the two groups. We hope the reviewer understands that to compensate this this limitation, we have now included the data on the accumulation of lariat introns in the Expanded View section, as explained above.

A more comprehensive investigation to precisely disclose the molecular mechanisms underlying MPLKIP's function in mRNA maturation requires additional studies that go beyond the scope of this manuscript. Our main aim was to establish the broader impact of MPLKIP deficiency on gene expression in the context of TTD, as thus far the biological role for MPLKIP/TTDN1 was unknown. We believe that the findings presented in this study contribute to our understanding of MPLKIP's role in gene expression through its role in splicing and its relevance to TTD pathogenesis.

3.While the GO terms enriched among the genes upregulated at RNA level in Fig 8 include NMD and SRP-dependent protein targeting, neither the components of the NMD pathway (UPF proteins, SMG factors) nor the signal recognition particle components are among the genes upregulated >1.5 fold (padj <0.05).
It is possible that these GO terms show an enrichment because several components of the ribosome are among this upregulated list. Indeed, all terms shown in the left plot of Fig 8C are likely ribosome-linked. This underlying issue will be more apparent if term "ribosome" shows enrichment in cellular component category of the DAVID analysis. This inconsistency also points out that a deeper dive into the transcriptomic (and proteomic) data is necessary to better comprehend the altered trends in MPLKIP depleted cells and to start uncovering more mechanistic details of MPLKIP function.

We acknowledge that our STRING analysis showed a predominance of ribosomal genes rather than core NMD/SRP-associated upregulated genes. This is consistent with the enrichment of the "ribosome" cellular component category in the DAVID analysis. We did not intend to overemphasize these upregulated pathways. In light of the reviewer's feedback, we have toned down the discussion on the NMD and SRP pathways. Instead, we highlight that the upregulation of these pathways can be seen as a possible cellular response to the observed aberrant splice products. Furthermore, we

mention that ribosome protein genes are particularly affected, these adaptations to the text are incorporated at the end of the sixth paragraph of the discussion section.

4. While in Fig 8D and 8E the shorter gene length and fewer exon number of the upregulated genes is an interesting trend, could this again be driven by ribosomal protein genes that are short and have fewer exons?

Indeed, part of the up-regulated genes affected by MPLKIP deficiency consists of ribosomal protein encoding genes. This observation aligns with the GO term analysis presented in Figure 8C. Furthermore, even after excluding the ribosomal genes from the analysis, we still clearly observe that the genes in TTD>ctrl exhibit a lower number of exons compared to TTD<ctrl genes. Therefore, there remains a notable difference in the average number of exons among the TTD-upregulated genes, even when the ribosomal genes are not considered (See picture below).

5.How well do up- and down-regulated genes or RNA (Fig 8) correlate with proteins (Fig 9)?

We would like to emphasize that RNA sequencing primarily provides information on mRNA expression levels, while protein levels are influenced by various post-translational modifications and

other factors and therefore cannot be in many cases directly correlated to mRNA levels. Despite observing similarities in affected proteins and pathways between the two analyses, it is important to note that many identified genes/proteins are not identical in both datasets. This underscores the complex relationship between RNA and protein levels and highlights the complementary nature of these two types of data, rather than always a direct correlation. This is also the exact reason why we have performed both complementary procedures. We have specifically addressed these differences and complexities in the eighth paragraph of the discussion of the revised manuscript, where we highlight briefly the complexities associated with the relationship between RNA and protein levels, including relevant references ((Vogel & Marcotte, 2012; Maier *et al*, 2009)).

6.Is reduced DBR1 level observed in the mass spec data in Figure 8? Are any other DBR1-assocoated proteins also affected in this analysis?

Our proteomic analysis did not yield a sufficient number of DBR1 peptides for reliable quantification in the mass spectrometry data presented in Figure 8. However, we were able to identify several other proteins known to interact with MPLKIP. These proteins, including CWF19L1, AQR, PRPF6, PRPF8, HNRNPU, SNRNP200, and PPIE, showed no significant changes in abundance or alterations in their levels.

7.It is likely that MPLKIP loss alters expression of one or a few key regulators which then cause downstream effects on skin development and associated gene expression. Does a close examination of the omic data point to any such regulators? If yes, is such a defect due to altered splicing? Also, if such regulators are identified, restoration of their expression in MPLKIP deficient cells may rescue the developmental phenotypes (at least partially). Such an insight will further increase the impact of the work.

We apologize for not being clear on this. Yes, indeed we have identified several key regulators of skin differentiation, most of which are associated with inherited skin disorders, illustrating their key role in skin differentiation. A few examples were explicitly mentioned in the discussion such as COL7A1 (support extracellular matrix (ECM) of skin), KRT4 & KRT13 (vital for epidermal morphology), DSC2, SPRR2 (crosslinks proteins and previously contributes to the skin host defense). To emphasize these key regulators, we have changed the text in the discussion as follows: "*Proteome analysis further identified significant down regulation of a large group of keratinocyte-specific structural and ECM-associated key regulatory proteins, including collagens, keratins, desmocollins, transglutaminases and members of the small proline-rich protein (SPRR) family.*"

We appreciate the reviewer's challenging suggestion regarding the restoration of key regulators. However, identifying specific key regulators responsible for the downstream effects on skin development would be a rather challenging enterprise, beyond the scope of this manuscript but certainly valuable for follow up experiments. Additionally, generating knock-ins in primary cells, particularly in the primary fibroblasts obtained from *MPLKIP*-deficient individuals, remains a difficult task. We recognize that further investigations and technical advancements are needed to overcome the obstacles associated with generating knock-ins in primary cells. However, we believe that prioritizing the exploration of DBR1 protein expression as a potential rescue mechanism holds promise for understanding and addressing the developmental phenotypes associated with *MPLKIP* deficiency.

8th Sep 2023

Dear Prof. Vermeulen,

Thank you for the submission of your revised manuscript to EMBO Molecular Medicine. I am pleased to inform you that we will be able to accept your manuscript pending the following final amendments:

1) Please address all referee #2 points by implementing suggested adjustments in the figures and the text and by discussion. No additional experiments are required.
2) Patient information: Please include all clinical information, that were previously in the Appendix, in M&M section in main manuscript text. Also, include statement that the informed consent was obtained from all human subjects and that the experiments conformed to the principles set out in the WMA Declaration of Helsinki and the Department of Health and Human Services Belmont Report.
3) In the main manuscript file, please do the following:
- In M&M, provide the antibody dilutions that were used for each antibody.
- In M&M, add the following paragraph:

Graphics:
(some of the... OR Figure #... OR synopsis) Graphics were created with BioRender.com.

Please remove the reference to BioRender from the "Acknowledgements".
- In M&M, add statistical paragraph that should reflect all information that you have filled in the Authors Checklist, especially regarding randomization, blinding, replication.
- Author contributions: Please remove it from the manuscript and specify author contributions in our submission system. CRediT has replaced the traditional author contributions section because it offers a systematic machine-readable author contributions format that allows for more effective research assessment. You are encouraged to use the free text boxes beneath each contributing author's name to add specific details on the author's contribution. More information is available in our guide to authors:
https://www.embopress.org/page/journal/17574684/authorguide#authorshipguidelines
4) Table: Please rename the Table 1 to Table EV1, update its callout in the text and remove the legend from the manuscript and add it to the excel file.
5) Synopsis:
- Please resize the synopsis image to 550 px-wide x (250-400)-px high and submit it as a high-resolution jpeg file.
- Please check your synopsis text and image before submission with your revised manuscript. Please be aware that in the proof stage minor corrections only are allowed (e.g., typos).
6) Source data: Please upload source data as one (zipped) file per main figure, i.e. all the source data files related to a single main figure should be zipped into one zip file. Source data for all EV figure should remain as one zip file.
7) As part of the EMBO Publications transparent editorial process initiative (see our Editorial at http://embomolmed.embopress.org/content/2/9/329), EMBO Molecular Medicine will publish online a Review Process File (RPF) to accompany accepted manuscripts. This file will be published in conjunction with your paper and will include the anonymous referee reports, your point-by-point response and all pertinent correspondence relating to the manuscript. Let us know whether you agree with the publication of the RPF and as here, if you want to remove or not any figures from it prior to publication. Please note that the Authors checklist will be published at the end of the RPF.
8) Please provide a point-by-point letter INCLUDING my comments as well as the reviewer's reports and your detailed responses (as Word file).

I look forward to reading a new revised version of your manuscript as soon as possible.

Yours sincerely,

Zeljko Durdevic

Zeljko Durdevic
Editor
EMBO Molecular Medicine

*** Instructions to submit your revised manuscript ***

*** PLEASE NOTE *** As part of the EMBO Publications transparent editorial process initiative (see our Editorial at https://www.embopress.org/doi/pdf/10.1002/emmm.201000094), EMBO Molecular Medicine will publish online a Review Process File to accompany accepted manuscripts.

In the event of acceptance, this file will be published in conjunction with your paper and will include the anonymous referee reports, your point-by-point response and all pertinent correspondence relating to the manuscript. If you do NOT want this file to be published, please inform the editorial office at contact@embomolmed.org.

When submitting your revised manuscript, please include:

1) a .docx formatted version of the manuscript text (including Figure legends and tables)

2) Separate figure files*

3) supplemental information as Expanded View and/or Appendix. Please carefully check the authors guidelines for formatting Expanded view and Appendix figures and tables at https://www.embopress.org/page/journal/17574684/authorguide#expandedview

4) a letter INCLUDING the reviewer's reports and your detailed responses to their comments (as Word file).

5) The paper explained: EMBO Molecular Medicine articles are accompanied by a summary of the articles to emphasize the major findings in the paper and their medical implications for the non-specialist reader. Please provide a draft summary of your article highlighting
- the medical issue you are addressing,
- the results obtained and
- their clinical impact.
This may be edited to ensure that readers understand the significance and context of the research.
Please refer to any of our published articles for an example.

6) For more information: There is space at the end of each article to list relevant web links for further consultation by our readers. Could you identify some relevant ones and provide such information as well? Some examples are patient associations, relevant databases, OMIM/proteins/genes links, author's websites, etc...

7) Author contributions: the contribution of every author must be detailed in a separate section.

8) EMBO Molecular Medicine now requires a complete author checklist (https://www.embopress.org/page/journal/17574684/authorguide) to be submitted with all revised manuscripts. Please use the checklist as guideline for the sort of information we need WITHIN the manuscript. The checklist should only be filled with page numbers were the information can be found. This is particularly important for animal reporting, antibody dilutions (missing) and exact values and n that should be indicted instead of a range.

9) Every published paper now includes a 'Synopsis' to further enhance discoverability. Synopses are displayed on the journal webpage and are freely accessible to all readers. They include a short stand first (maximum of 300 characters, including space) as well as 2-5 one sentence bullet points that summarise the paper. Please write the bullet points to summarise the key NEW findings. They should be designed to be complementary to the abstract - i.e. not repeat the same text. We encourage inclusion of key acronyms and quantitative information (maximum of 30 words / bullet point). Please use the passive voice. Please attach these in a separate file or send them by email, we will incorporate them accordingly.

You are also welcome to suggest a striking image or visual abstract to illustrate your article. If you do please provide a jpeg file 550 px-wide x 400-px high.

10) A Conflict of Interest statement should be provided in the main text

11) Please note that we now mandate that all corresponding authors list an ORCID digital identifier. This takes <90 seconds to complete. We encourage all authors to supply an ORCID identifier, which will be linked to their name for unambiguous name identification.

Currently, our records indicate that the ORCID for your account is 0000-0003-3616-734X.

Please click the link below to modify this ORCID:
Link Not Available

12) The system will prompt you to fill in your funding and payment information. This will allow Wiley to send you a quote for the article processing charge (APC) in case of acceptance. This quote takes into account any reduction or fee waivers that you may be eligible for. Authors do not need to pay any fees before their manuscript is accepted and transferred to our publisher.

*Additional important information regarding Figures

Each figure should be given in a separate file and should have the following resolution:
Graphs 800-1,200 DPI
Photos 400-800 DPI
Colour (only CMYK) 300-400 DPI"

Figures are not edited by the production team. All lettering should be the same size and style; figure panels should be indicated by capital letters (A, B, C etc). Gridlines are not allowed except for log plots. Figures should be numbered in the order of their appearance in the text with Arabic numerals. Each Figure must have a separate legend and a caption is needed for each panel.

*Additional important information regarding figures and illustrations can be found at https://bit.ly/EMBOPressFigurePreparationGuideline. See also figure legend preparation guidelines: https://www.embopress.org/page/journal/17574684/authorguide#figureformat

The system will prompt you to fill in your funding and payment information. This will allow Wiley to send you a quote for the article processing charge (APC) in case of acceptance. This quote takes into account any reduction or fee waivers that you may be eligible for. Authors do not need to pay any fees before their manuscript is accepted and transferred to our publisher.

***** Reviewer's comments *****

Referee #1 (Comments on Novelty/Model System for Author):

Seven new families are described with detailed clinical information. The experiments are well performed and the newly modified conclusions are consistent with the data presented.

Referee #1 (Remarks for Author):

The authors have responded adequately to the extensive suggestions. This manuscript provides additional support for the hypothesis of TTD being predominately a disease of transcription and RNA processing.

Referee #2 (Comments on Novelty/Model System for Author):

I selected "medium" for technical quality as it is unclear if replicates are technical or biological, at all places indicated by the authors.

Referee #2 (Remarks for Author):

The revised manuscript by Theil et al addresses many but not all issues raised on the original version. The additions and changes have certainly improved the manuscript. However, there are many issues that remain and need to be still addressed.
Main points:
The analysis in Fig EV5E is a nice addition as it further supports the authors claim on the direct effects of MPLKIP on splicing via DBR1. It is strongly recommended to move this figure to the main figures, Fig 7. Also, the description in methods on how the lariat-specific reads were identified is not fully comprehensible. While the authors refer to Pineda and Bradley (2018) for how this analysis was executed and describe how reads mapping to both 5'- and 3'-splice sites were identified, it is difficult to follow their logic as to how the split-read alignments were determined to originate from lariats. Perhaps, a schematic summarizing their approach can be added to this figure panel.
Another major issue the authors need to properly acknowledge is the recently published article by Townley et al (2023), which describes many similar findings regarding MPLKIP function alongside DBR1 in splicing, higher lariat accumulation upon MPLKIP deficiency, and the gene-length dependent effect of this deficiency on gene expression. What differentiates the two studies is that Townley et al mainly employ transformed human cell lines or mouse models unlike this study which uses a more disease-

relevant in vitro cell model. A discussion of the similar findings from this parallel work in Discussion is warranted. In my opinion, such an open acknowledgement and discussion of similar conclusions of the two studies should not lessen the impact of findings described here although I am not familiar with the journal policy regarding the impact of a competing publication with similar results on the novelty of the work under review. Lastly, it is quite easy to spot the uncanny similarity in the lariat read quantification strategy described by the authors in the revised manuscript and that described by Townley et al, although both base this analysis on Pineda and Bradley (2018).

Although the authors acknowledge that enrichment of NMD and SRP pathways in GO term analysis is mainly driven by ribosome components, they still describe alteration of NMD as a potential mechanism whereby MPLKIP deficient cells attempt to achieve homoeostasis in gene expression (Discussion, page 17). This is misleading and should be corrected. Instead, the focus can be alteration of ribosomal gene expression, possibly to modulate translation. Also, linking ribosome upregulation to "enhancing the cell's capacity to detect and eliminate improperly spliced RNA" is too speculative.

The authors point about lack of a tight correlation between RNA and protein expression is well-taken. Still, it is worth testing if at least the trend in the effects of gene length on alteration in RNA expression are also mirrored in changes in protein expression. That is, are upregulated proteins in Fig 8B from shorter genes and downregulated proteins encoded from longer genes?

In Discussion, the authors should more clearly explain their reasoning to assume that intron debranching, a step that occurs during or after spliceosome disassembly, affects the splicing process.

In figure legends, replicates for experiments are described as "technical replicates". Were biological replicates never performed or is there a confusion between the two terms? Biological replication is crucial for robust experimentation. For example, in Fig EV4, to fully demonstrate that the level of DBR1 mRNA is not changing in TTD287IS and TTD289IS biological replicates of the RT-qPCR data will be needed.

Minor points:

In the revised title, wording "correct splicing" is awkward. Also, wouldn't "keratinocyte differentiation" be more appropriate?

Fig 5 title: It is not appropriate to use a descriptive adjective like "proper" to describe the effect of MPLKIP absence on DBR1 binding to NTC.

Fig 4C- both non-CTR samples are labeled as TTD287IS.

Pg12- The authors do not show the results of the DEXSeq anywhere in figures or tables. They direct the readers to figure 8b, but that is a volcano plot based on DESeq2 results.

Pg12, line 18- Why figure 4 is referred to here? It does not relate to the results being discussed.

Pg 15-16, in Discussion paragraph starting "Although no clear functional domain...", there is undue emphasis on IDR of MPLKIP although there are no direct experiments performed by the authors in the manuscript. The speculation about two possible functions of MPLKIP can still be made without going into the possibility of phase separation.

Discussion is too speculative at places (some instances pointed out above), especially when trying to explain the alterations in gene expression identified in the last two figures.

***** Reviewer's comments *****

Referee #1 (Comments on Novelty/Model System for Author):

Seven new families are described with detailed clinical information. The experiments are well performed and the newly modified conclusions are consistent with the data presented.

Referee #1 (Remarks for Author):

The authors have responded adequately to the extensive suggestions. This manuscript provides additional support for the hypothesis of TTD being predominately a disease of transcription and RNA processing.

We thank referee #1 for the critical review and valuable remarks, which have significantly improved the paper.

Referee #2 (Comments on Novelty/Model System for Author):

I selected "medium" for technical quality as it is unclear if replicates are technical or biological, at all places indicated by the authors.

Referee #2 (Remarks for Author):

The revised manuscript by Theil et al addresses many but not all issues raised on the original version. The additions and changes have certainly improved the manuscript. However, there are many issues that remain and need to be still addressed.

Main points:
The analysis in Fig EV5E is a nice addition as it further supports the authors claim on the direct effects of MPLKIP on splicing via DBR1. It is strongly recommended to move this figure to the main figures, Fig 7.
We thank the referee for the valuable remark. We have moved Fig EV5E to the main Figure: Fig 8G.

Also, the description in methods on how the lariat-specific reads were identified is not fully comprehensible. While the authors refer to Pineda and Bradley (2018) for how this analysis was executed and describe how reads mapping to both 5'- and 3'-splice sites were identified, it is difficult to follow their logic as to how the split-read alignments were determined to originate from lariats. Perhaps, a schematic summarizing their approach can be added to this figure panel.
Unfortunately, we were unable to use or reference any R package for lariat detection, as such a package is not currently available. Instead, we followed the method outlined in the Pineda and Bradley paper. Our description of the lariat detection process is already quite comprehensive, exceeding the level of detail typically found. However, we have made further enhancements to our description of the method and have also provided a detailed illustration of the lariat analysis in Figure EV6.

Another major issue the authors need to properly acknowledge is the recently published article by Townley et al (2023), which describes many similar findings regarding MPLKIP function alongside DBR1 in splicing, higher lariat accumulation upon MPLKIP deficiency, and the gene-length dependent effect of this deficiency on gene expression. What differentiates the two studies is that Townley et al mainly employ transformed human cell lines or mouse models unlike this study which uses a more disease-relevant in vitro cell model. A discussion of the similar findings from this parallel work in Discussion is warranted. In my opinion, such an open acknowledgement and discussion of similar conclusions of the two studies should not lessen the impact of findings described here although I am not familiar with the journal policy regarding the impact of a competing publication with similar results on the novelty of the work under review.
We have cited the Townley et al. paper already twice in our manuscript, which was initially suggested by reviewer #1. While there are some overlapping results, the majority of our findings are complementary to theirs. The discussion section of our manuscript is already extensive, and the editor has agreed that no alterations are necessary; it remains suitable in its current form. Nevertheless, to address this matter, we have added the following sentence to the end of the discussion: "*During the review process of our manuscript, another paper was published that also describes certain findings related to MPLKIP's role in RNA processing (splicing) and lariat turnover (Townley et al., 2023).*"

Lastly, it is quite easy to spot the uncanny similarity in the lariat read quantification strategy described by the authors in the revised manuscript and that described by Townley et al, although both base this analysis on Pineda and Bradley (2018).
We do not fully understand the qualification "uncanny similarity" between our lariat read quantification strategy and that described by Townley et al., as both analyses were based on the same procedure described by Pineda and Bradley. We are also somewhat perplexed by the use of the term "uncanny" in this context, as we independently and unbiasedly adopted the same analytical approach to investigate a shared biological function. Rather than characterizing it as "uncanny," we view this as a valuable illustration of independent confirmation and also in line with other published data to which we cite in our manuscript.

Although the authors acknowledge that enrichment of NMD and SRP pathways in GO term analysis is mainly driven by ribosome components, they still describe alteration of NMD as a potential mechanism whereby MPLKIP deficient cells attempt to achieve homoeostasis in gene expression (Discussion, page 17). This is misleading and should be corrected. Instead, the focus can be alteration of ribosomal gene expression, possibly to modulate translation. Also, linking ribosome upregulation to "enhancing the cell's capacity to detect and eliminate improperly spliced RNA" is too speculative.
We thought to include description of biological pathways, unbiasedly identified by the commonly used String analysis without any intention to be misleading. The discussion concerning the potential upregulation of NMD and SRP pathways was simply aimed at highlighting a potential biological consequence of improperly spliced transcripts, which aligns with findings in other publications on this topic. Such discussions are typically within the scope of the Discussion section, where broader implications of the research are explored.

In response to the reviewer's request, we have extensively acknowledged and discussed that ribosomal proteins were primarily affected by our findings. Following consultation with the editor, we

have now included the statement that this aspect would require further investigation to fully understand its implications.

The authors point about lack of a tight correlation between RNA and protein expression is well-taken. Still, it is worth testing if at least the trend in the effects of gene length on alteration in RNA expression are also mirrored in changes in protein expression. That is, are upregulated proteins in Fig 8B from shorter genes and downregulated proteins encoded from longer genes?
We did perform an analysis specifically examining the correlation between protein expression changes and gene length, and our findings did not reveal a strong, consistent correlation between upregulated/downregulated proteins and gene length for the majority of proteins. While it is possible to identify correlations when selectively focusing on specific genes, we believe that such observations are too speculative and currently not suitable for inclusion in our manuscript. Nevertheless, we will explicitly state the following in the discussion section: "*We also do not observe a clear correlation on the effects of gene length on alteration in RNA expression and changes in protein expression*".

In Discussion, the authors should more clearly explain their reasoning to assume that intron debranching, a step that occurs during or after spliceosome disassembly, affects the splicing process.
We acknowledge that there may still be some remaining unanswered questions after reviewing the manuscript. However, we have attempted to address these questions by providing possible explanations in the discussion section. Specifically, "*It is worth noting that altering the levels of different splicing factors, as observed here for DBR1, the ratio of specific splicing events can be changed. For example, serine-arginine (SR) proteins and heterogeneous nuclear ribonucleoproteins (hnRNPs) can antagonize each other in a concentration-dependent manner. Altered splicing ratios will eventually result in the production of aberrant proteins that will jeopardize cellular homeostasis (Okunola & Krainer, 2009).*" While we understand that additional questions may arise, it's important to note that the discussion section is already quite comprehensive, and we have received feedback from the editor indicating that no further alterations to the discussion are necessary in its current form.

In figure legends, replicates for experiments are described as "technical replicates". Were biological replicates never performed or is there a confusion between the two terms? Biological replication is crucial for robust experimentation. For example, in Fig EV4, to fully demonstrate that the level of DBR1 mRNA is not changing in TTD287IS and TTD289IS biological replicates of the RT-qPCR data will be needed.
We apologize for any confusion regarding the terminology used in the manuscript. It should be clarified that all experiments in our study were conducted using "biological replicates." This terminology has been revised accordingly in the revised manuscript.

Minor points:
In the revised title, wording "correct splicing" is awkward. Also, wouldn't "keratinocyte differentiation" be more appropriate?
We have taken your feedback into consideration and made the necessary revisions. The 'awkward phrase' "correct splicing" has been removed from the title. After consulting with the editor, we have adjusted the title to: "*Trichothiodystrophy-associated MPLKIP maintains DBR1 levels for proper lariat debranching and ectodermal differentiation".*

Fig 5 title: It is not appropriate to use a descriptive adjective like "proper" to describe the effect of MPLKIP absence on DBR1 binding to NTC.
We have removed the descriptive adjective word “proper” from the title of Fig 5.

Fig 4C- both non-CTR samples are labeled as TTD287IS.
We apologize for this sloppiness and have corrected this, indeed one of the non-CTR samples should be labeled as TTD289IS, this has been adopted in the revised Fig 4C.

Pg12- The authors do not show the results of the DEXSeq anywhere in figures or tables. They direct the readers to figure 8b, but that is a volcano plot based on DESeq2 results.
We apologize for this mistake and removed the reference to the figure. The full DEXseq results is described in the main text and not visualized in the figures.

Pg12, line 18- Why figure 4 is referred to here? It does not relate to the results being discussed.
We apologize for this mistake and have changed the referred figure to Figs EV5C and D.

Pg 15-16, in Discussion paragraph starting "Although no clear functional domain...", there is undue emphasis on IDR of MPLKIP although there are no direct experiments performed by the authors in the manuscript. The speculation about two possible functions of MPLKIP can still be made without going into the possibility of phase separation.
We have retained the section on phase separation in the manuscript, following consultation with the editor. We felt it was somewhat restrictive not to be able to explore potential physical protein properties, such as intrinsically disordered regions (IDR), based on the amino acid sequence. It is well-established in multiple papers that IDR is highly correlated with phase separation. We believe that the discussion section is an appropriate place to draw connections between specific properties of the protein under investigation (MPLKIP) and a known biological phenomenon.

It's important to note that we haven't conducted experiments in this specific direction, which is why we again refer to the Townley paper where they investigate the IDR of MPLKIP.

Discussion is too speculative at places (some instances pointed out above), especially when trying to explain the alterations in gene expression identified in the last two figures.
The editor believes our discussion is adequate and fine in its current form. Nevertheless, we hope that by including our additional adjustments, the manuscript is now ready for publication.

18th Sep 2023

Dear Prof. Vermeulen,

We are pleased to inform you that your manuscript is accepted for publication and is now being sent to our publisher to be included in the next available issue of EMBO Molecular Medicine.

Please read below for additional IMPORTANT information regarding your article, its publication and the production process.

Congratulations on your interesting work,

Zeljko Durdevic

Zeljko Durdevic
Editor
EMBO Molecular Medicine

Follow us on Twitter @EmboMolMed
Sign up for eTOCs at embopress.org/alertsfeeds

*** *** *** IMPORTANT INFORMATION *** *** ***

SPEED OF PUBLICATION
The journal aims for rapid publication of papers, using using the advance online publication "Early View" to expedite the process: A properly copy-edited and formatted version will be published as "Early View" after the proofs have been corrected. Please help the Editors and publisher avoid delays by providing e-mail address(es), telephone and fax numbers at which author(s) can be contacted.

Should you be planning a Press Release on your article, please get in contact with embomolmed@wiley.com as early as possible, in order to coordinate publication and release dates.

LICENSE AND PAYMENT:

All articles published in EMBO Molecular Medicine are fully open access: immediately and freely available to read, download and share.

EMBO Molecular Medicine charges an article processing charge (APC) to cover the publication costs. You, as the corresponding author for this manuscript, should have already received a quote with the article processing fee separately. Please let us know in case this quote has not been received.

Once your article is at Wiley for editorial production you will receive an email from Wiley's Author Services system, which will ask you to log in and will present you with the publication license form for completion. Within the same system the publication fee can be paid by credit card, an invoice, pro forma invoice or purchase order can be requested.

Payment of the publication charge and the signed Open Access Agreement form must be received before the article can be published online.

PROOFS

You will receive the proofs by e-mail approximately 2 weeks after all relevant files have been sent o our Production Office. Please return them within 48 hours and if there should be any problems, please contact the production office at embopressproduction@wiley.com.

Please inform us if there is likely to be any difficulty in reaching you at the above address at that time. Failure to meet our

deadlines may result in a delay of publication.

All further communications concerning your paper proofs should quote reference number EMM-2023-17973-V3 and be directed to the production office at embopressproduction@wiley.com.

Thank you,

Zeljko Durdevic
Editor
EMBO Molecular Medicine

EMBO Press Author Checklist

Corresponding Author Name: Wim Vermeulen
Journal Submitted to: EMBO Molecular Medicine
Manuscript Number: EMM-2023-17973

USEFUL LINKS FOR COMPLETING THIS FORM
The EMBO Journal - Author Guidelines
EMBO Reports - Author Guidelines
Molecular Systems Biology - Author Guidelines
EMBO Molecular Medicine - Author Guidelines

Reporting Checklist for Life Science Articles (updated January
This checklist is adapted from Materials Design Analysis Reporting (MDAR) Checklist for Authors. MDAR establishes a minimum set of requirements in transparent reporting in the life sciences (see Statement of Task: 10.31222/osf.io/9sm4x). Please follow the journal's guidelines in preparing your
Please note that a copy of this checklist will be published alongside your article.

Abridged guidelines for figures
1. Data
The data shown in figures should satisfy the following conditions:
- the data were obtained and processed according to the field's best practice and are presented to reflect the results of the experiments in an accurate and unbiased manner.
- ideally, figure panels should include only measurements that are directly comparable to each other and obtained with the same assay.
- plots include clearly labeled error bars for independent experiments and sample sizes. Unless justified, error bars should not be shown for technical
- if n<5, the individual data points from each experiment should be plotted. Any statistical test employed should be justified.
- Source Data should be included to report the data underlying figures according to the guidelines set out in the authorship guidelines on Data

2. Captions
Each figure caption should contain the following information, for each panel where they are relevant:
- a specification of the experimental system investigated (eg cell line, species name).
- the assay(s) and method(s) used to carry out the reported observations and measurements.
- an explicit mention of the biological and chemical entity(ies) that are being measured.
- an explicit mention of the biological and chemical entity(ies) that are altered/varied/perturbed in a controlled manner.
- the exact sample size (n) for each experimental group/condition, given as a number, not a range;
- a description of the sample collection allowing the reader to understand whether the samples represent technical or biological replicates (including how many animals, litters, cultures, etc.).
- a statement of how many times the experiment shown was independently replicated in the laboratory.
- definitions of statistical methods and measures:
  - common tests, such as t-test (please specify whether paired vs. unpaired), simple χ2 tests, Wilcoxon and Mann-Whitney tests, can be unambiguously identified by name only, but more complex techniques should be described in the methods section;
  - are tests one-sided or two-sided?
  - are there adjustments for multiple comparisons?
  - exact statistical test results, e.g., P values = x but not P values < x;
  - definition of 'center values' as median or average;
  - definition of error bars as s.d. or s.e.m.

Please complete ALL of the questions below.
Select "Not Applicable" only when the requested information is not relevant for your study.

Materials

Newly Created Materials	Information included in the manuscript?	In which section is the information available? (Reagents and Tools Table, Materials and Methods, Figures, Data Availability Section)
New materials and reagents need to be available; do any restrictions apply?	Yes	Materials and Methods; Data Availability Section

Antibodies	Information included in the manuscript?	In which section is the information available? (Reagents and Tools Table, Materials and Methods, Figures, Data Availability Section)
For **antibodies** provide the following information:<br>- Commercial antibodies: RRID (if possible) or supplier name, catalogue number and or/clone number<br>- Non-commercial: RRID or citation	Yes	Materials and Methods

DNA and RNA sequences	Information included in the manuscript?	In which section is the information available? (Reagents and Tools Table, Materials and Methods, Figures, Data Availability Section)
**Short novel DNA or RNA including primers, probes**: provide the sequences.	Yes	Materials and Methods

Cell materials	Information included in the manuscript?	In which section is the information available? (Reagents and Tools Table, Materials and Methods, Figures, Data Availability Section)
**Cell lines:** Provide species information, strain. Provide accession number in repository **OR** supplier name, catalog number, clone number, and/**OR** RRID.	Yes	Materials and Methods; Figures, Appendix
**Primary cultures:** Provide species, strain, sex of origin, genetic modification status.	Yes	Materials and Methods
Report if the cell lines were recently **authenticated** (e.g., by STR profiling) and tested for mycoplasma contamination.	Yes	Mycoplasma test available upon request

Experimental animals	Information included in the manuscript?	In which section is the information available? (Reagents and Tools Table, Materials and Methods, Figures, Data Availability Section)
**Laboratory animals or Model organisms:** Provide species, strain, sex, age, genetic modification status. Provide accession number in repository **OR** supplier name, catalog number, clone number, **OR** RRID.	Not Applicable	
**Animal observed in or captured from the field:** Provide species, sex, and age where possible.	Not Applicable	
Please detail **housing and husbandry conditions**.	Not Applicable	

Plants and microbes	Information included in the manuscript?	In which section is the information available? (Reagents and Tools Table, Materials and Methods, Figures, Data Availability Section)
**Plants:** provide species and strain, ecotype and cultivar where relevant, unique accession number if available, and source (including location for collected wild specimens).	Not Applicable	
**Microbes:** provide species and strain, unique accession number if available, and source.	Not Applicable	

Human research participants	Information included in the manuscript?	In which section is the information available? (Reagents and Tools Table, Materials and Methods, Figures, Data Availability Section)
If collected and within the bounds of privacy constraints report on age, sex and gender or ethnicity for all study participants.	Yes	Materials and Methods

Core facilities	Information included in the manuscript?	In which section is the information available? (Reagents and Tools Table, Materials and Methods, Figures, Data Availability Section)
If your work benefited from core facilities, was their service mentioned in the acknowledgments section?	Yes	Acknowledgement

Design

Study protocol	Information included in the manuscript?	In which section is the information available? (Reagents and Tools Table, Materials and Methods, Figures, Data Availability Section)
If study protocol has been **pre-registered, provide DOI in the manuscript**. For clinical trials, provide the trial registration number **OR** cite DOI.	Not Applicable	
Report the **clinical trial registration number** (at ClinicalTrials.gov or equivalent), where applicable.	Not Applicable	

Laboratory protocol	Information included in the manuscript?	In which section is the information available? (Reagents and Tools Table, Materials and Methods, Figures, Data Availability Section)
Provide DOI OR other citation details if **external detailed step-by-step protocols** are available.	Not Applicable	

Experimental study design and statistics	Information included in the manuscript?	In which section is the information available? (Reagents and Tools Table, Materials and Methods, Figures, Data Availability Section)
Include a statement about **sample size** estimate even if no statistical methods were used.	Yes	Materials and Methods
Were any steps taken to minimize the effects of subjective bias when allocating animals/samples to treatment (e.g. **randomization procedure**)? If yes, have they been described?	Not Applicable	Materials and Methods
Include a statement about **blinding** even if no blinding was done.	Not Applicable	
Describe **inclusion/exclusion criteria** if samples or animals were excluded from the analysis. Were the criteria pre-established?<br><br>If sample or data points were omitted from analysis, report if this was due to attrition or intentional exclusion and provide justification.	Not Applicable	
For every figure, are **statistical tests** justified as appropriate? Do the data meet the assumptions of the tests (e.g., normal distribution)? Describe any methods used to assess it. Is there an estimate of variation within each group of data? Is the variance similar between the groups that are being statistically compared?	Yes	Materials and Methods; Legends, Figures

Sample definition and in-laboratory replication	Information included in the manuscript?	In which section is the information available? (Reagents and Tools Table, Materials and Methods, Figures, Data Availability Section)
In the figure legends: state number of times the experiment was **replicated** in laboratory.	Yes	Figures legends
In the figure legends: define whether data describe **technical or biological replicates**.	Yes	Figures legends

Ethics

Ethics	Information included in the manuscript?	In which section is the information available? (Reagents and Tools Table, Materials and Methods, Figures, Data Availability Section)
Studies involving **human participants**: State details of **authority granting ethics approval** (IRB or equivalent committee(s), provide reference number for approval.	Not Applicable	
Studies involving **human participants**: Include a statement confirming that **informed consent** was obtained from all subjects and that the experiments conformed to the principles set out in the WMA Declaration of Helsinki and the Department of Health and Human Services Belmont Report.	Yes	Materials and Methods
Studies involving **human participants: For** publication of **patient photos**, include a statement confirming that consent to publish was obtained.	Yes	Materials and Methods
Studies involving experimental **animals**: State details of **authority granting ethics approval** (IRB or equivalent committee(s), provide reference number for approval. Include a statement of compliance with ethical regulations.	Not Applicable	
Studies involving **specimen and field samples:** State if relevant **permits** obtained, provide details of authority approving study; if none were required, explain why.	Not Applicable	

Dual Use Research of Concern (DURC)	Information included in the manuscript?	In which section is the information available? (Reagents and Tools Table, Materials and Methods, Figures, Data Availability Section)
Could your study fall under dual use research restrictions? Please check biosecurity documents and list of **select agents and toxins** (CDC): https://www.selectagents.gov/sat/list.htm	Not Applicable	
If you used a select agent, is the security level of the lab appropriate and reported in the manuscript?	Not Applicable	
If a study is subject to dual use research of concern regulations, is the name of the **authority granting approval and reference number** for the regulatory approval provided in the manuscript?	Not Applicable	

Reporting

The MDAR framework recommends adoption of discipline-specific guidelines, established and endorsed through community initiatives. Journals have their own policy about requiring specific guidelines and recommendations to complement MDAR.

Adherence to community standards	Information included in the manuscript?	In which section is the information available? (Reagents and Tools Table, Materials and Methods, Figures, Data Availability Section)
State if relevant guidelines or checklists (e.g., **ICMJE, MIBBI, ARRIVE, PRISMA**) have been followed or provided.	Not Applicable	
For **tumor marker prognostic studies,** we recommend that you follow the **REMARK** reporting guidelines (see link list at top right). See author guidelines, under 'Reporting Guidelines'. Please confirm you have followed these guidelines.	Not Applicable	
For **phase II and III randomized controlled trials**, please refer to the **CONSORT** flow diagram (see link list at top right) and submit the CONSORT checklist (see link list at top right) with your submission. See author guidelines, under 'Reporting Guidelines'. Please confirm you have submitted this list.	Not Applicable	

Data Availability

Data availability	Information included in the manuscript?	In which section is the information available? (Reagents and Tools Table, Materials and Methods, Figures, Data Availability Section)
Have **primary datasets** been deposited according to the journal's guidelines (see 'Data Deposition' section) and the respective accession numbers provided in the Data Availability Section?	Yes	Data Availability Section, Materials and Methods
Were **human clinical and genomic datasets** deposited in a public access-controlled repository in accordance to ethical obligations to the patients and to the applicable consent agreement?	Yes	Data Availability Section, Materials and Methods
Are **computational models** that are central and integral to a study available without restrictions in a machine-readable form? Were the relevant accession numbers or links provided?	Not Applicable	
If publicly available data were reused, provide the respective **data citations in the reference list**.	Not Applicable	
